# Exploring Triazole-Connected Steroid-Pyrimidine Hybrids: Synthesis, Spectroscopic Characterization, and Biological Assessment

**DOI:** 10.1021/acsomega.4c04800

**Published:** 2024-08-29

**Authors:** Anna Kawka, Damian Nowak, Hanna Koenig, Tomasz Pospieszny

**Affiliations:** † Department of Bioactive Products, Faculty of Chemistry, Adam Mickiewicz University, Uniwersytetu Poznańskiego 8 Street, 61-614 Poznań, Poland; ‡ Department of Quantum Chemistry, Faculty of Chemistry, Adam Mickiewicz University, Uniwersytetu Poznańskiego 8 Street, 61-614 Poznań, Poland

## Abstract

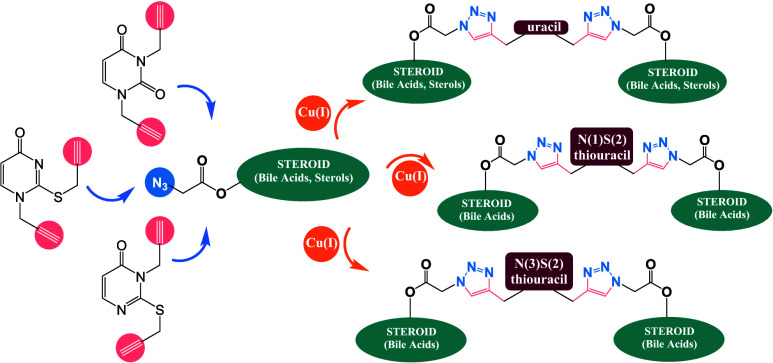

Molecules originating from natural sources are physicochemically and biologically diverse. The conjugation of two active biomolecules has become the foundation for medical and pharmaceutical sciences. An effective synthesis of 11 new steroid-pyrimidine conjugates containing 1,2,3-triazole rings was carried out. The group of 3α–OH bile acids (lithocholic, deoxycholic, cholic) and 3β–OH sterols (cholesterol, cholestanol) were respectively modified to azidoacetates. 2-thiouracil was converted into N(1)S and N(3)S dipropargyl derivatives. Azide–alkyne cycloaddition in the presence of copper(I) of the obtained compounds led to the preparation of 1,2,3-triazole derivatives. Based on a series of spectroscopic (^1^H NMR, ^13^C NMR, Fourier-transform infrared (FT-IR)), spectrometric analyses (Electrospray ionization-mass spectrometry (ESI-MS), electron impact-mass spectrometry (EI-MS)), and semiempirical calculations, the structures of all compounds were confirmed. *In silico* biological tests and molecular docking (for domain 1KZN, 2H94, 5V5Z, 1EZF, 2Q85) were performed for selected compounds. The tests performed indicate the theoretical antimicrobial potential of the obtained ligands.

## 1 Introduction

Two highly significant classes of natural compounds, pyrimidine bases and steroids, are pivotal in numerous biological processes. Among these, thio derivatives of pyrimidine bases such as 2-thiouracil emerge as noteworthy constituents of t-RNA.^1,2^ Beyond their role in biological systems, these compounds have made substantial contributions to the realms of pharmacology, medicine, and medicinal chemistry. Their *S*-, *N*-, or *S*, *N*-disubstituted analogues have displayed notable therapeutic potential, mainly exhibiting antiviral, antithyroid, and antitumor activities.^3−6^ They have also been identified as valuable in biosensing applications and radioprotectors.^7,8^ Nucleobases have been found to have broad applications as precursors capable of forming strong hydrogen bonds in the design of new biomaterials or complex nanostructures.^9,10^

**Scheme 1:**
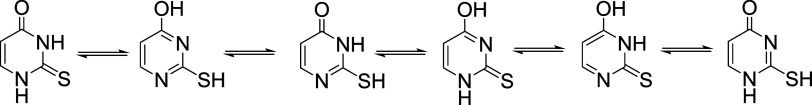
Selected Forms of 2-Thiouracil Tautomerization

The prototropic tautomerism exhibited by thio derivatives of pyrimidine bases has garnered significant interest (Scheme 1). The equilibrium between tautomeric forms, particularly in the case of 2-thiouracil, plays a pivotal role in dictating its chemoselectivity and regioselectivity. Moreover, this equilibrium is intricately influenced by factors such as temperature and the physical state of the compound, whether in solution or the solid phase.^11^

Steroids are essential for medicinal chemistry because they involve all metabolic pathways. The rigid skeleton of cyclopentaneperhydrophenanthrene provides steroid conjugates with the proper arrangement of other molecules and several physicochemical properties.^12^ Bile acids act as an emulsifier, helping with the digestion and absorption of fats, while sterols such as cholesterol are essential for cell structure and the biosynthesis of steroid hormones.^13,14^ Modifications of functional groups such as 3α–OH, 7α–OH, 12α–OH or 3β–OH and amphiphilic properties resulted in their wide use in bioorganic synthesis.^15^ Their 1,2,3–triazole derivatives with anticancer properties are particularly important.^16,17^ These compounds can inhibit the growth of cancer cells through various mechanisms, such as induction of apoptosis, blocking DNA synthesis, or inhibiting angiogenesis.^18−20^ Some studies suggest that bile acid derivatives with 1,2,3-triazole systems may have antiviral activity. They may inhibit viral replication or penetration into host cells.^21,22^ Moreover, they have been used as potential drugs in treating cholestasis and research tools for studying the mechanisms of drug action, especially in the context of interactions with cell receptors and signaling pathways.^23−25^

The main route for the synthesis of 1,2,3–triazole derivatives is the “click” chemistry reaction. This technique involves the rapid, efficient, and specific formation of new chemical bonds between two reactants.^26^ Typically, 1,3–dipolar reactions are used, such as the reactions of azides with alkynes in the presence of copper(I) ions, which lead to triazole linkers.^27^ This method is beneficial in supramolecular chemistry, biochemistry, and nanotechnology, as well as in creating materials with advanced properties. Its advantages are ease and versatility, high reaction efficiency, and minor byproducts.^28,29^

The combination of steroids with pyrimidines can lead to the creation of compounds with unique biological activity.^30^ Conjugation of a carrier molecule with a biologically active substance brings many benefits, such as nontoxicity, minimizing side effects, and overcoming drug resistance of the target cell.^31^ These compounds may have therapeutic, anticancer, antiviral, anti-inflammatory, or neuroprotective effects.^32−35^ Steroid-pyrimidine conjugates may be more stable compared to uncombined components. Bile acid and uracil conjugates are being investigated for their potential anticancer effects. Research suggests that these compounds may have antiproliferative and apoptotic activity against cancer cells, which opens the possibility of their use in anticancer therapy.^36−38^ Bile acid and uracil conjugates can be carriers in gene therapy. Thanks to their ability to bind to deoxyribonucleic acid (DNA), they can deliver genetic material to target cells, which opens the possibility of using them to treat genetic and cancer diseases. These are promising compounds with potential therapeutic applications in oncology, hepatology, virology, and gene therapy.^39−42^

## 2 Results and Discussion

### 2.1 Synthesis

The studies describe efficient syntheses of obtaining new steroid-pyridine bioconjugates linked by 1,2,3–triazole rings. Bile acids (lithocholic, deoxycholic, cholic), sterols (cholesterol, cholestanol) with appropriately modified 3α– or 3β–OCOCH_2_N_3_ groups, such as uracil and 2-thiouracil dipropargyl were used as a reactant. According to the literature, the synthesis of two propargyl derivatives of 2-thiouracil (*N*(1)*S* and *N*(3)*S*), as well as 11 new bile/sterol–pyrimidine conjugates containing 1,2,3-triazole systems, has not been described so far. The created compounds align with the modern trend of synthesis of macrocyclic systems, which are the foundation in the search for new structures with biological activity.

**Scheme 2:**
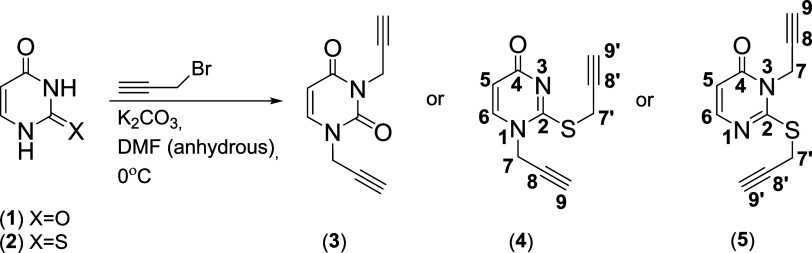
Synthesis of Uracil (**3**) and 2-Thiouracil (**4**–**5**) Propargyl Derivatives

**Scheme 3:**
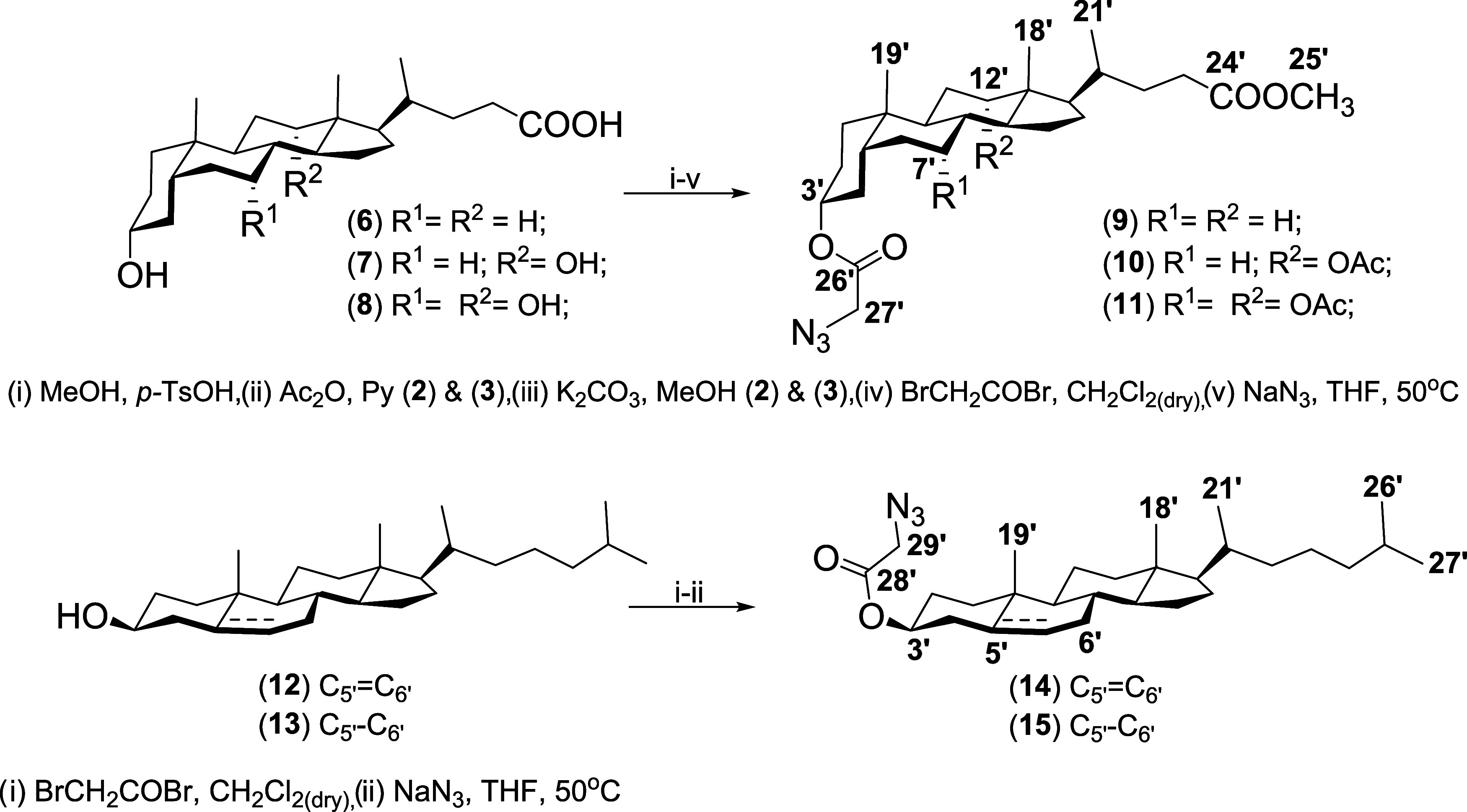
Synthesis of Substituted Derivatives of Methyl Esters of Bile Acids (**9**–**11**) and Sterols (**14**–**15**)

**Scheme 4:**
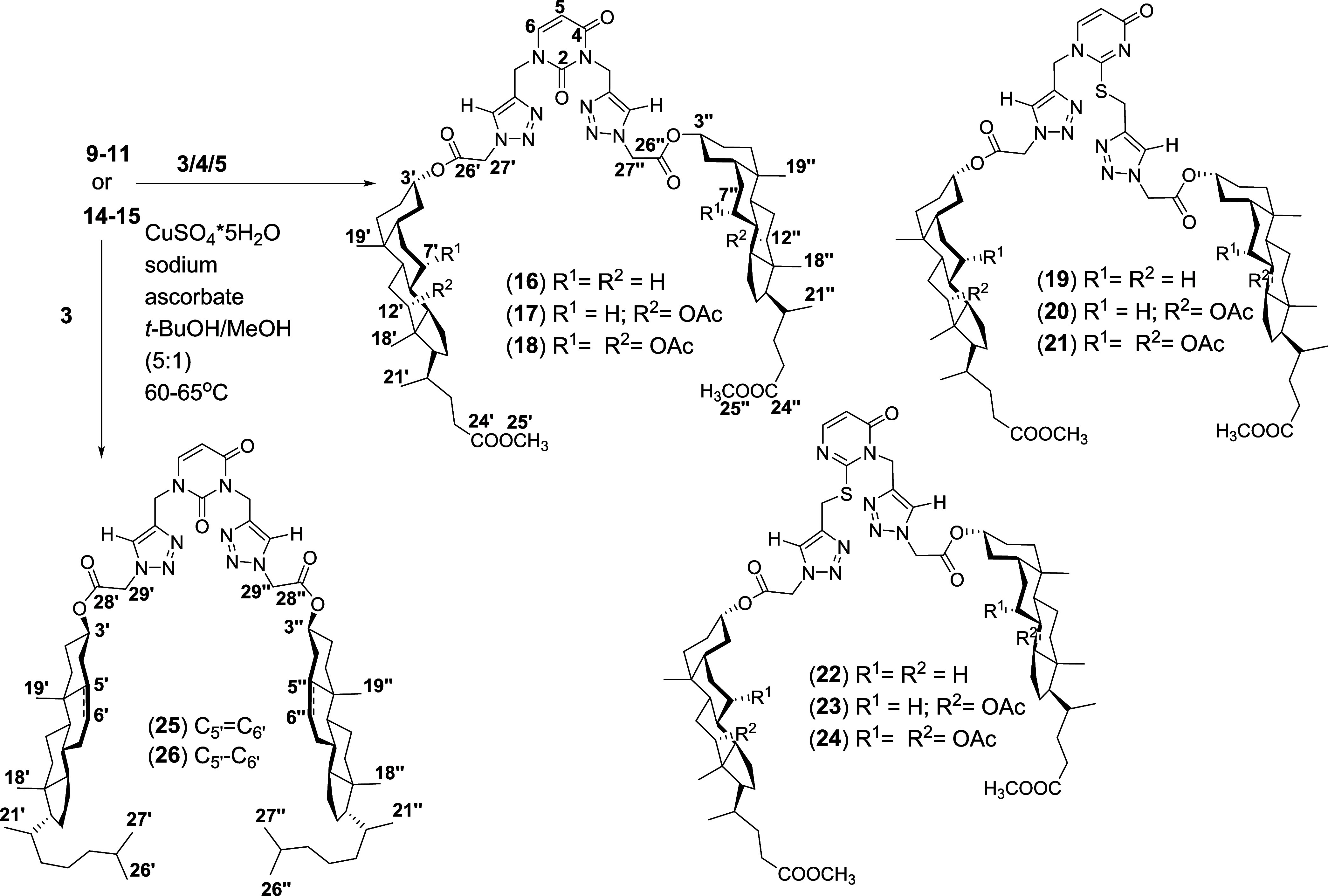
Synthesis of Steroids–pyrimidine Conjugates (**16**–**26**)

The structures of two propargyl disubstituted derivatives of 2-thiouracil (**4**) and (**5**), as well as all synthesized conjugates (**16**–**26**), were determined based on their ^1^H and ^13^C NMR, Fourier-transform infrared (FT-IR), electrospray ionization-mass spectrometry (ESI-MS), and electron impact-mass spectrometry (EI-MS) spectra. Moreover, the PM5 calculation method was performed for all compounds. The syntheses of substrates (**3**–**5**), (**9**–**11**), (**14**–**15**), and conjugates (**16**–**26**) are shown in Schemes 2, 3, and 4, respectively.

### 2.2 Spectroscopic Characteristics

The structural characterization of all synthesized compounds was accomplished by analyzing their ^1^H and ^13^C NMR, FT-IR, ESI-MS, and EI-MS spectra. In addition, PM5 calculations were conducted for each compound further to explore their properties and characteristics.^43−45^

The six substrates: methyl 3α-azidoacetoxy-5β-cholan-24-oate (**9**), methyl 3α-azidoacetoxy-12α-acetoxy-5β-cholan-24-oate (**10**), methyl 3α-azidoacetoxy-7α,12α-diacetoxy-5β-cholan-24-oate (**11**), cholester-3β-yl 2-azidoacetate (**14**), 5α-cholestan-3α-yl azidoethanoate (**15**) and *N*1*, N*3-bis(prop-2-yne-1-yl)uracil (**3**) have been described and characterized in the literature.^46−48^

**Figure 1:**
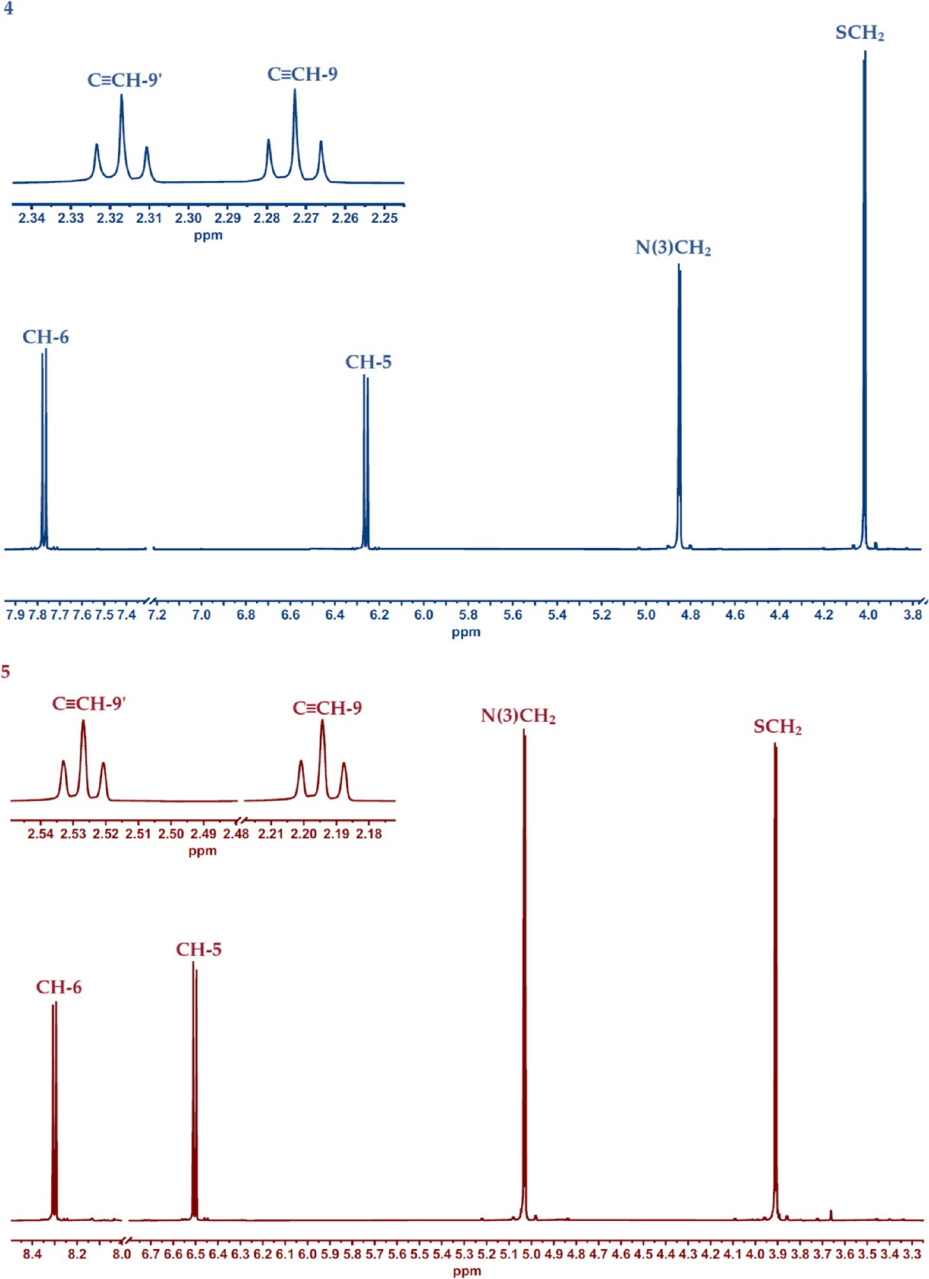
Spectra ^1^H NMR of new compounds (**4**) and (**5**).

In the ^1^H NMR spectrum of dipropargyl derivatives of 2-thiouracil (**4**, **5**), characteristic doublets were observed at 8.30 and 7.77 ppm from CH-6 protons and at 6.50 and 6.26 from CH-5 (Figure 1). Signals from CH_2_–N(1) protons appear as double singlets at 5.03 and 4.85 ppm, similar to CH_2_–S protons at 4.02 and 3.91 ppm. In the 2.53–2.19 ppm range, characteristic triplets originating from C≡CH protons were observed.

**Figure 2:**
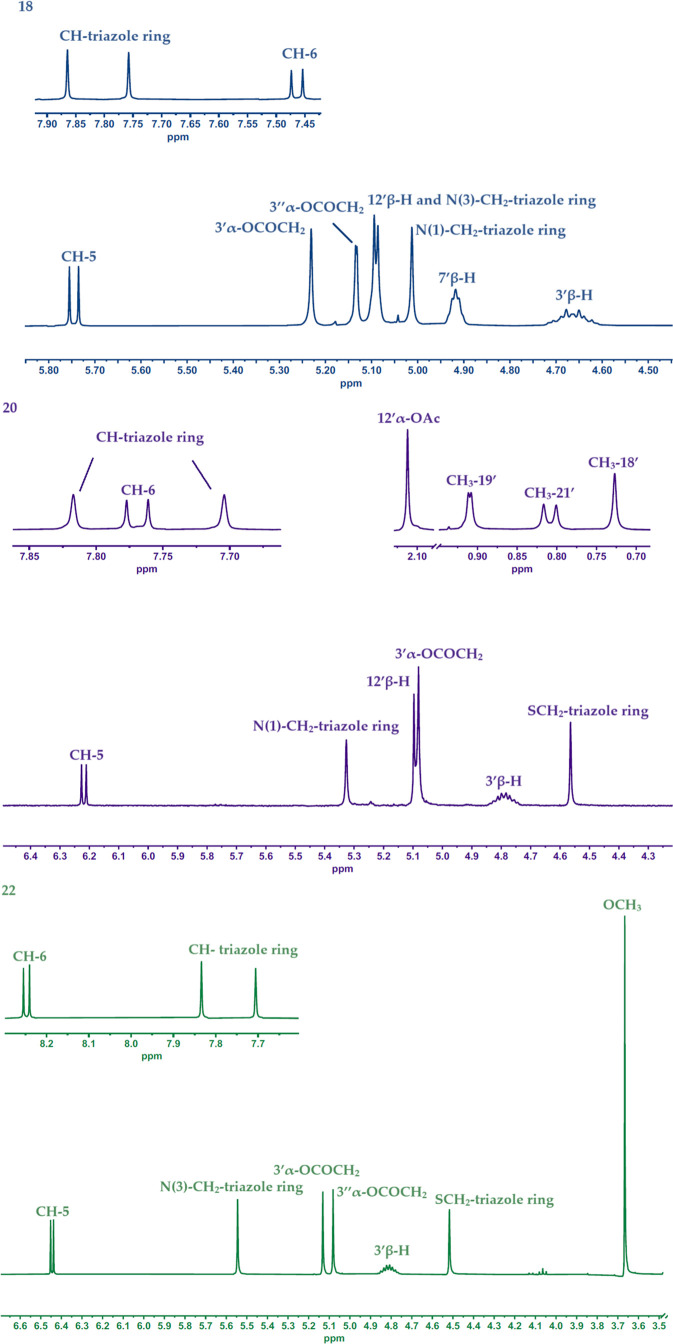
Diagnostic signals in spectra ^1^H NMR conjugates (**18**, **20**, **22**).

Diagnostic signals from protons from 1,2,3-triazole rings were observed for all conjugates (**16**–**26**) as singlets in the 7.90–7.70 ppm range (Figure 2). Characteristic doublets from protons at CH-6 and CH-5 were observed at 8.26–8.25 and 6.45 ppm (for compounds **22**–**24**), 7.77–7.75 and 6.22–6.20 ppm (for compounds **19**–**21**), 7.46–7.45 and 5.75–5.74 ppm (for compounds **16**–**18**, **25**, **26**). Protons present in the methylene groups N–CH_2_–triazole ring and S-CH_2_–triazole ring give singlets with values of 5.54–5.52, respectively (N(3)), 5.34–5.33 (N(1)) and 4.56–4.50 ppm. Signals appeared in the ^1^H NMR spectra of steroid–uracil conjugates (**16**–**18**, **25**–**26**) at 5.25–5.23 and 5.13–5.11 ppm from protons of the CH_2_ methylene groups connecting the steroid skeleton with the triazole ring. For compounds (**19**–**24**), similar singlets were observed at 5.15–5.08 ppm. In the ^1^H NMR spectra of all compounds (**16**–**26**), there are characteristic multiplets in the 4.86–4.61 ppm belonging to 3′α-H and 3′β-H. Additionally, in the case of conjugates (**17**–**18**), (**20**–**21**), (**23**–**24**), a singlet appears from the 12′β-H proton at 5.11–5.09, while for (**18**), (**21**), (**24**) multiplets originating with the 7′β-H proton at 4.93–4.91 ppm. A cholesterol derivative (**26**) diagnostic is a broad singlet for C6′-H at 5.38 ppm. The proton spectra of all bile acid–pyrimidine compounds are characterized by a singlet at 3.67–3.65 ppm coming from protons from the ester group CH_3_-25′. Moreover, for derivatives of deoxycholic and cholic acids, characteristic singlets from hydrogens present in the 7′α–OCOCH_3_ and 12′α–OCOCH_3_ groups can be observed at 2.15 and 2.11–2.09 ppm, respectively. In the ^1^H NMR spectra of all compounds (**16**–**26**), signals coming at the hydrogens from the methyl groups CH_3_-18′, CH_3_-19′, and CH_3_-21′ were observed at 0.73–0.65, 1.04–0.90, and 0.93–0.91 ppm, respectively. Moreover, for sterol derivatives (**25**–**26**), doublets appear in the range of 0.86–0.85 ppm, characteristic of the CH_3_-26′ and CH_3_-27′ groups.

The ^13^C NMR spectra of compounds (**4**) and (**5**) show signals from carbons C(4)=O, C(2)-S, C(6)=(C5), at 169.8–160.7, 167.5–160.7, 157.8–151.9 and 110.8–104.1 ppm, respectively. Signals from C≡C atoms were observed in the 79.7–70.5 ppm range, while signals from carbons from methylene groups CH_2_ were observed in the 54.1–19.4 ppm range.

In the ^13^C NMR spectra of all conjugates (**16**–**26**), signals from carbons from methyl groups C-18′, C-21′, and C-19′ were observed about 12.4–11.8, 18.7–17.5, 23.0–20.8 ppm, and additionally for compounds (**25**, **26**) at 21.2–19.2 ppm from atoms C-26′ and C-27′. However, carbon atoms in the carbonyl group in positions 3′α (or 3′β)-OC=O resonate in the 165.7–165.4 ppm range. The carbon atoms of the 12′α-OC=O steroid skeleton gave signals in the range of 170.5–170.4 ppm. However, the carbon of the 7′α-OC=O gave a signal at 170.5–170.2 ppm. The signals of C(24′)=O, C(4)=O, and C(2)-S appeared in the range of 174.7–174.5, 170.4–162.0, and 168.1–151.1 ppm, respectively. Moreover, unsaturated carbon atoms at the C(5)=C(6) bond can be seen at 111.0–102.0 and 157.6–142.4 ppm. However, the diagnostic carbon atoms from the triazole ring, such as C=CH, give signals at 145.7–138.8 and 125.4–123.3 ppm, respectively. On the other hand, the methylene carbon atom of the steroid skeleton-CH_2_–triazole ring was observed at 51.2–51.0 ppm.

### 2.3 Infrared Spectroscopy

The most characteristic feature of the FT-IR spectra of compound (**4**, **5**) is the band at 3287–3228 cm^–1^ assigned to the ν(≡C–CH) group. The steroid skeleton is a saturated hydrocarbon, so it does not provide many useful IR features. Stretching solid vibrations of C–H bonds are identified at 2972 and 2927 cm^–1^. Additionally, for synthesis, the new compounds of thiouracil are requisite and analytical bands at 2122 cm^–1^, which are specific attributes of ν(C≡C) group. Moreover, for all products (**16**–**26**) are also observed two characteristic solid bands at 1748–1661 cm^–1^ and 1248–1211 cm^–1^ are assigned respectively to the symmetric group ν(C=O) and ν(C–O). In addition, characteristic stretching vibrations of C–H bonds are present in 2965–2852 cm^–1^.

### 2.4 Electron Impact Mass Spectrometry

**Scheme 5:**
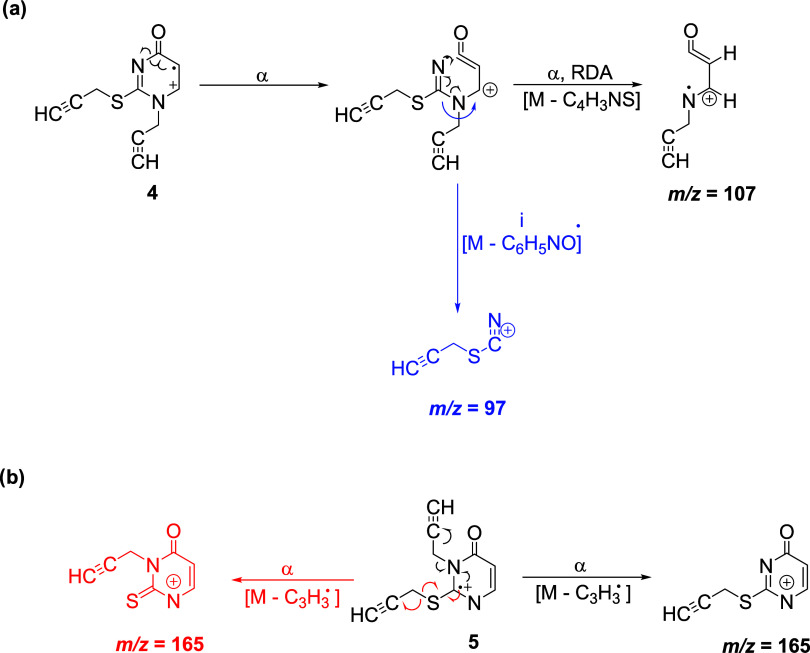
Fragmentation of Compounds (**4**) (a) and (**5**) (b) during EI-MS

In the *N*(1) *S* substitution, there are ions with molecular masses of 107 and 97, which are not present in the *N*(3)*S* substitution spectrum. The presence of the *m*/*z* 165 ion in *N*(3)*S* with an intensity of 100% indicates the possibility of its formation in two ways mass fragmentation (Scheme 5).

### 2.5 Electrospray Ionization

The ESI-MS spectra were acquired using methanol as the solvent. In all instances, the molecular ion [M]^+^ is detected, indicating the presence of a positively charged ion with a proton, alkali metals, or halides in positive ion mode (ES^+^) as well as negative ion mode (ES^–^).

**Figure 3:**
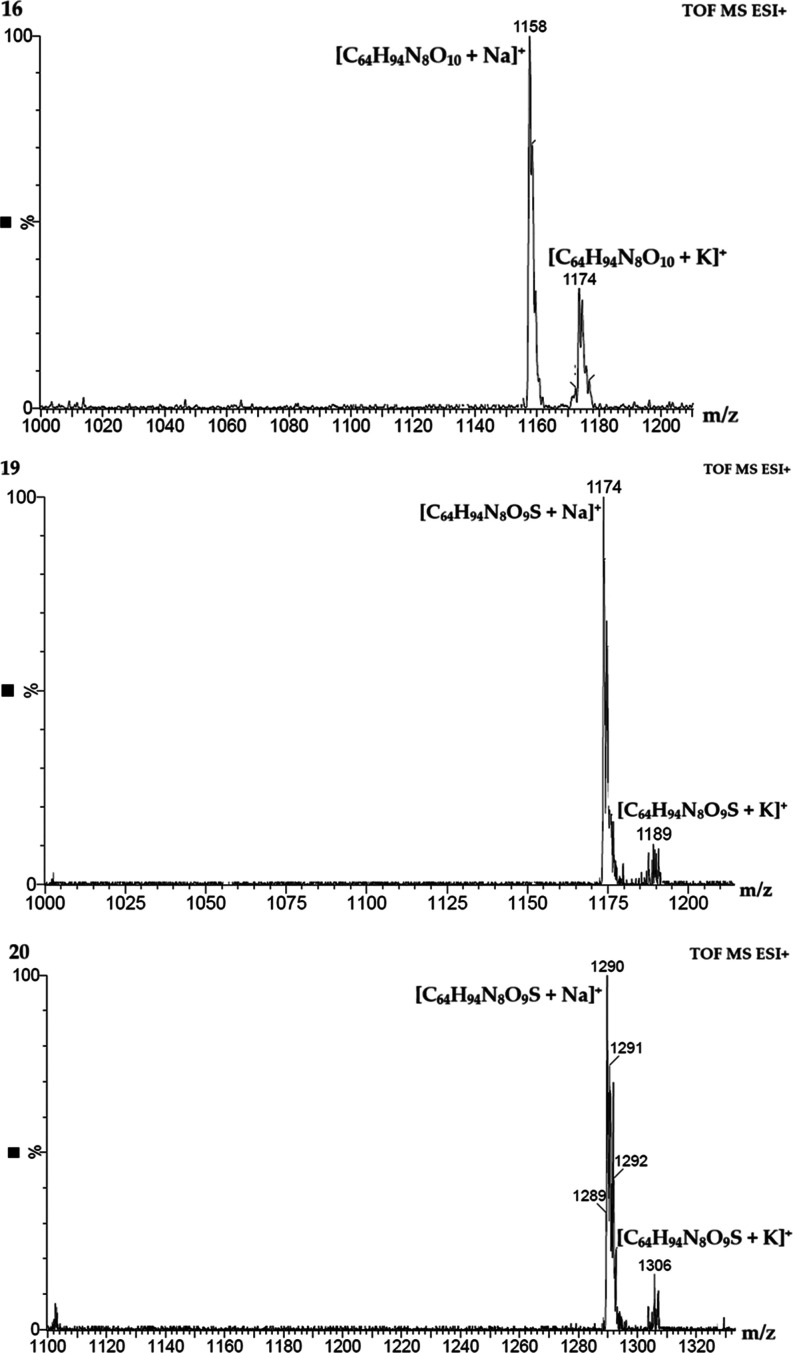
ESI-MS spectra of bioconjugates (**16**), (**19**), and (**20**).

Figure 3 displays the ESI-MS spectra of conjugates **(16**), (**19**), and (**20**). In this spectrum, ion peaks are observed at *m*/*z* at 1174 (20%) [C_64_H_94_N_8_O_10_ + K]^+^ and 1158 (100%) [C_64_H_94_N_8_O_10_ + Na]^+^ (compound **16**), 1189 [C_64_H_94_N_8_O_9_S + K]^+^ (10%) and 1174 (100%) [C_64_H_94_N_8_O_9_S + Na]^+^ (compound **19**), 1306 [C_68_H_98_N_8_O_13_S + K]^+^ and 1290 (100%) [C_68_H_98_N_8_O_13_S + Na]^+^ (compound **20**).

### 2.6 PM5 Calculations

**Figure 4:**
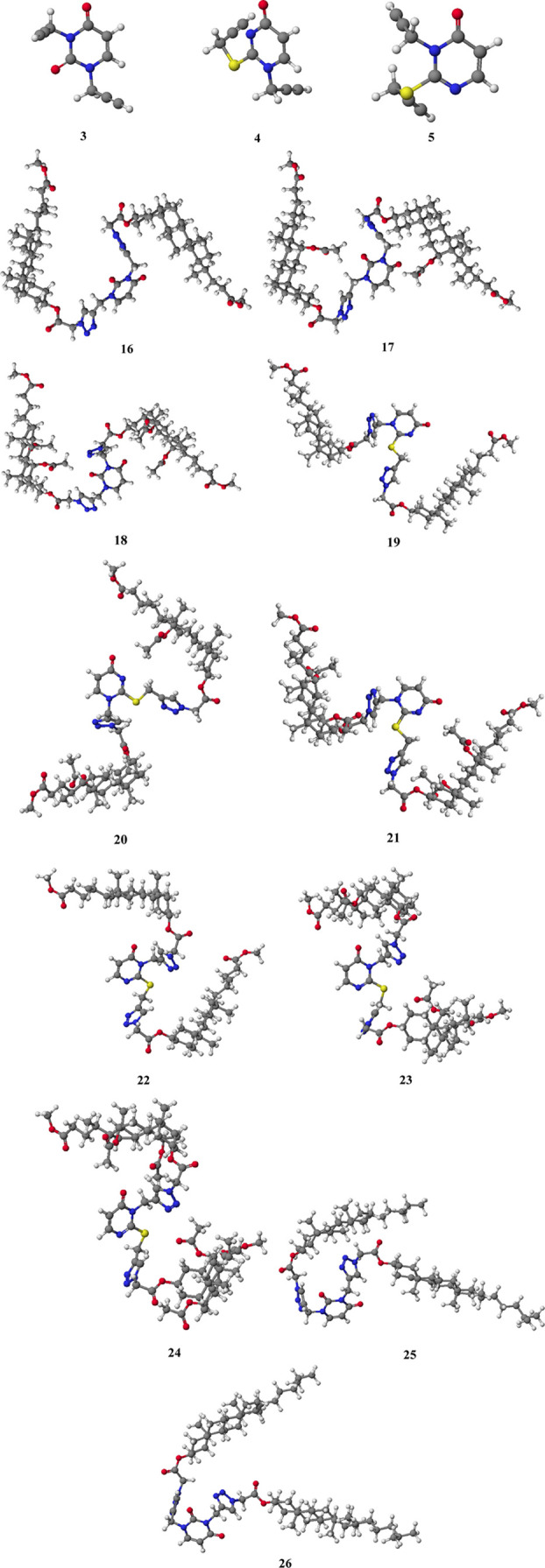
Molecular models of representative compounds (**4**) and (**5**), as well as (**16**–**26**), were calculated by the PM5 method.

The PM5 semiempirical calculations were performed using the WinMopac 2003 program. The final heat of formation (HOF) of compounds (**3**, **4**, **5**) and conjugates (**16**–**26**) is presented in Table 1, and Figure 4 shows their molecular models. Theoretical values of calculations are very often used in comparing crystallographic structures or in determining molecular docking.

**Table 1:** Heat of Formation [kcal/mol] of Compounds (**3**, **4**, **5**) and (**16**–**26**)

compound	heat of formation [kcal/mol]
**3**	28.9829
**4**	97.0622
**5**	90.6298
**16**	–439.5148
**17**	–606.8373
**18**	–784.1348
**19**	–370.6792
**20**	–546.0568
**21**	–712.6079
**22**	–379.0817
**23**	–548.2400
**24**	–712.2926
**25**	–272.6631
**26**	–321.9369

The obtained HOF values indicate that the most stable are triazole systems of cholic acid with uracil or thiouracil (**18**, **21**, **24**). This results from blocked hydroxyl groups in the steroid skeleton at the C-7′ and C-12′ atoms. Acetate groups (7′α-OAc and 12′α-OAc) predispose to the formation of intramolecular interactions, such as hydrogen bonds or electrostatic interactions. Therefore, the obtained compounds can form stable host–guest complexes. The HOF values in each bile acid conjugate increase with decreasing OAc groups. The lowest HOF values are observed for 1,2,3-triazole derivatives of lithocholic acid (**16**, **19**, **22**) and sterols (**25**, **26**). This is due to the lack of additional groups to create stable interactions. Moreover, due to the C5′=C6′ double bond, the cholesterol conjugate (**25**) has a higher HOF value than the cholestanol conjugate (**26**). The positive HOF values for compounds (**3**, **4**, **5**) result from two propargyl groups in each molecule. Alkyne bonds make uracil/thiouracil derivatives the least stable and can be easily modified.

### 2.7 Prediction of Activity Spectra for Substances

The pharmacological activities of the synthesized compounds were determined using the in silico Prediction of Activity Spectra for Substances (PASS) program, which is based on computer analysis of structure–activity relationships. This program uses a heterogeneous training set containing approximately 250,000 biologically active compounds from various chemical series, covering approximately 4500 different types of biological activity. Because PASS only requires the structural formula of a chemical compound to predict, it can be used in the early stages of research. Many examples of successful applications of PASS have led to the development of new pharmacological agents.^49−52^ This program is also helpful in studying the biological activity of secondary metabolites. The present study focused on the activities predicted with the highest probability for a potential compound (focal activities).

Biological activity prediction analysis could only be performed for compounds whose mass does not exceed 1200 g/mol. Therefore, the potential pharmacotherapeutic properties of two new propargyl thiouracil derivatives (**4** and **5**) and conjugates (**16**, **19**, **22**, **25**, **26**) have been described. Table 2 lists the target compounds against which the conjugates show the highest probability of biological activity. According to the research results, the most frequently predicted types of activity for compounds (**4**) and (**5**) are proteasome ATPase inhibitor, electron-transferring-flavoprotein dehydrogenase inhibitor, muramoyltetrapeptide carboxypeptidase inhibitor and chloride peroxidase inhibitor. However, *in silico* tests for the new steroid-pyrimidine conjugates (**16**, **19**, **22**, **25**, **26**) obtained show the highest activity against glyceryl-ether monooxygenase inhibitor, alkylacetylglycerophosphatase inhibitor, antieczematic, cytoprotectant and CYP17 inhibitor. The obtained triazole derivatives show different biological activity than thiouracil substrates. Their predicted effects can be used in the design of hypolipemic, antispot, cholesterol-lowering and even prostate cancer treatments.

**Table 2:** “Probability to be Active” (PA) Values for the Predicted biological Activity of (**4**–**5**), as well as (**16**, **19**, **22**, **25**, **26**)

	compound						
focal predicted activity (PA > 50%) (**4**, **5**) – substrates; (**16**, **19**, **22**, **25**, **26**) – conjugates	**4**	**5**	**16**	**19**	**22**	**25**	**26**
electron-transferring-flavoprotein dehydrogenase inhibitor	64	74	–	–	–	–	–
mannotetraose 2-α-*N*-acetylglucosaminyl transferase inhibitor	59	59	–	–	–	–	–
chloride peroxidase inhibitor	56	56	–	–	–	–	–
proteasome ATPase inhibitor	51	79	–	–	–	–	54
muramoyltetrapeptide carboxypeptidase inhibitor	–	74	–	–	–	–	–
chenodeoxycholoyltaurine hydrolase inhibitor	–	62	–	–	–	–	–
formaldehyde transketolase inhibitor	–	58	–	–	–	–	–
ferredoxin-NAD + reductase inhibitor	–	57	–	–	–	–	–
naphthalene 1,2-dioxygenase inhibitor	–	57	–	–	–	–	–
glutathione thiol esterase inhibitor	–	58	–	–	–	–	–
glyceryl-ether monooxygenase inhibitor	–	52	71	69	69	61	73
alkylacetylglycerophosphatase inhibitor	–	–	68	–	52	–	71
antieczematic	–	–	69	59	–	64	67
cytoprotectant	–	–	61	57	60	–	–
biliary tract disorder treatment	–	–	56	–	–	–	61
dermatologic	–	–	57	52	54	59	57
anti-infertility, female	–	–	53	–	–	58	57
prostate disorders treatment	–	–	53	–	–	58	53
Myc inhibitor	–	–	52	50	50	58	54
antipruritic, allergic	–	–	52	–	–	54	54
CYP17 inhibitor	–	–	–	–	–	65	–
DELTA14-sterol reductase inhibitor	–	–	–	–	–	60	–

### 2.8 Molecular Docking

The molecular docking process started by converting the SMILES^53^ representation of conjugated chemical structures (new ligands) into three-dimensional (3D) structures, and the Gasteiger^54^ charges were added to each new ligand. It was accomplished through the application of OpenBabel tool version 3.1.1.^55,56^ Subsequently, the protein domains corresponding to the PDB^57^ IDs 1EZF(58,59)1KZN,^60,61^2H94,^62,63^2Q85^64,65^ and 5V5Z(66,67) were prepared in accordance with the standard AutoDock tool 1.5.7 scheme.^68^ Molecular dockings were then carried out using AutoDock Vina,^69^ with the specific parameters outlined in Table 3 for each docking search.

**Table 3:** Search Space of Each of the Protein Domains^a^

PDB ID/search parameter	1EZF	1KZN	2H94	2Q85	5V5Z
search size	66,60,64	62, 54, 60	62, 62, 56	60, 50, 50	72, 70, 50
search center	–12.596, 43.347, 32.218	18.320, 30.783, 36.761	25.643, 43.019, 89.938	13.168, 2.780, 2.799	–38.038, – 19.282, 24.569

a The values are given in *x*, *y*, and *z* coordinates and the Angstrom unit.

The determination of molecular docking centers was based on the ligands in the raw PDB files.^70^ UCSF Chimera software, version 1.1670, was employed to visualize the three-dimensional aspects.

### 2.9 Selected Protein Domains

Their specific biological functions guided the selection of protein domains within the physiological system:

- antifungal activity (PDB IDs: 1EZF, 5V5Z)
- antibacterial activity (PDB IDs: 1KZN, 2Q85)
- Both antifungal and antibacterial activity (PDB ID: 2H94)

These proteins belong to different enzyme classes:^71^

- transferases (1EZF) - transfer specific functional groups from one molecule to another
- isomerases (1KZN) - facilitate intramolecular rearrangements within a single molecule
- oxidoreductases (2H94, 2Q85, and 5V5Z) - catalyze oxidation–reduction reactions by transferring electrons between molecules

The protein domain 1EZF(58) corresponds to squalene synthase, which catalyzes squalene production, a precursor to ergosterol, an essential component of fungal cell membranes. Inhibiting squalene synthase disrupts ergosterol biosynthesis, making it a classic target for antifungal drugs. Understanding the structure and function of squalene synthase aids in developing antifungal agents to combat fungal infections.^59,72^

The protein domain 1KZN,^60^ known as gyrase, is crucial in bacterial DNA replication and transcription by catalyzing negative supercoiling of circular DNA. Targeting DNA gyrase with antibacterial agents can lead to bacterial death. Compounds like quinolones, coumarins, and cyclothialidines have been developed to inhibit gyrase, offering potential avenues for antibacterial drug discovery.^61,73^

The protein domain 2H94(62) shows the shape of a human enzyme called Lysine-Specific Demethylase-1 (LSD1). This enzyme helps control how genes are turned on or off by removing certain chemical marks from proteins called histones. These marks are like little tags on the histone tails. Interestingly, the products of this enzyme can also have antifungal properties. Some bacteria and fungi depend on specific chemical reactions for their survival. By interfering with these reactions, the LSD1 enzyme can slow down the growth of these microbes.^63,74^

The protein domain 2Q85(64) corresponds to *Escherichia coli* MurB, associated with antibacterial activity. MurB is an enzyme involved in the peptidoglycan biosynthesis pathway of bacteria, making it a potential target for antibacterial agents. Inhibiting MurB could disrupt bacterial cell wall formation, leading to bacterial death.^65,75,76^

The protein domain 5V5Z(66) represents the structure of CYP51 from the pathogen *Candida albicans*, known as lanosterol 14α-demethylase (LDM). LDM is the target of azole drugs used to treat fungal infections, but their efficacy is limited by drug resistance and suboptimal cure rates, especially in immunocompromised patients. Understanding the structure of CYP51 aids in developing new antifungal agents to combat drug-resistant fungal pathogens and improve treatment outcomes for fungal infections.^67,76,77^

### 2.10 Similarities and Differences between Novel and Endogenous Ligands

To visualize the differences between native ligand and new ligands, the molecular some of QED (Quantitative Estimate of Druglikeness)^78^ descriptors were calculated with RDKit python library,^80^ as shown in Table 4.

**Table 4:** Comparison of Native Ligands and New Ligand Molecular Descriptors

name	molecular weight [g/mol]	HB acceptors	HB donors	polar surface area Å^2^	*a* log *P*	rotatable bonds
1EZF	539	7	3	133.24	1.09	9
1KZN	664	11	4	186.35	2.07	9
2H94	763	20	10	363.61	–0.98	13
2Q85	763	20	10	363.31	–0.98	13
5V5Z	667	9	0	104.70	1.73	11
4	204	3	0	34.89	0.60	3
5	204	3	0	34.89	0.60	3
16	1136	14	0	210.62	9.60	18
17	1252	18	0	263.22	8.69	20
18	1368	22	0	315.82	7.78	22
19	1152	15	0	201.51	11.09	19
20	1268	19	0	254.11	10.17	21
21	1384	23	0	306.71	9.25	23
22	1152	15	0	201.51	11.09	19
23	1268	19	0	254.11	10.17	21
24	1384	23	0	306.71	9.25	23
25	1128	10	0	158.02	13.20	20
26	1132	10	0	158.02	13.36	20

Both the native and new ligands exhibit a wide range of molecular weights. The new ligands have more molecular weight than the native ligands. The number of hydrogen bonds acceptors of new ligands is similar to the number of hydrogen bonds acceptors in native ligands. In the case of hydrogen bonds donors, the new ligands do not have any of them. The polar surface area is generally higher than in the case of new ligands. The lipophilicity is much different for the new ligands exposed to higher alogP values.^79^ It indicates that new ligands are exhibiting much higher lipophilicity compared to native ligands, which may lead to low solubility and poor absorption.^80^ There is also variability in the number of rotatable bonds, meaning that the flexibility of the ligands is different. To conclude, it can be said that the new ligands are not very similar to native ligands. Thus, they can be potent ligands that do good activity.

### 2.11 Molecular Docking

The RMSD (Root-Mean-Square Deviation) values of atomic positions are provided in Table 5. RMSD measures the average distance between the atoms of two superimposed molecules. It is commonly used to quantify the structural similarity between two molecules in space. In the context of molecular docking, RMSD describes how far the predicted pose of a ligand differs from the native pose. Lower RMSD values indicate higher accuracy in pose prediction.

**Table 5:** Quantitative Assessment of the Structural Differences between the Endogenous Ligands Raw Pose and the Endogenous Ligands Predicted Pose through Molecular Docking

PDB ID	1EZF	1KZN	2H94	2Q85	5V5Z
RMSD (Å)	1.30	0.50	0.54	1.54	1.65

Table 6 presents the results of molecular docking studies conducted via AutoDock Vina software.^69^ The five protein domains have been studied. Three of them are related to antibacterial activity (1KZN and 2Q85), one to anticancer (2H94), and the others (1EZF and 5V5Z) are related to antifungal activity.

**Table 6:** Results– Binding Energies (Affinities) are Given in [kcal/mol] Unit

protein domain ID/ligand name	1EZF	1KZN	2H94	2Q85	5V5Z
native	–11.9	–9.1	–14.6	–10.9	–10.5
**4**	–5.7	–5.7	–6.2	–5.9	–5.9
**5**	–6.2	–5.2	–5.6	–5.7	–5.6
**16**	–11.4	–7.1	–9.5	–11.3	–11.3
**17**	–11.4	–8.3	–9.9	–12.7	–10.8
**18**	–11.1	–6.4	–9.6	–9.7	–10.6
**19**	–10.8	–8.0	–10.7	–11.3	–11.2
**20**	–10.9	–7.1	–9.9	–10.7	–10.6
**21**	–10.2	–8.0	–9.3	–11.6	–9.7
**22**	–11.3	–8.8	–10.2	–11.2	–11.9
**23**	–10.9	–7.6	–10.2	–9.5	–10.8
**24**	–10.4	–8.2	–9.3	–10.1	–10.1
**25**	–11.4	–8.7	–10.3	–11.6	–12.1
**26**	–11.6	–7.4	–10.0	–10.8	–11.3

### 2.12 1EZF Protein Domain

**Figure 5:**
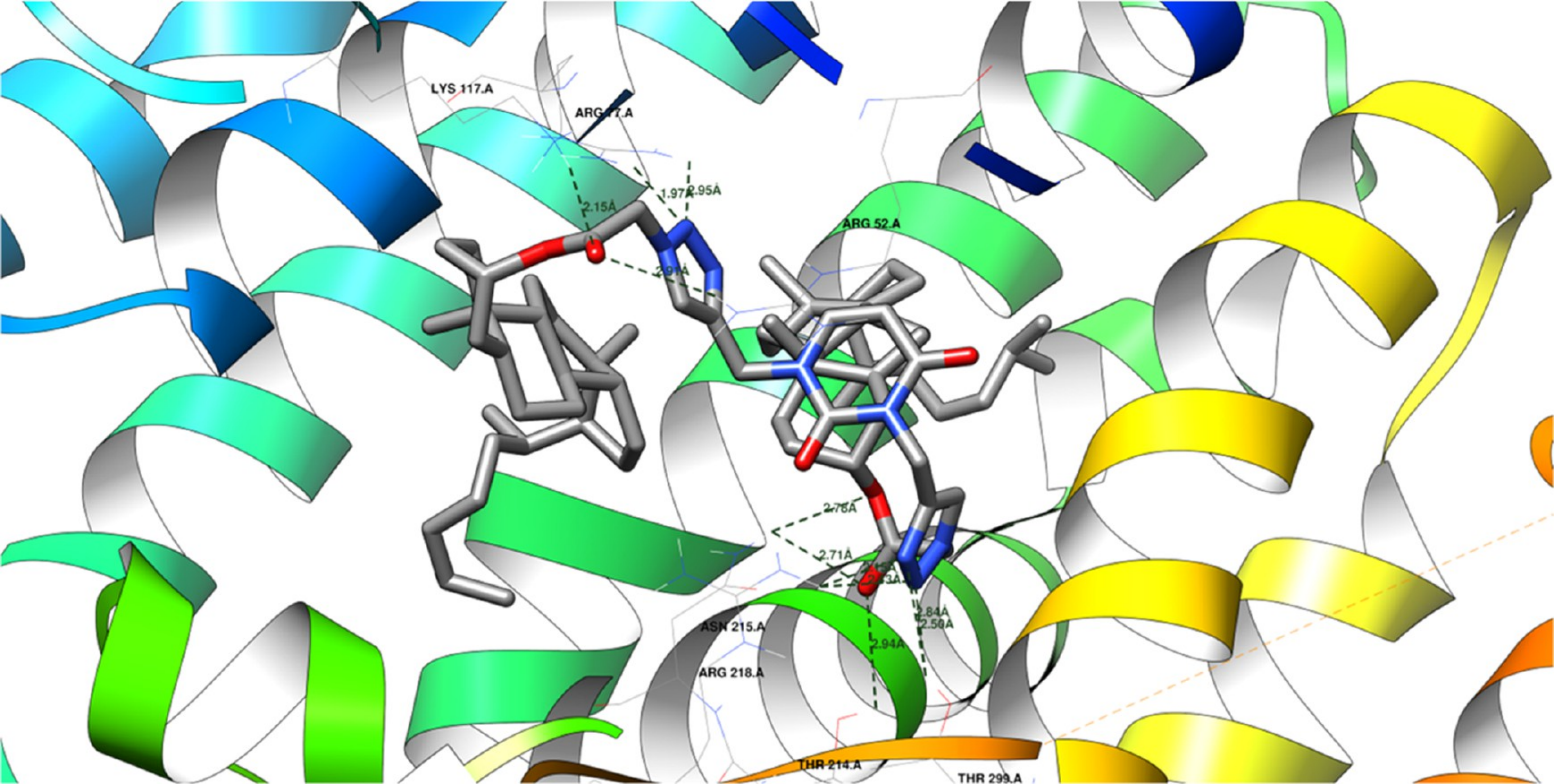
Potential hydrogen bonds between **26** ligand and the 1EZF protein domain.

Regarding antifungal activity, protein domain 1EZF reveals that the newly discovered ligands have affinities comparable to the native ligand’s binding energy. Only in a few cases do the binding energies of new ligands deviate by more than 1 kcal/mol. Nevertheless, their affinities fall within the same range as the native ligand. The RMSD for the native ligand, in this case, is 1.30 Å (Table 5).^81^Figure 5 shows how a protein and a molecule called a **26** ligand might bond together. They could form up to 11 hydrogen bonds, like tiny bridges holding them together. However, some of these bonds compete with each other, meaning only about six can form at the same time.

The **26** ligand can form hydrogen bonds with several specific amino acids in the 1EZF protein domain. These hydrogen bonds help hold the ligand and protein together. The shorter the distance between the atoms involved in a hydrogen bond, the stronger the bond. These hydrogen bonds involve the following atoms and distances:

- Ligand’s keto-ester oxygen atom (acceptor) to LYS 117 A amine (donor): 2.15 Å
- Ligand’s keto-ester oxygen atom (acceptor) to ARG 52 A amine (donor): 2.91 Å
- Ligand’s 1,2,3-triazole nitrogen (acceptor) to ARG 77 A amine (donor): 1.97 Å (more likely) or 2.95 Å
- Ligand’s other ester oxygen atom (acceptor) to ASN 215 A amine (donor): 2.71 or 2.78 Å
- Ligand’s keto-ester oxygen atom (acceptor) to THR 214 A amine (donor): 2.94 Å
- Ligand’s other 1,2,3-triazole nitrogen (acceptor) to ARG 218 A amine (donor): 2.15 or 2.43 Å
- Ligand’s other 1,2,3-triazole nitrogen (acceptor) to THR 299 A amine (donor): 2.50 or 2.84 Å

### 2.13 1KZN Protein Domain

**Figure 6:**
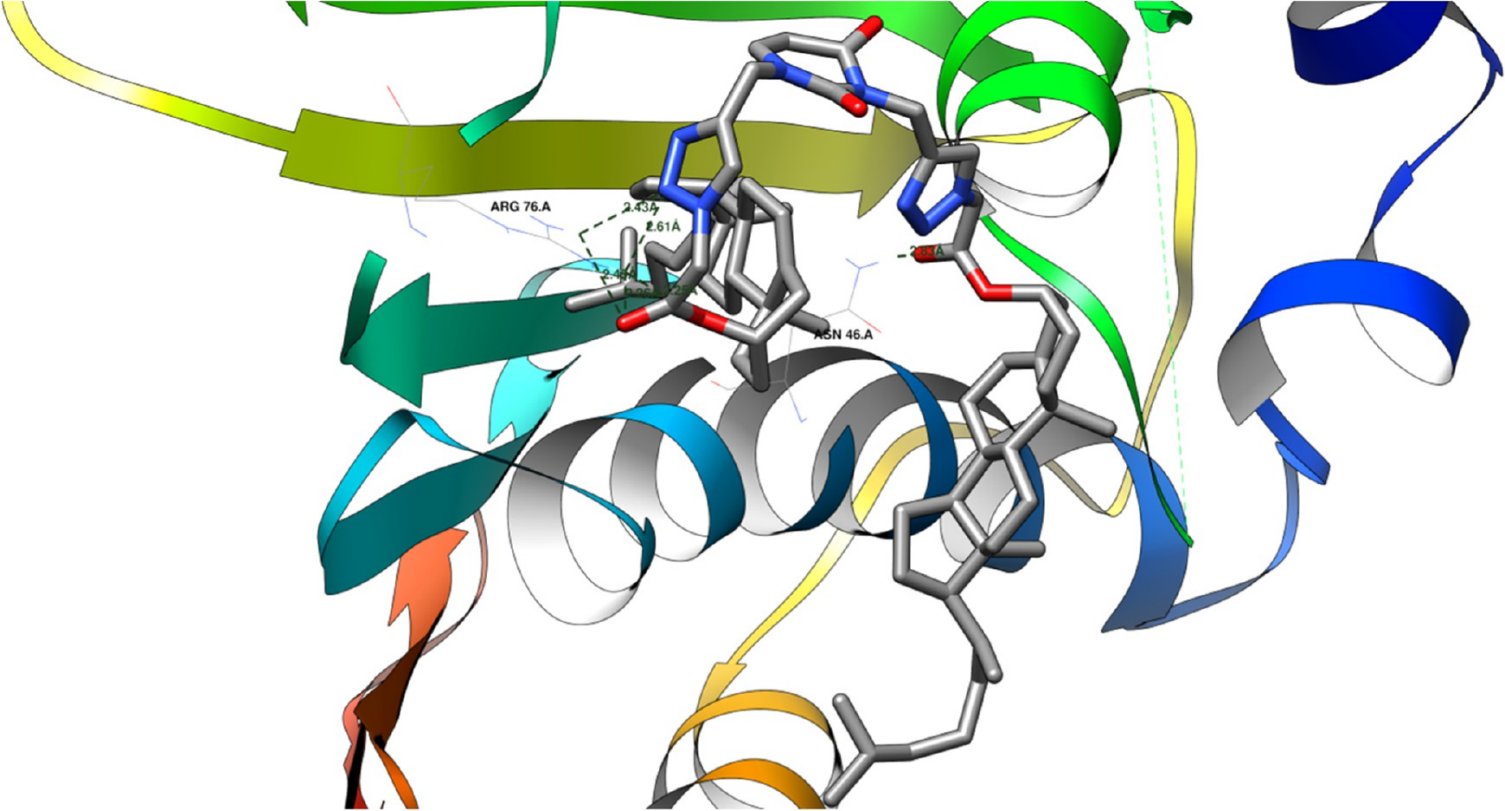
Potential hydrogen bonds between ligand **25** and the 1KZN protein domain.

These ligands exhibit similar or lower binding energies than the native ligand for the 1KZN protein domain. Specifically, ligands such as **17**, **22**, **24**, and **25** demonstrate similar affinities (within ±1 kcal/mol) to the native ligand, while others (**4**, **5**, **16**, **18**, **19**, **20**, **21**, **23** and **26**) exhibit lower affinities. The reduced affinities of these ligands can be attributed to their larger sizes compared to the native ligand. Notably, the root-mean-square deviation (RMSD) between the initial pose of the native ligand and its reduced pose is 0.50 Å (Table 5), indicating an accurate recreation of the native ligand’s binding configuration.^81^Figure 6 shows how a protein and a molecule called a **25** ligand might bond together. They could form up to six hydrogen bonds, like tiny bridges holding them together. However, some of these bonds compete with each other, meaning only about four can form at the same time.

The **25** ligand can form hydrogen bonds with specific amino acids in the 1KZN protein domain. These hydrogen bonds involve the following atoms and distances:

- Ligand’s keto-ester oxygen atom (acceptor) to ASN 46 A amine (donor): 2.83 Å
- Ligand’s 1,2,3-triazole nitrogen (acceptor) to ARG 76 A amine (donor): 2.43 and 2.61 Å
- Ligand’s ester oxygen atom (acceptor) to ARG 76 A amine (donor): 2.25, 2.26, and 2.45 Å

### 2.14 2H94 Protein Domain

**Figure 7:**
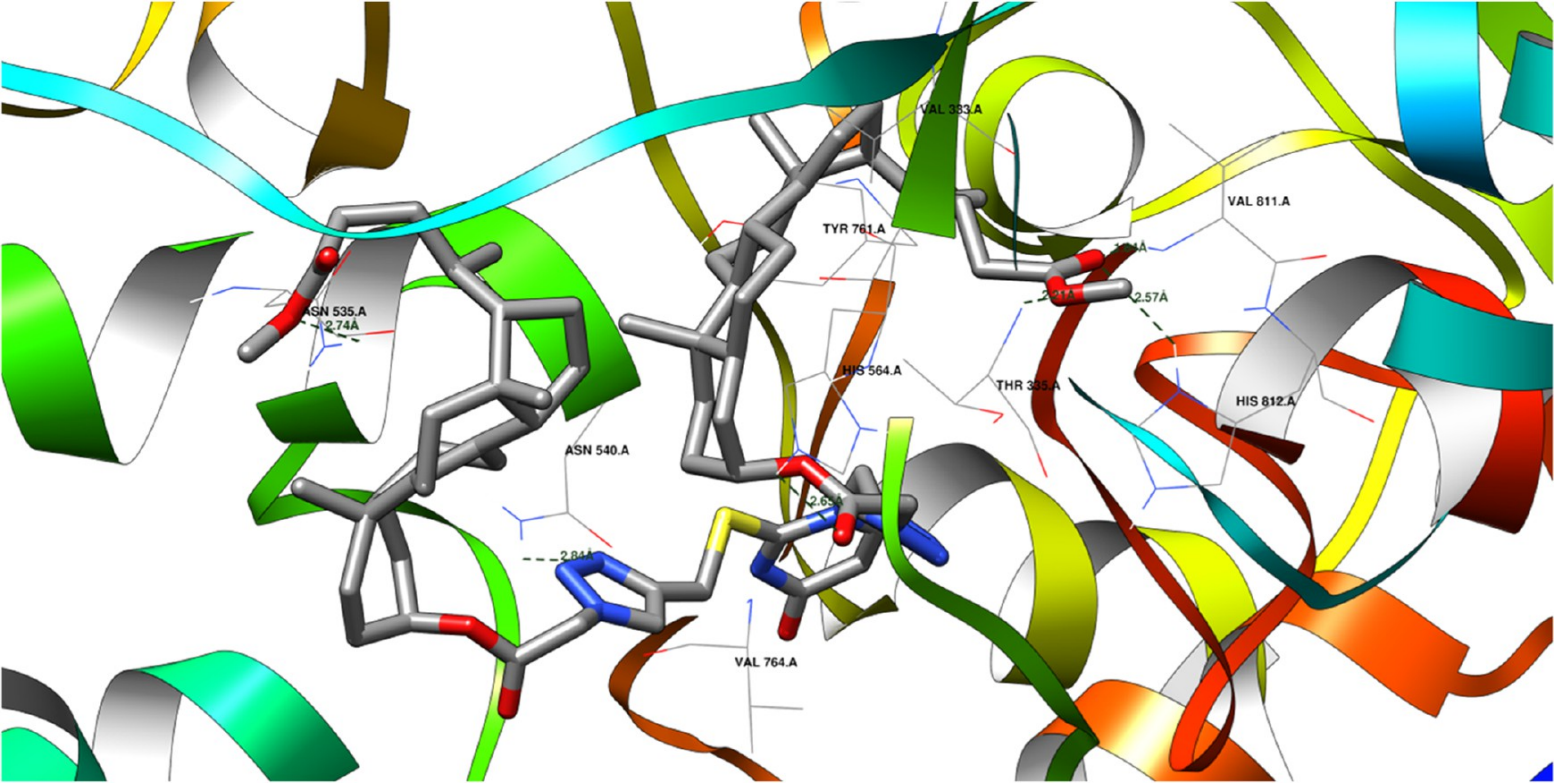
Potential hydrogen bonds between ligand **19** and the 2H94 protein domain.

For protein domain 2H94, the newly discovered ligands consistently exhibit significantly lower affinities than the native ligand. Despite this, their affinities are still noteworthy, suggesting potential anticancer activity. In this case, the RMSD for the initial pose recreation is 0.54 Å (Table 5), demonstrating faithful reproduction of the native ligand’s pose.^81^Figure 7 shows how a protein and a molecule called a **19** ligand might bond together. They could form up to six hydrogen bonds, like tiny bridges holding them together. However, some of these bonds compete with each other, meaning only about five can form at the same time.

The **19** ligand can form hydrogen bonds with specific amino acids in the 2H94 protein domain. These hydrogen bonds involve the following atoms and distances:

- Ligand’s keto-ester oxygen atom (acceptor) to ASN 535 A amine (donor): 2.74 Å
- Ligand’s 1,2,3-triazole nitrogen (acceptor) to ASN 540 A amine (donor): 2.84 Å
- Ligand’s keto-ester oxygen atom (acceptor) to HIS 564 A amine (donor): 2.65 Å
- Ligand’s keto-ester oxygen atom (acceptor) to VAL 811 A amine (donor): 1.84 Å
- Ligand’s keto-ester oxygen atom (acceptor) to THR 335 A amine (donor): 2.21 Å
- Ligand’s keto-ester oxygen atom (acceptor) to HIS 812 A amine (donor): 2.57 Å

### 2.15 2Q85 Protein Domain

**Figure 8:**
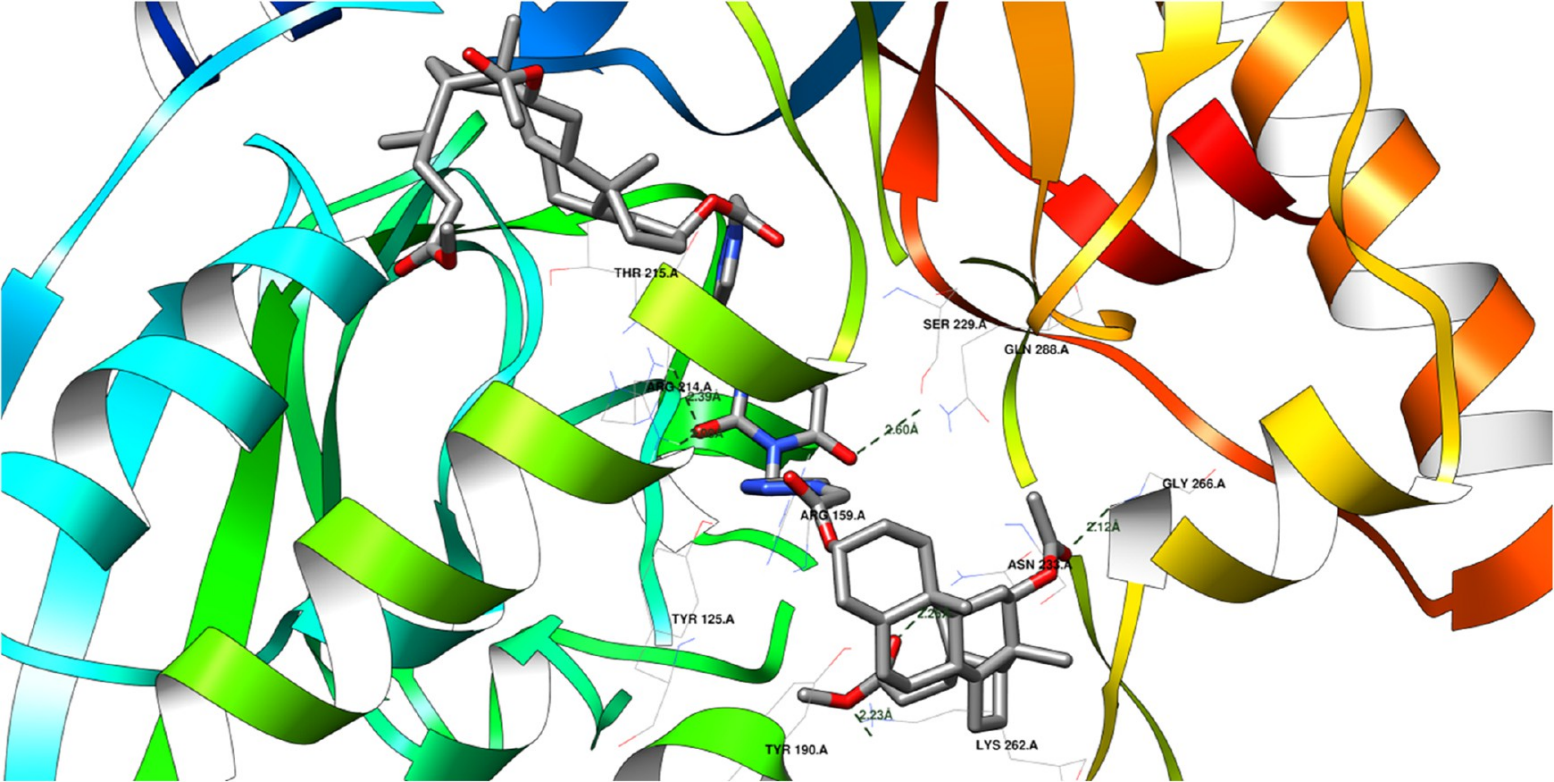
Potential hydrogen bonds between ligand **17** and the 2Q85 protein domain.

**Figure 9:**
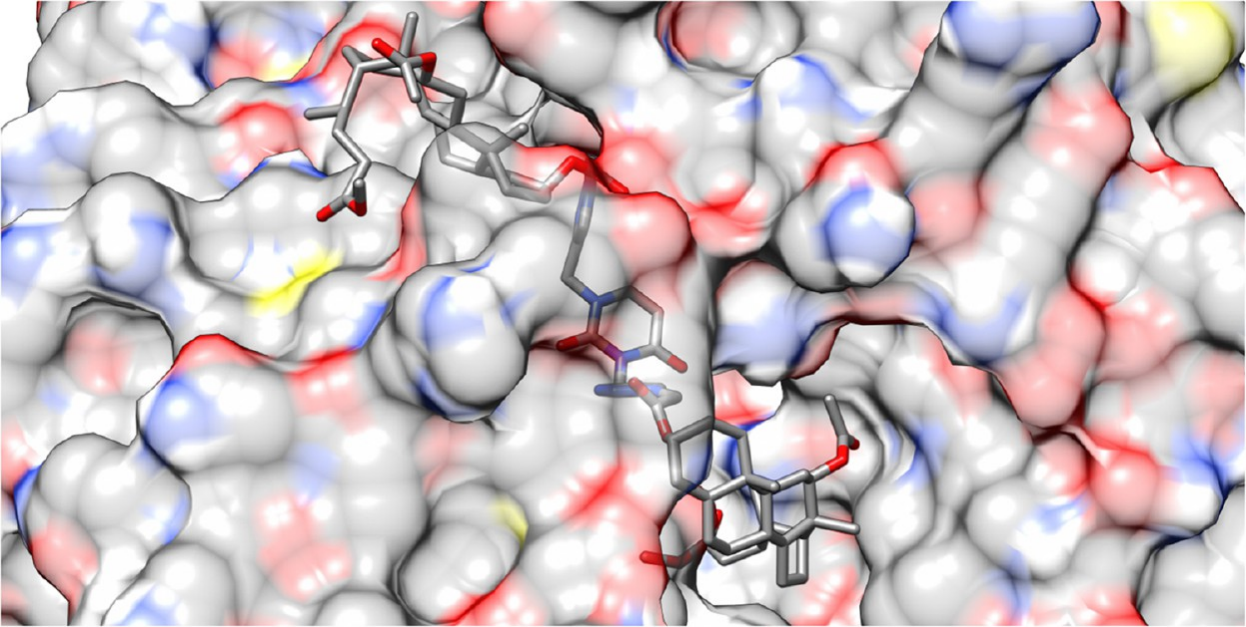
Illustration of the fitting of the **17** ligand into the binding site of the 2Q85 protein domain.

In the case of protein domain 2Q85, some new ligands exhibit affinities similar to or even better than the initial ligand. Notably, the **17** ligand shows significantly higher affinity. However, most other ligands have lower affinities than the native ligand, indicating antibacterial activity. The RMSD for this domain is 1.54 Å (Table 5), indicating reasonable pose reproduction.^81^Figure 8 shows how a protein and a molecule called a **17** ligand might bond together. They could form up to six hydrogen bonds, like tiny bridges holding them together. However, some of these bonds compete with each other, meaning only about five can form at the same time. Figure 9 shows the same ligand bound to the 2Q85 protein domain similarly. The protein is shown as a surface instead of a ribbon, but the interactions remain the same.

The **17** ligand can form hydrogen bonds with specific amino acids in the 2Q85 protein domain. These hydrogen bonds involve the following atoms and distances:

- Ligand’s keto-ester oxygen atom (acceptor) to ASN 233 A amine (donor): 2.26 Å
- Ligand’s ether oxygen atom (acceptor) to LYS 262 A amine (donor): 2.23 Å
- Ligand’s keto-ester oxygen atom (acceptor) to GLY 266 A amine (donor): 2.12 Å
- Ligand’s uracil oxygen atom (acceptor) to ARG 214 A amine (donor): 2.08 and 2.39 Å
- Ligand’s uracil oxygen atom (acceptor) to SER 229 A hydroxyl group (donor): 2.60 Å

In this case, the hydrogen bonds between the ligand’s keto-ester oxygen and ASN 233 A, ether oxygen and LYS 262 A, and keto-ester oxygen and GLY 266 A are likely the strongest because they have the shortest distances.

**Figure 10:**
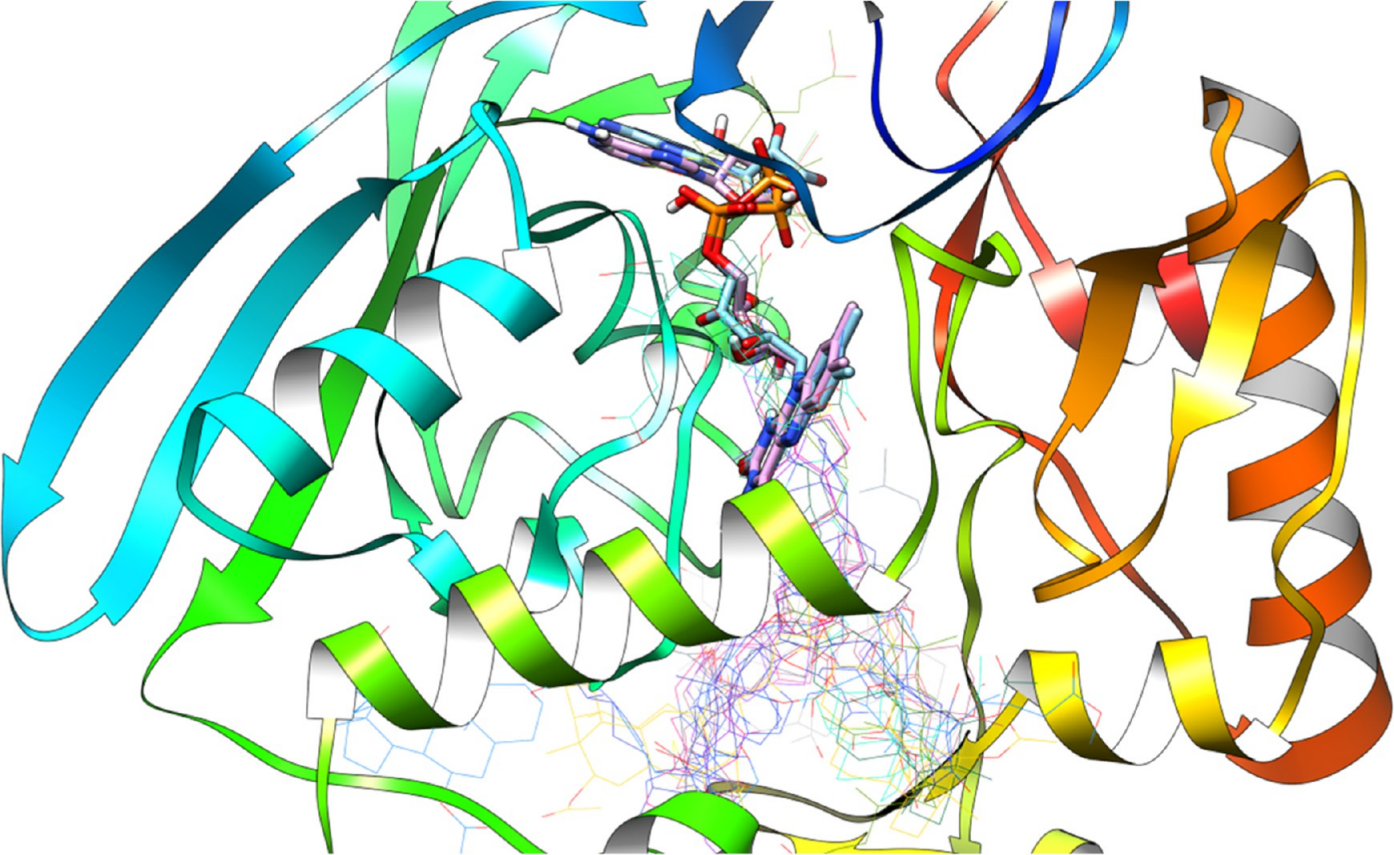
Native ligand, redocked ligand (sticks), and all new ligands at once are wired representations within the 2Q85 protein domain’s active site.

Figure 10 shows all the different ligands docked into the 2Q85 protein domain. The docked ligands are shown as wireframe models, while the native ligand (the ligand that the protein naturally binds to) and the redocked ligand (a ligand that was docked back into the binding site) are shown as solid models.

The low RMSD (Root Mean Square Deviation) value indicates that the docked ligands occupy the same binding site as the native ligand. RMSD is a measure of how closely two structures match up. A lower RMSD value means that the structures are more similar.

In this case, the low RMSD value means that the docked ligands bind to the protein, similarly to the native ligand. This suggests that the docking procedure found the ligands’ correct binding site.

### 2.16 5V5Z Protein Domain

Finally, for protein domain 5V5Z, the new ligands exhibit similar or higher affinities than the native ligand, suggesting genuine antifungal activity. Notably, the energy-favorable pose (out of the top 10 poses) has been chosen based on the lowest root-mean-square error (RMSE), which equals 1.65 Å (Table 5).^81^

**Figure 11:**
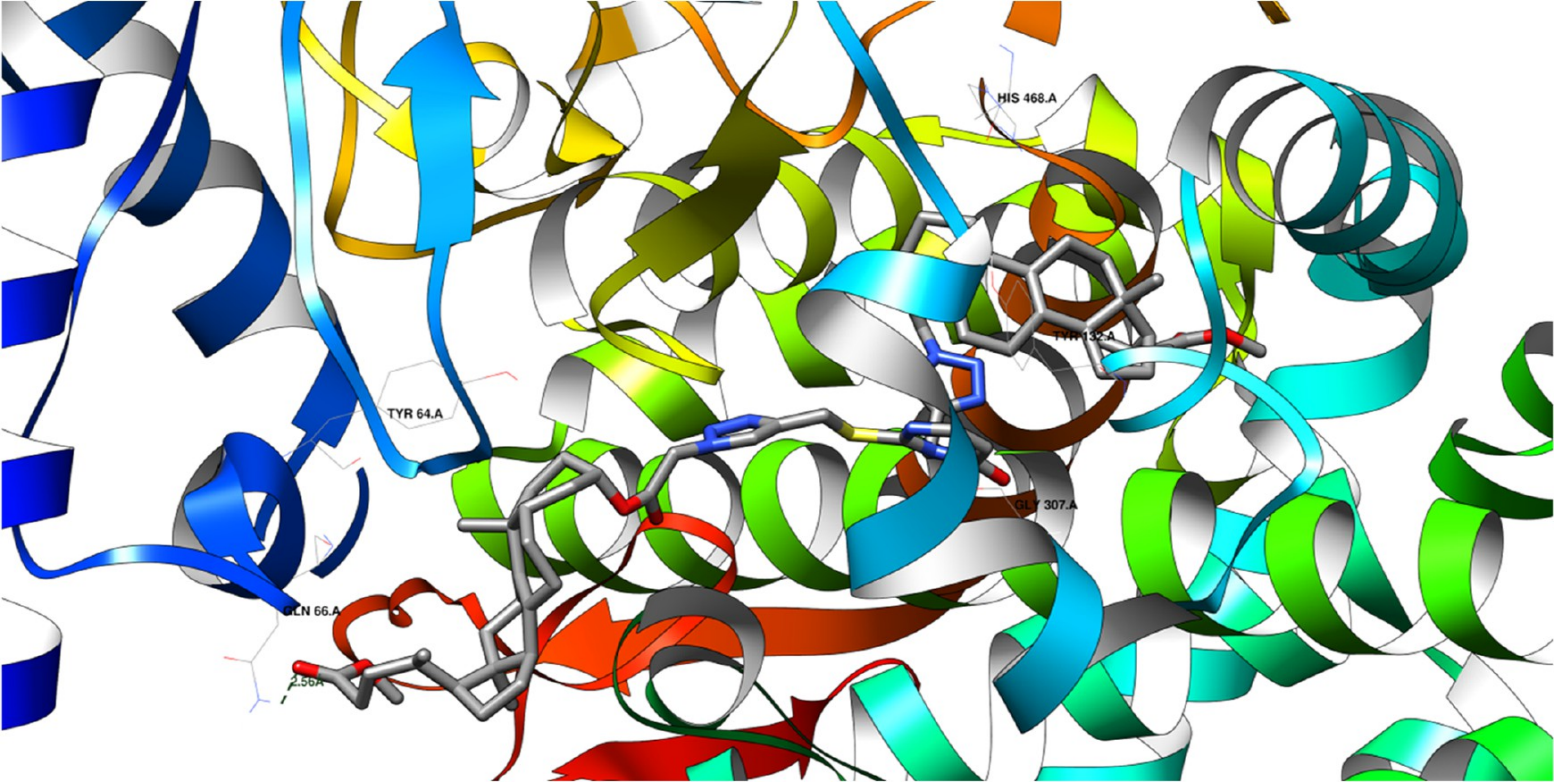
Potential hydrogen bonds between ligand **22** and the 5V5Z protein domain are shown in the figure. One hydrogen bond will likely be formed between the ligand’s keto-ester oxygen atom (an acceptor) and the GLN 66 A residue of the domain. The length of this hydrogen bond is 2.56 Å.

**Figure 12:**
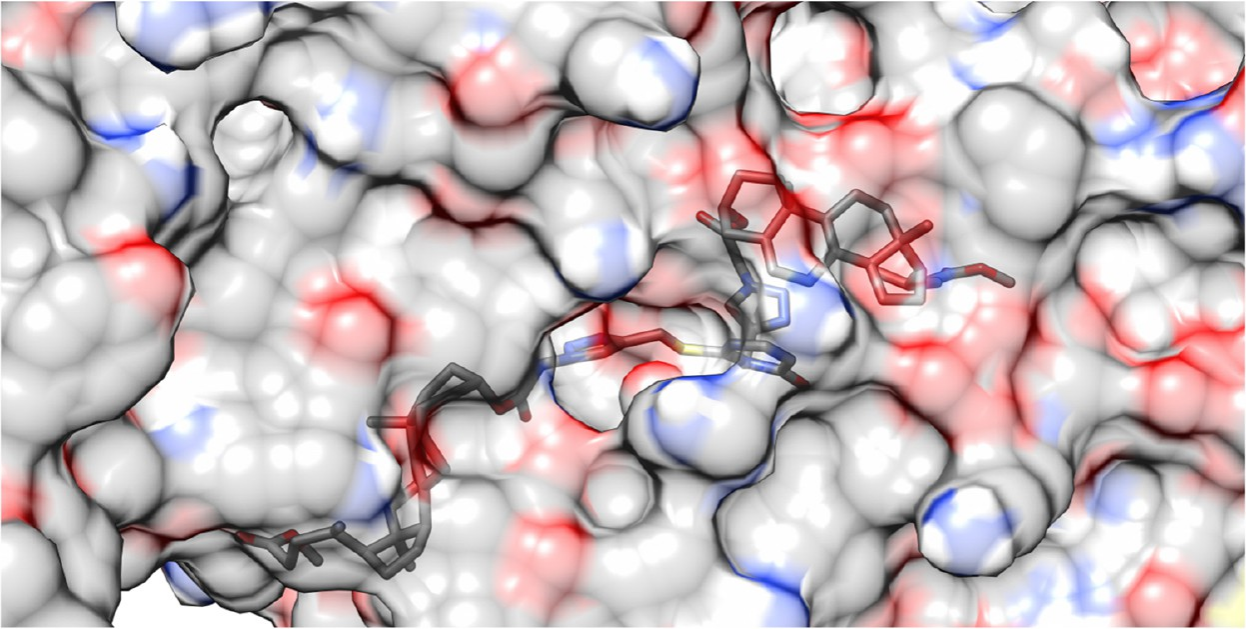
Illustration of the fitting of the **22** ligand into the binding site of the 5V5Z protein domain.

Figure 11 shows the potential hydrogen bonds between the protein domain and the **22** ligand. There is the possibility of formation of at least one hydrogen bond. Figure 12 shows the same ligand in the binding site of the 5V5Z protein domain but with the protein surface instead of the ribbon.

**Figure 13:**
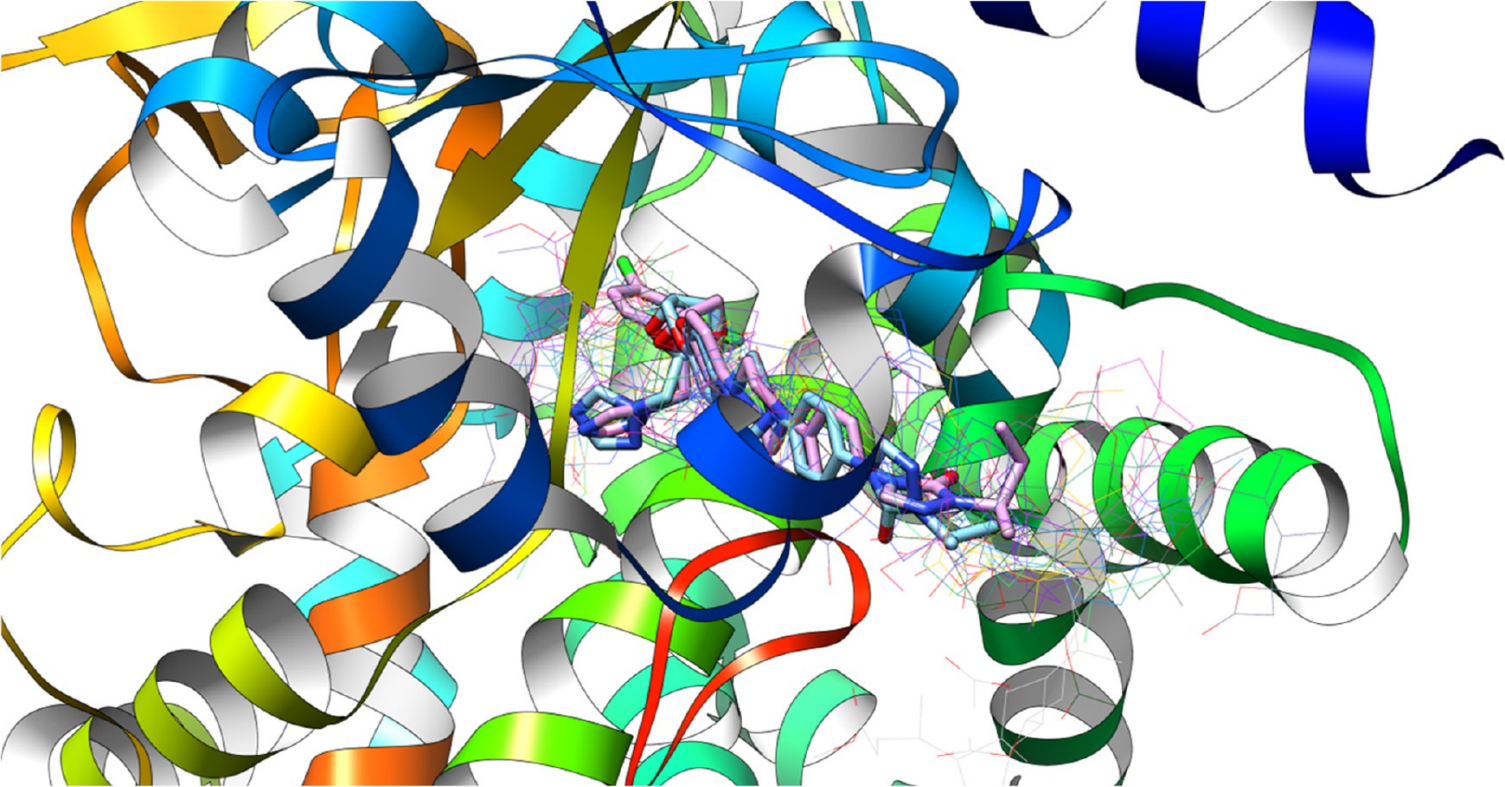
Native ligand, redocked ligand (sticks), and all new ligands at once are wired representations within the 5V5Z protein domain’s active site.

Figure 13 shows all the docked ligands (represented as wireframes) and the native and redocked ligands. The low value of RMSD (root-mean-square deviation) can be explained by the fact that all the ligands occupy the same binding site of the 5V5Z protein domain.

All the ligands occupying the same binding site suggest competing for the same binding site. This could have implications for drug design, as it could be possible to design ligands that are more selective for the binding site and, therefore, have a higher affinity for the protein.

## 3 Experimental Section

### 3.1 Synthesis

#### 3.1.1 General Procedure for the Synthesis of Compounds (**3**–**5**)

Uracil (**1**) or 2-thiouracil (**2**) (11.9 mmol) was dissolved in 5 mL of anhydrous DMF, then K_2_CO_3_ was added and left for 30 min. The mixture was cooled to 0 °C, and propargyl bromide (26.2 mmol) was added. After 24 h of stirring, the reaction was stopped. DMF was evaporated, and the crude product obtained was dissolved in 10 mL of ethyl acetate. The organic layer was washed with brine (2 × 15 mL) and dried over anhydrous Na_2_SO_4_. As an eluent, the crude compound was purified by column chromatography on silica gel using ethyl acetate/hexane (1.5:1). Two new compounds were isolated: **4** (323 mg, 26%) and **5** (443 mg, 34%).

#### 3.1.2 General Procedure for the Synthesis of Compounds (**16**–**26**)

The synthesis of azidoacetyl-substituted bile acids (**9**–**11**) or sterols (**14**–**15**) derivatives has been described previously.^46^

An azidoacetate derivative of lithocholic acid (**9**) (201 mg, 0.426 mmol) or sterol (**14**) was dissolved in a *tert-*butanol/methanol mixture (5:1, 6 mL). A propargyl derivative of uracil (**3**) or 2-thiouracil (**4**/**5**) (40 mg, 0.213 mmol) was added at 65 °C (in a water bath). CuSO_4_·5H_2_O (3 mg, 3 mol %) and sodium ascorbate (9 mg, 20 mol %) in water (0.3 mL) were then added to the dissolved mixture. The mixture was heated to 60–65 °C in a water bath for 1 h. The resulting mixture was extracted with chloroform (10 mL), washed with brine (15 mL), and dried using anhydrous Na_2_SO_4_. After evaporating the solvent and purifying the residue over silica gel (CHCl_3_/EtOAc, 20:1), a total of 162 mg (72%) of the product (**16**) and 155 mg (65%) of the product (**25**) were obtained.

##### 3.1.2.1 *N*(1),*N*(3)-Di(prop-2-yne-1-yl) Uracil (**3**)

White solid (1120 mg, 63%). ^1^H NMR (400 MHz, CDCl_3_): δ ppm 7.49 (d, *J* = 8.0 Hz, 1H, CH-5), 5.86 (d, *J* = 8.0 Hz, 1H, CH-6), 4.72 (ds, *J* = 2.5 Hz, 2H, N(3)CH_2_–7′), 4.62 (ds, *J* = 2.6 Hz, 2H, N(1)CH_2_–7), 2.52 (d, *J* = 2.6 Hz, 1H, CH-9), 2.19 (d, *J* = 2.5 Hz, 1H, CH-9′); ^13^C {^1^H} NMR (101 MHz, CDCl_3_) δ 161.6 (C-4), 150.3 (C-2), 140.7 (C-6), 102.2 (C-5), 77.8 (C-8), 75.9 (C-8′), 75.8 (C-9), 70.4 (C-9′), 37.4 (C-7), 30.4 (C-7′); FT-IR (KBr, cm^–1^) ν_max_: 3303, 2306, 1717, 1676, 1275.

##### 3.1.2.2 *N*(1)-(Prop-2-yne-1-yl)-*S*(2)-(prop-2-yn-1-yl) Thiouracil (**4**)

White solid (323 mg, 26%). ^1^H NMR (400 MHz, CDCl_3_): δ ppm 7.77 (d, *J* = 6.5 Hz, 1H, CH-6), 6.26 (d, *J* = 6.5 Hz, 1H, CH-5), 4.85 (ds, *J* = 2.5 Hz, 2H, N(3)CH_2_), 4.02 (ds, *J* = 2.7 Hz, 2H, SCH_2_), 2.32 (t, *J* = 2.5 Hz, 1H, CH-9′), 2.27 (t, *J* = 2.7 Hz, 1H, CH-9); ^13^C {^1^H} NMR (101 MHz, CDCl_3_) δ 160.8 (C-4), 160.7 (C-2), 152.0 (C-6), 110.8 (C-5), 77.7 (C-8′), 75.8 (C-8), 73.1 (C-9′), 72.0 (C-9), 33.1 (C-7), 21.1 (C-7′); FT-IR (KBr, cm^–1^) ν_max_: 3281, 3228, 3174, 2973, 2927, 2122, 1691, 1560, 1495; EI-MS (*m*/*z*, % int.): 203 (M^+^, 100), 107 (10), 78 (25).

##### 3.1.2.3 *N*(3)-(Prop-2-yne-1-yl)-*S*(2)-(prop-2-yn-1-yl) Thiouracil (**5**)

White solid (443 mg, 34%). ^1^H NMR (400 MHz, CDCl_3_): δ ppm 8.30 (d, *J* = 5.7 Hz, 1H, CH-6), 6.50 (d, *J* = 5.7 Hz, 1H, CH-5), 5.03 (ds, *J* = 2.5 Hz, 2H, N(3)CH_2_), 3.91 (ds, *J* = 2.6 Hz, 2H, SCH_2_), 2.53 (t, *J* = 2.4 Hz, 1H, CH-9′), 2.19 (t, *J* = 2.6 Hz, 1H, CH-9); ^13^C {^1^H} NMR (101 MHz, CDCl_3_) δ 169.8 (C-4), 167.5 (C-2), 157.8 (C-6), 104.1 (C-5), 79.7 (C-8′), 77.8 (C-8), 75.3 (C-9′), 70.5 (C-9), 54.1 (C-7), 19.4 (C-7′); FT-IR (KBr, cm^–1^) ν_max_: 3287, 3173, 2972, 2927, 2122, 1560, 1435, 1344, 1306, 1013; EI-MS (*m*/*z*, % int.): 204 (25), 164 (100), 69 (23).

##### 3.1.2.4 *N*(1),*N*(3)-Di[2-(methyl 5β- cholan-24-oate)-2-oxoethyl-1*H*-1,2,3-triazole-1-(3-carboxylate)] Uracil (**16**)

Oil (224 mg, 72%). ^1^H NMR (400 MHz, CDCl_3_): δ ppm 7.84 (s, 1H, CH-triazole ring), 7.74 (s, 1H, CH-triazole ring), 7.45 (d, *J* = 7.9 Hz, 1H, CH-6), 5.74 (d, *J* = 7.9 Hz, 1H, CH-5), 5.25 (s, 2H, CH_2_–27′), 5.12 (s, 2H, CH_2_–27″) 5.08 (s, 2H, N(3)–CH_2_–triazole ring), 5.03 (s, 2H, N(1)–CH_2_–triazole ring), 4.86–4.81 (m, 2H, 3′β-H and 3″β-H), 3.66 (s, 6H, OCH_3_-25′ and OCH_3_-25″), 0.93 (d, *J* = 6.4 Hz, 6H, CH_3_-21′ and CH_3_-21″), 0.92 (s, 6H, CH_3_-19′ and CH_3_-19″), 0.65 (s, 6H, CH_3_-18′ and CH_3_-18″); ^13^C {^1^H} NMR (101 MHz, CDCl_3_) δ 174.7 (C-24′/24″), 165.7 (3′α–OCO), 165.6 (3″α–OCO), 162.4 (C-4), 151.1 (C-2), 143.3 (C-6), 142.4 (C-triazole ring), 142.2 (C-triazole ring), 125.3 (CH-triazole ring), 125.0 (CH-triazole ring), 102.0 (C-5), 76.7 (C-3′/C-3″), 56.4, 56.3, 56.0, 55.9, 51.5 (C-25′/25″), 51.0 (C-27′/27″), 50.9, 44.3, 42.7, 41.8, 40.4, 40.4, 40.0, 37.8, 36.0 (N(1)–CH_2_), 35.7, 35.3, 34.8, 34.5, 32.0, 31.0, 30.9, 30.4 (N(3)–CH_2_), 28.1, 26.9, 26.4, 26.2, 24.1, 23.2 (C-19′/19″), 20.8, 18.2 (C-21′/21″), 12.0 (C-18′/18″); FT-IR (KBr, cm^–1^) ν_max_: 2941, 2867, 1741, 1667, 1451, 1217; ESI-MS (*m*/*z*): 1174 [C_64_H_94_N_8_O_10_ + K]^+^, 1171 [C_64_H_94_N_8_O_10_ + Cl]^−^, 1158 [C_64_H_94_N_8_O_10_ + Na]^+^.

##### 3.1.2.5 *N*(1),*N*(3)-Di[2-(methyl 12α-acetoxy-5β-cholan-24-oate)-2-oxoethyl-1*H*-1,2,3-triazole-1-(3-carboxylate)] Uracil (**17**)

Oil (188 mg, 63%). ^1^H NMR (400 MHz, CDCl_3_): δ ppm 7.85 (s, 1H, CH-triazole ring), 7.75 (s, 1H, CH-triazole ring), 7.46 (d, *J* = 7.9 Hz, 1H, CH-6), 5.74 (d, *J* = 7.9 Hz, 1H, CH-5), 5.24 (s, 2H, CH_2_–27′), 5.12 (s, 2H, CH_2_–27″) 5.09 (d, 2H, 12′β-H and 12″β-H), 5.08 (s, 2H, N(3)–CH_2_–triazole ring), 5.02 (s, 2H, N(1)–CH_2_–triazole ring), 4.80–4.78 (m, 2H, 3′β-H and 3″β-H), 3.67 (s, 6H, OCH_3_-25′ and OCH_3_-25″), 2.11 (s, 6H, 12′α-OAc and 12″α-OAc), 0.91 (s, 6H, CH_3_-19′ and CH_3_-19″), 0.83 (d, *J* = 6.4 Hz, 6H, CH_3_-21′ CH_3_-21″), 0.73 (s, 6H, CH_3_-18′ and CH_3_-18″); ^13^C {^1^H} NMR (101 MHz, CDCl_3_) δ 174.6 (C-24′/24″), 170.4 (12′/12″α–OCO), 165.7 (3′α–OCO),165.5 (3″α–OCO), 162.4 (C-4), 151.1 (C-2), 143.3 (C-6), 142.4 (C-triazole ring), 142.2 (C-triazole ring), 125.2 (CH-triazole ring), 125.0 (CH- triazole ring), 102.0 (C-5), 75.82 (C-12′/12″), 75.79 (C-3′/3″), 51.5 (C-25′/25″), 51.0 (C-27′/27″), 50.1, 49.4, 49.3, 47.5, 45.0, 44.2, 41.8, 36.0 (N(1)–CH_2_), 35.6, 34.7, 34.5, 34.4, 34.0, 32.0, 31.0, 30.8 (N(3)–CH_2_), 27.3, 25.8, 25.6, 23.4, 23.0 (C-19′/19″), 21.4 (C-29′/29″), 17.5 (C-21′/21″), 12.4 (C-18′/18″); FT-IR (KBr, cm^–1^) ν_max_: 2950, 2870, 1736, 1667, 1451, 1246, 1216; ESI-MS (*m*/*z*): 1289 [C_68_H_98_N_8_O_14_+K]^+^, 1286 [C_68_H_98_N_8_O_14_ + Cl]^−^, 1274 [C_68_H_98_N_8_O_14_ + Na]^+^.

##### 3.1.2.6 *N*(1),*N*(3)-Di[2-(methyl 7α,12α-diacetoxy-5β- cholan-24-oate)-2-oxoethyl-1*H*-1,2,3-triazole-1-(3-carboxylate)] Uracil (**18**)

Oil (140 mg, 78%). ^1^H NMR (400 MHz, CDCl_3_): δ ppm 7.86 (s, 1H, CH-triazole ring), 7.76 (s, 1H, CH-triazole ring), 7.46 (d, *J* = 7.9 Hz, 1H, CH-6), 5.75 (d, *J* = 7.9 Hz, 1H, CH-5), 5.23 (s, 2H, CH_2_–27′), 5.13 (ds, 2H, CH_2_–27″) 5.09 (s, 4H, 12′β-H, 12″β-H and s, 2H, N(3)–CH_2_–triazole ring), 5.01 (s, 2H, N(1)–CH_2_–triazole ring), 4.93–4.91 (m, 2H, 7′β-H and 7″β-H), 4.72–4.61 (m, 2H, 3′β-H and 3″β-H), 3.66 (s, 6H, OCH_3_-25′ and OCH_3_-25″), 2.15 (s, 6H, 7′α-OAc and 7″α-OAc), 2.11 (s, 6H, 12′α-OAc and 12″α-OAc), 0.93 (s, 6H, CH_3_-19′ and CH_3_-19″), 0.82 (d, *J* = 6.3 Hz, 6H, CH_3_-21′ and CH_3_-21″), 0.73 (s, 6H, CH_3_-18′ and CH_3_-18″); ^13^C {^1^H} NMR (101 MHz, CDCl_3_) δ 174.5 (C-24′/24″), 170.5 (12′α–OCO), 170.4 (12″α–OCO), 170.3 (7′α–OCO), 170.2 (7″α–OCO), 165.6 (3′α–OCO), 165.5 (3″α–OCO), 162.4 (C-4), 151.1 (C-2), 143.3 (C-6), 142.4 (C-triazole ring), 142.2 (C-triazole ring), 125.3 (CH-triazole ring), 125.1 (CH-triazole ring), 102.0 (C-5), 76.5 (C-12′/12″), 75.3 (C-3′/3″), 70.5 (C-7′/7″), 51.5 (C-25′/25″), 51.0 (C-27′/27″), 47.3, 45.0, 44.2, 43.3, 40.8, 37.7, 35.9 (N(1)–CH_2_), 34.6, 34.4, 34.2, 31.2, 30.8, 30.7 (N(3)–CH_2_), 28.8, 27.1, 26.7, 25.5, 22.8 (C-19′/19″), 22.4, 21.6 (C-29′/29″), 21.4 (C-31′/31″), 17.5 (C-21′/21″), 12.2 (C-18′/18″); FT- IR (KBr, cm^–1^) ν_max_: 2952, 2872, 1736, 1668, 1452, 1238; ESI-MS (*m*/*z*): 1406 [C_72_H_102_N_8_O_18_ + K]^+^, 1402 [C_72_H_102_N_8_O_18_ + Cl]^−^, 1390 [C_72_H_102_N_8_O_18_ + Na]^+^.

##### 3.1.2.7 *N*(1),*S*(2)-[2-(methyl 5β-cholan-24-oate)-2-oxoethyl-1*H*-1,2,3-triazole-1-(3-carboxylate)] Thiouracil (**19**)

Oil (76 mg, 34%). ^1^H NMR (400 MHz, CDCl_3_): δ ppm 7.81 (s, 1H, CH-triazole ring), 7.77 (d, *J* = 6.4 Hz, 1H, CH-6), 7.70 (s, 1H, CH-triazole ring), 6.21 (d, *J* = 6.5 Hz, 1H, CH-5), 5.34 (s, 2H, N(1)–CH_2_–triazole ring), 5.09 (ds, *J* = 5.2 Hz, 4H, CH_2_–27′ and CH_2_–27″), 4.84–4.76 (m, 2H, 3′β-H and 3″β-H), 4.57 (s, 2H, SCH_2_–triazole ring), 3.67 (s, 6H, OCH_3_-25′ and CH_3_-25″), 0.93 (d, *J* = 6.4 Hz, 6H, CH_3_-21′ and CH_3_-21″), 0.92 (s, 6H, CH_3_-19′ and CH_3_-19″), 0.65 (s, 6H, CH_3_-18′ and CH_3_-18″); ^13^C {^1^H} NMR (101 MHz, CDCl_3_) δ 174.7 (C-24′/24″), 165.6 (3′α–OCO), 165.5 (3″α–OCO), 162.0 (C-4), 161.6 (C-2), 151.9 (C-6), 143.8 (C-triazole ring), 141.9 (C-triazole ring), 125.3 (CH-triazole ring), 124.2 (CH-triazole ring), 110.7 (C-5), 75.0 (C-3′/3″), 69.2, 56.4, 56.4, 56.0, 51.4 (C-25′/25″), 51.1 (C-26′/26″), 42.7, 41.9 (N(1)–CH_2_), 40.5, 40.4, 40.1, 40.0, 35.8, 35.3, 34.9, 34.5, 32.1, 32.0, 31.1, 31.0, 28.1, 27.3, 26.9, 26.5, 26.6, 26.3, 24.2, 23.2 (SCH_2_), 20.8 (C-19′/19″), 18.3 (C-21′/21″), 16.5, 12.0 (C-18′/18″); FT-IR (KBr, cm^–1^) ν_max_: 2940, 2866, 1740, 1684, 1489, 1216; ESI-MS (*m*/*z*): 1189 [C_64_H_94_N_8_O_9_S + K]^+^, 1186 [C_64_H_94_N_8_O_9_S + Cl]^−^, 1174 [C_64_H_94_N_8_O_9_S + Na]^+^.

##### 3.1.2.8 *N*(1),*S*(2)-Di[2-(methyl 12α-acetoxy-5β-cholan-24-oate)-2-oxoethyl-1*H*-1,2,3-triazole-1-(3-carboxylate)] Thiouracil (**20**)

Oil (88 mg, 47%). ^1^H NMR (400 MHz, CDCl_3_): δ ppm 7.82 (s, 1H, CH-triazole ring), 7.77 (d, *J* = 6.4 Hz, 1H, CH-6), 7.70 (s, 1H, CH-triazole ring), 6.22 (d, *J* = 6.5 Hz, 1H, CH-5), 5.33 (s, 2H, N(1)–CH_2_–triazole ring), 5.10 (d, 2H, 12′β-H and 12″β-H), 5.08 (s, 4H, CH_2_–27′ and CH_2_–27″), 4.83–4.77 (m, 2H, 3′β-H and 3″β-H), 4.56 (s, 2H, SCH_2_–triazole ring), 3.67 (s, 6H, OCH_3_-25′ and CH_3_-25″), 2.11 (ds, *J* = 2.6 Hz, 6H, 12′α-OAc and 12″α-OAc) 0.91 (s, 6H, CH_3_-19′ and CH_3_-19″), 0.81 (d, *J* = 6.4 Hz, 6H, CH_3_-21′ and CH_3_-21″), 0.73 (s, 6H, CH_3_-18′ and CH_3_-18″); ^13^C {^1^H} NMR (101 MHz, CDCl_3_) δ 174.5 (C-24′/24″), 170.4 (12′α–OCO and 12″α–OCO), 165.6 (3′α–OCO), 165.5 (3″α–OCO), 162.1 (C-4), 161.7 (C-2), 151.9 (C-6), 143.7 (C-triazole ring), 142.0 (C-triazole ring), 125.4 (CH-triazole ring), 124.2 (CH-triazole ring), 110.7 (C-5),, 75.9 (C-12′/12″), 75.3 (C-3′/3″), 51.5 (C-25′/25″), 51.1 (C-27′/27″), 49.4, 47.6, 45.0, 41.8 (N(1)–CH_2_), 40.0, 35.7, 34.7, 34.6, 34.5, 34.4, 34.0, 32.1, 31.0, 30.8, 27.3, 26.8, 26.5, 25.8, 25.6, 23.4 (SCH_2_), 23.0 (C-19′/19″), 21.4 (C-29′/29″), 17.5 (C-21′/21″), 12.4 (C-18′/18″); FT-IR (KBr, cm^–1^) ν_max_: 2950, 2869, 1737, 1683, 1490, 1246, 1241; ESI-MS (*m*/*z*): 1346 [C_68_H_98_N_8_O_13_S + Br]^−^, 1306 [C_68_H_98_N_8_O_13_S + K]^+^, 1303 [C_68_H_98_N_8_O_13_S + Cl]^−^, 1290 [C_68_H_98_N_8_O_13_S + Na]^+^.

##### 3.1.2.9 *N*(1),*S*(2)-Di[2-(methyl 7α,12α-diacetoxy-5β-cholan-24-oate)-2-oxoethyl-1*H*-1,2,3-triazole-1-(3-carboxylate)] Thiouracil (**21**)

Oil (42 mg, 43%). ^1^H NMR (400 MHz, CDCl_3_): δ ppm 7.90 (s, 1H, CH-triazole ring), 7.75 (d, *J* = 6.4 Hz, 1H, CH-6), 7.80 (s, 1H, CH-triazole ring), 6.20 (d, *J* = 6.3 Hz, 1H, CH-5), 5.34 (s, 2H, N(1)–CH_2_–triazole ring), 5.11 (d, 2H, 12′β-H and 12″β-H), 5.09 (s, 4H, CH_2_–27′ and CH_2_–27″), 4.92 (s, 2H, 7′β-H and 7″β-H) 4.69–4.64 (m, 2H, 3′β-H and 3″β-H), 4.51 (s, 2H, SCH_2_–triazol ring), 3.66 (s, 6H, OCH_3_-25′ and CH_3_-25″), 2.15 (s, 6H, 7′α-OAc and 7″α-OAc), 2.11 (s, 6H, 12′α-OAc and 12″α-OAc), 0.92 (s, 6H, CH_3_-19′ and CH_3_-19″), 0.82 (d, *J* = 6.3 Hz, 6H, CH_3_-21′ and CH_3_-21″), 0.73 (s, 6H, CH_3_-18′ and CH_3_-18″); ^13^C {^1^H} NMR (101 MHz, CDCl_3_) δ 174.5 (C-24′/24″), 170.5 (12′α–OCO), 170.5 (12″α–OCO), 170.4 (7′α–OCO), 170.3 (7″α–OCO), 165.6 (3′α–OCO), 165.4 (3″α–OCO), 164.1 (C-4), 161.4 (C-2), 152.1 (C-6), 143.8 (C-triazole), 141.9 (C-triazole), 125.3 (CH-triazole), 124.2 (CH-triazole), 111.0 (C-4), 75.3 (C-12′/12″), 75.0 (C-3′/3″), 70.5 (C-7′/7″), 60.2, 59.1, 51.52 (C-25′/25″), 51.2 (C-27′/27″), 47.3, 45.0, 43.3, 40.8 (N(1)–CH_2_), 37.7, 34.6, 34.4, 34.2, 31.2, 30.9, 30.7, 29.7, 28.8, 27.1, 26.7, 25.5, 22.8 (SCH_2_), 22.5 (C-19′/19″), 21.7 (C-29′/29″), 21.4 (C-31′/31″), 17.5 (C-21′/21″), 12.2 (C-19′/19″); FT-IR (KBr, cm^–1^) ν_max_: 2965, 2875, 1741, 1675, 1485, 1241, 1232; ESI-MS (*m*/*z*): 1290 [C_68_H_98_N_8_O_13_S + Na]^+^.

##### 3.1.2.10 *N*(3),*S*(2)-[2-(methyl 5β-cholan-24-oate)-2-oxoethyl-1*H*-1,2,3-triazole-1-(3-carboxylate)] Thiouracil (**22**)

Oil (135 mg, 60%). ^1^H NMR (400 MHz, CDCl_3_): δ ppm 8.25 (d, *J* = 5.7 Hz, 1H, CH-6), 7.83 (s, 1H, CH-triazole ring), 7.70 (s, 1H, CH-triazole ring), 6.45 (d, *J* = 5.7 Hz, 1H, CH-5), 5.54 (s, 2H, N(3)–CH_2_–triazole ring), 5.13 (s, 2H, CH_2_–27′), 5.08 (s, 2H, CH_2_–27″), 4.85–4.78 (m, 2H, 3′β-H and 3″β-H), 4.52 (s, 2H, SCH_2_–triazole ring), 3.67 (s, 6H, OCH_3_-25′ and CH_3_-25″), 0.93 (s, 6H, CH_3_-19′ and CH_3_-19″), 0.91 (d, *J* = 6.4 Hz, 6H, CH_3_-21′ and CH_3_-21″), 0.65 (s, 6H, CH_3_-18′ and CH_3_-18″); ^13^C {^1^H} NMR (101 MHz, CDCl_3_) δ 174.7 (C-24′/24″), 170.4 (C-4), 168.1 (C-2), 165.7 (3′α–OCO), 165.6 (3″α–OCO), 157.6 (C-6), 145.7 (C-triazole ring), 143.1 (C-triazole ring), 125.2 (CH-triazole ring), 123.7 (CH-triazole ring), 104.5 (C-5), 76.7 (C-3′/3″), 59.4, 56.4, 56.0, 51.4 (C-25′/25″), 51.1 (C-27′/27″), 42.7, 41.9 (N(3)–CH_2_), 40.5, 40.1, 35.8, 35.3, 34.9, 34.6, 32.1, 31.1, 31.0, 28.1, 27.0, 26.5, 26.3, 25.7, 24.2, 23.2 (SCH_2_), 20.8 (C-19′/19″), 18.3 (C-21′/21″), 12.0 (C-18′/18″); FT-IR (KBr, cm^–1^) ν_max_: 2935, 2852, 1741, 1666, 1472, 1211; ESI-MS (*m*/*z*): 1188 [C_64_H_94_N_8_O_9_S + K]^+^, 1186 [C_64_H_94_N_8_O_9_S + Cl]^−^, 1174 [C_64_H_94_N_8_O_9_S + Na]^+^.

##### 3.1.2.11 *N*(3),*S*(2)-Di[2-(methyl 12α-acetoxy-5β-cholan-24-oate)-2-oxoethyl-1*H*-1,2,3-triazole-1-(3-carboxylate)] Thiouracil (**23**)

Oil (169 mg, 78%). ^1^H NMR (400 MHz, CDCl_3_): δ ppm 8.25 (d, *J* = 5.7 Hz, 1H, CH-6), 7.83 (s, 1H, CH-triazole ring), 7.71 (s, 1H, CH-triazole ring), 6.45 (d, *J* = 5.7 Hz, 1H, CH-5), 5.53 (s, 2H, N(3)–CH_2_–triazole ring), 5.14 (s, 2H, CH_2_–27′), 5.09 (s, 4H, 12′β-H, 12″β-H and CH_2_–27″), 4.83–4.76 (m, 2H, 3′β-H and 3″β-H), 4.51 (s, 2H, SCH_2_–triazole ring), 3.67 (s, 6H, OCH_3_-25′ and CH_3_-25″), 2.11 (6H, 12′α-OAc and 12″α-OAc), 0.91 (s, 6H, CH_3_-19′ and CH_3_-19″), 0.81 (d, *J* = 6.2 Hz, 6H, CH_3_-21′ and CH_3_-21″), 0.72 (s, 6H, CH_3_-18′ and CH_3_-18″); ^13^C {^1^H} NMR (101 MHz, CDCl_3_) δ 174.6 (C-24′/24″), 170.4 (12′α–OCO, 12″α–OCO and C-4), 168.1 (C-2), 165.7 (3′α–OCO), 165.6 (3″α–OCO), 157.6 (C-6), 145.7 (C-triazole ring), 143.2 (C-triazole ring), 125.2 (CH-triazole ring), 123.7 (CH-triazole ring), 104.5 (C-5), 75.9 (C-12′/12″), 75.8 (C-3′/3″), 59.4, 51.5 (C-25′/25″), 51.1 (C-27′/27″), 49.4, 47.6, 45.0, 41.8 (N(3)–CH_2_), 35.6, 34.7, 34.6, 34.4, 34.0, 32.1, 31.0, 30.8, 27.3, 26.8, 26.5, 25.8, 25.7, 25.6, 23.5 (SCH_2_), 23.0 (C-19′/19″), 21.4 (C-29′/29″), 17.5 (C-21′/21″), 12.4 (C-18′/18″); FT-IR (KBr, cm^–1^) ν_max_: 2951, 2872, 1735, 1563, 1377, 1248; ESI-MS (*m*/*z*): 1406 [C_72_H_102_N_8_O_17_S + Na]^+^.

##### 3.1.2.12 *N*(3),*S*(2)-Di[2-(methyl 7α,12α-diacetoxy-5β-cholan-24-oate)-2-oxoethyl-1*H*-1,2,3-triazole-1-(3-carboxylate)] Thiouracil (**24**)

Oil (124 mg, 67%). ^1^H NMR (400 MHz, CDCl_3_): δ ppm 8.26 (d, *J* = 5.8 Hz, 1H, CH-6), 7.83 (s, 1H, CH-triazole ring), 7.71 (s, 1H, CH-triazole ring), 6.45 (d, *J* = 5.7 Hz, 1H, CH-5), 5.52 (s, 2H, N(3)–CH_2_–triazole ring), 5.15 (s, 2H, CH_2_–27′), 5.10 (s, 2H, 12′β-H and 12″β-H), 5.08 (s, 2H, CH_2_–27″), 4.91 (s, 2H, 7′β-H and 7″β-H), 4.72–4.62 (m, 2H, 3′β-H and 3″β-H), 4.50 (s, 2H, SCH_2_–triazole ring), 3.66 (s, 6H, OCH_3_-25′ and CH_3_-25″), 2.15 (s, 6H, 7′α-OAc and 7″α-OAc), 2.09 (s, 6H, 12′α-OAc and 12″α-OAc), 0.92 (s, 6H, CH_3_-19′ and CH_3_-19″), 0.82 (d, *J* = 6.3 Hz, 6H, CH_3_-21′ and CH_3_-21″), 0.73 (s, 6H, CH_3_-18′ and CH_3_-18″); ^13^C {^1^H} NMR (101 MHz, CDCl_3_) δ 174.5 (C-24′/24″), 170.5 (12′α–OCO and 12″α–OCO), 170.3 (7′α–OCO, 7″α–OCO and C-4), 168.1 (C-2), 165.7 (3′α–OCO), 165.6 (3″α–OCO), 157.6 (C-6), 145.6 (C-triazole ring), 143.2 (C-triazole ring), 125.2 (CH-triazole ring), 123.7 (CH-triazole ring), 104.5 (C-5), 75.3 (C-12′/12″), 75.0 (C-3′/3″), 70.5 (C-7′/7″), 60.4, 59.4, 51.5 (C-25′/25″), 51.1 (C-27′/27″), 47.3, 45., 43.3, 40.8 (N(3)–CH_2_), 37.7, 34.6, 34.4, 34.2, 31.2, 30.9, 30.7, 28.8, 27.2, 26.7, 25.6, 25.5, 22.8 (SCH_2_), 22.5 (C-19′/19″), 21.6 (C-31′/31″), 21.4 (C-29′/29″), 21.0, 19.1, 17.5 (C-21′/21″), 16.5, 14.2, 13.7, 12.2 (C-18′/18″); FT-IR (KBr, cm^–1^) ν_max_: 2949, 2869, 1736, 1563, 1439, 1246, 1214; ESI-MS (*m*/*z*): 1290 [C_68_H_98_N_8_O_13_S + Na]^+^.

##### 3.1.2.13 *N*(1),*N*(3)-Di[2-(methyl cholest-5-ene)-2-oxoethyl-1*H*-1,2,3-triazole-1-(3-carboxylate)] Uracil (**25**)

Oil (115 mg, 65%). ^1^H NMR (400 MHz, CDCl_3_): δ ppm 7.84 (s, 1H, CH-triazole ring), 7.74 (s, 1H, CH-triazole ring), 7.45 (d, *J* = 7.9 Hz, 1H, CH-6), 5.75 (d, *J* = 7.8 Hz, 1H, CH-5), 5.38 (bs, 2H, CH-6′ and CH-6″), 5.25 (s, 2H, CH_2_–29′), 5.12 (s, 2H, CH_2_–29″), 5.08 (s, 2H, N(3)–CH_2_–triazole ring), 5.02 (s, 2H, N(1)–CH_2_–triazole ring), 4.69–4.64 (m, 2H, 3′α-H and 3″α-H), 1.04 (s, 6H, CH_3_-19′ and CH_3_-19″), 0.91 (d, *J* = 6.4 Hz, 6H, CH_3_-21′ and CH_3_-21″), 0.86 (dd, *J*_1_ = 6.6 Hz, *J*_2_ = 1.8 Hz, 12H, CH_3_-26′/26″ and CH_3_-27′/27″), 0.68 (s, 6H, CH_3_-18′ and CH_3_-18″); ^13^C {^1^H} NMR (101 MHz, CDCl_3_) δ 165.6 (3′β–OCO), 165.5 (3″β–OCO), 162.4 (C-4), 151.1 (C-2), 142.4 (C-6 and C-5′/5″), 138.9 (C-triazole ring), 138.8 (C-triazole ring), 123.4 (CH-triazole ring), 123.3 (CH-triazole ring and C-6′/6″), 102.0 (C-5), 76.4 (C-3′/3″), 56.6, 56.1, 51.0 (C-29′/29″), 49.9, 44.3, 42.3, 39.7, 39.5, 37.8, 36.8, 36.5 (N(1)–CH_2_), 36.1, 36.0, 35.8, 31.8, 31.8, 29.7, 28.2, 28.0 (N(3)–CH_2_), 27.6, 24.2, 23.8, 22.8, 22.5 (C-19′/19″), 21.0 (C-26′/26″ and C-27′/27″), 19.2, 18.7 (C-21′/21″), 14.2, 11.8 (C-18′/18″); FT-IR (KBr, cm^–1^) ν_max_: 3447, 2947, 1748, 1706, 1661, 1222; ESI-MS (*m*/*z*): 1151 [C_68_H_102_N_8_O_6_ + Na]^+^.

##### 3.1.2.14 *N*(1),*N*(3)-Di[2-(methyl 5β-cholestan)-2-oxoethyl-1*H*-1,2,3-triazole-1-(3-carboxylate)] Uracil (**26**)

Oil (75%). ^1^H NMR (400 MHz, CDCl_3_): δ ppm 7.84 (s, 1H, CH-triazole ring), 7.73 (s, 1H, CH-triazole ring), 7.45 (d, *J* = 7.9 Hz, 1H, CH-6), 5.74 (d, *J* = 7.9 Hz, 1H, CH-5), 5.25 (s, 2H, CH_2_–29′), 5.11 (s, 2H, CH_2_–29″) 5.07 (s, 2H, N(3)–CH_2_–triazole ring), 5.02 (s, 2H, N(1)–CH_2_–triazole ring), 4.83–4.72 (m, 2H, 3′α-H and 3″α-H), 0.90 (d, *J* = 6.4 Hz, 6H, CH_3_-21′ and CH_3_-21″), 0.86 (dd, *J*_1_ = 6.4 Hz, *J*_2_ = 2.4 Hz, 12H, CH_3_-26′/26″ and CH_3_-27′/27″), 0.82 (s, 6H, CH_3_-19′ and CH_3_-19″), 0.65 (s, 6H, CH_3_-18′ and CH_3_-18″); ^13^C {^1^H} NMR (101 MHz, CDCl_3_) δ 165.7 (3′β–OCO), 165.6 (3″β–OCO), 162.4 (C-4), 151.1 (C-2), 143.3 (C-6), 142.4 (C-triazole ring), 142.2 (C-triazole ring), 125.3 (CH-triazole ring), 125.1 (CH-triazole ring), 102.0 (C-5), 76.5 (C-3″), 76.3 (C-3′), 56.3, 56.2, 56.2, 54.1, 51.1 (C-29′), 50.9, 44.6, 44.3, 42.5, 39.9, 39.5, 36.5 (N(1)–CH_2_), 36.1, 35.9, 35.8, 35.4, 33.8, 31.9, 28.5, 28.20, 28.0 (N(3)–CH_2_), 27.3, 24.1, 23.8, 22.8, 22.5 (C-19′/19″), 21.2 (C-26′/26″ and C-27′/27″), 18.6 (C-21′/21″), 12.2 (C-18″), 12.0 (C-18′); FT-IR (KBr, cm^–1^) ν_max_: 3440, 2935, 1740, 1620, 1215; ESI-MS (*m*/*z*): 1171 [C_68_H_106_N_8_O_6_ + K]^+^.

## 4 Conclusions

An effective synthesis, comprehensive spectroscopic characterization, and theoretical studies of the biological properties of 11 new steroid-pyrimidine bioconjugates connected with 1,2,3-triazole rings were carried out. The synthesis involved the strategic incorporation of triazole rings that linked steroid and pyrimidine units, which could offer synergistic effects in biological activity. Detailed spectroscopic and spectrometric analyses provided detailed structural information, confirming the successful formation of the bioconjugates. Additionally, the potential biological activities of the synthesized compounds were investigated in theoretical studies, such as molecular docking simulations. Computational methods provided insight into the molecular interactions between bioconjugates and target biological receptors. The combined experimental and theoretical approach confirmed the structural integrity of the synthesized compounds. It also provided valuable information about their potential biological activities, opening a promising avenue for further pharmaceutical development. Molecular docking studies predicted that the new ligands may have potential antibacterial and antifungal activities. Affinity energy for selected ligands is lower than the affinity energy of the native ligand toward *E. coli* MurB and against *C. albicans* fungi. This is a promising path for the development of new drugs, especially in the fight against infections resistant to currently available therapies. However, further experimental studies are required to confirm these predicted activities. In particular, *in vitro* and *in vivo* studies can provide valuable information on their efficacy and safety profile, enabling more informed decisions regarding drug development.

## Data Availability

The study’s data are available in the published article and its Supporting Information.

## Supporting Information Available

General information and copies of ^1^H and ^13^C NMR, ESI-MS, EI-MS, and FT-IR spectra (PDF)

## Notes

The obtained compounds were obtained under laboratory conditions. The compounds have not been subjected to cytotoxicity tests. Therefore, their effect on human or animal cells is not known.
