# Supplementary material for: Exploring Triazole-Connected Steroid-Pyrimidine Hybrids: Synthesis, Spectroscopic Characterization, and Biological Assessment
Source: ACS Omega. 2024 Aug 29;9(36):37995–8014. doi: 10.1021/acsomega.4c04800 (PMC11391466; doi:10.1021/acsomega.4c04800)
Supplement: Supplementary file 1 — ao4c04800_si_001.pdf [file ao4c04800_si_001.pdf]

***Supporting Information***

**Exploring Triazole-Connected Steroid-Pyrimidine Hybrids: Synthesis, Spectroscopic Characterization, and Biological Assessment**

Anna Kawka[a*], Damian Nowak[b], Hanna Koenig[a] Tomasz Pospieszny[a]

[a]*Department of Bioactive Products, Faculty of Chemistry, Adam Mickiewicz University, Uniwersytetu Poznańskiego 8 Street, 61-614 Poznań, Poland*

[b]*Department of Quantum Chemistry, Faculty of Chemistry, Adam Mickiewicz University, Uniwersytetu Poznańskiego 8 Street, 61-614 Poznań, Poland*

*Corresponding Author: anna.kawka@amu.edu.pl

**Table of Contents**

1. General Information S2
2. Copies of NMR spectra S3
   2.1. Copies of $^{1}$H NMR spectra S3
   2.2. Copies of $^{13}$C NMR spectra S10
3. Copies of MS spectra S17
   3.1. Copies of ESI-MS spectra S17
   3.2. Copies of EI-MS spectra S19
4. Copies of FT-IR spectra S20

## 1. General information

The following reagents were acquired from Sigma-Aldrich Corporation for the synthesis: lithocholic acid, deoxycholic acid, cholic acid, uracil, 2-thiouracil, potassium carbonate anhydrous, acetic anhydride, propargyl bromide, sodium azide, and sodium ascorbate. Chloroform, dichloromethane, dimethylformamide anhydrous, ethyl acetate, *t*-butanol, and methanol solvents were obtained from standard commercial sources such as Merck and Fisher and were used without purification. The characterization methods used are as follows:

IR Spectra: FT/IR-4600 type A instrument in either solid state or oil phase, with wavenumbers reported in $cm^{-1}$.

$^{1}H$ and $^{13}C$ NMR spectra: Varian Mercury 300 MHz spectrometer (Oxford, UK) operating at 300.07 MHz and 75.4614 MHz for $^{1}H$ and $^{13}C$, respectively. Chemical shifts are reported in ppm relative to $Me_4Si$ used as the internal standard and coupling constants (J values) in Hz. Typical conditions for $^{1}H$ spectra include a pulse width of 32°, acquisition time of 5 seconds, FT size of 32 K, digital resolution of 0.3 Hz per point, and scans ranging from 1200 to 10,000 per spectrum. For $^{13}C$ spectra, typical conditions include a pulse width of 60°, FT size of 60 K, digital resolution of 0.6 Hz per point, and the number of scans varied accordingly.

ESI-MS: Waters/Micromass (Manchester, UK) ZQ mass spectrometer equipped with a Harvard Apparatus (Saint Laurent, Canada) syringe pump, with mass-to-charge ratio (m/z) reported. Sample solutions were prepared in MeOH at a concentration of approximately $10^{-5}$ M. Standard ESI-MS mass spectra were recorded at a cone voltage of 90 V.

EI-MS: Spectrometer Bruker 320MS/420GC. Sample solutions were prepared in MeOH at a concentration of approximately $10^{-5}$ M. Standard EI-MS mass spectra were recorded at a cone voltage of 90 V.

## 2. Copies of NMR spectra

### 2.1. Copies of $^{1}$H NMR spectra

3

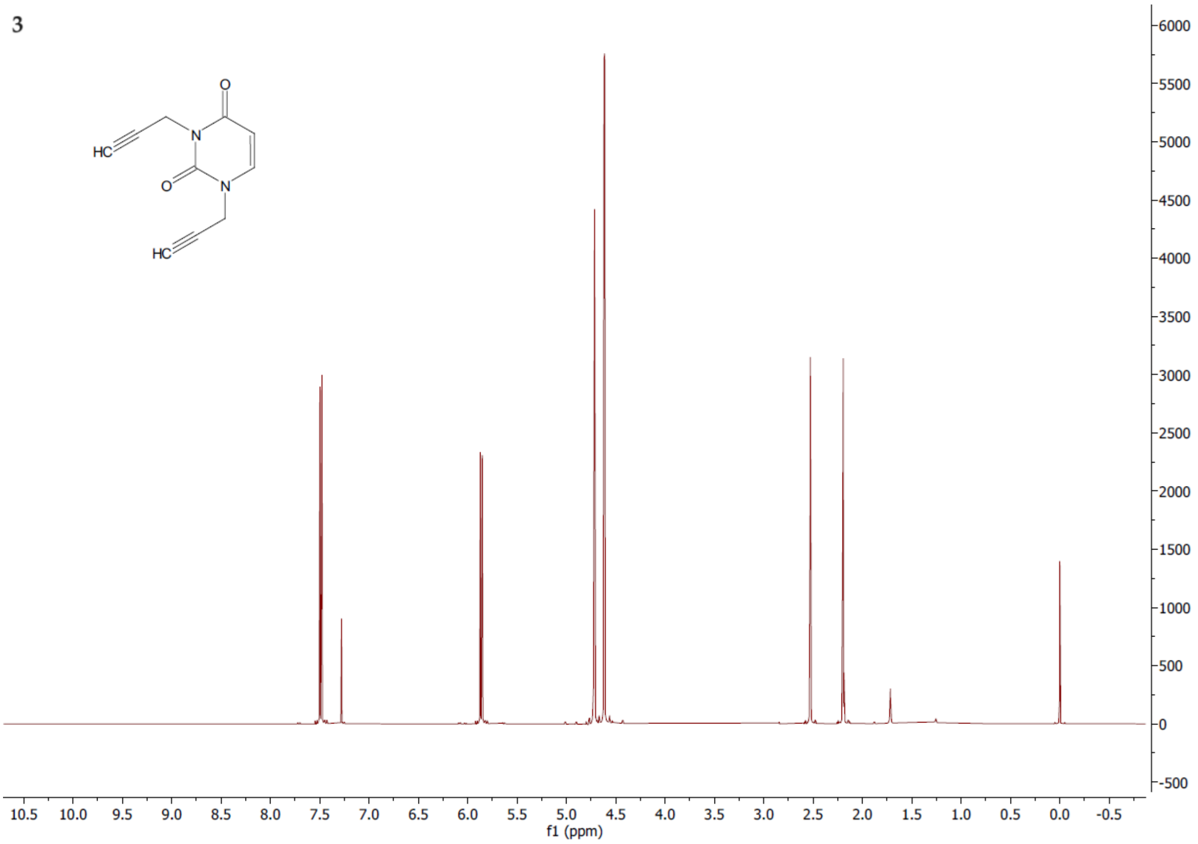

4

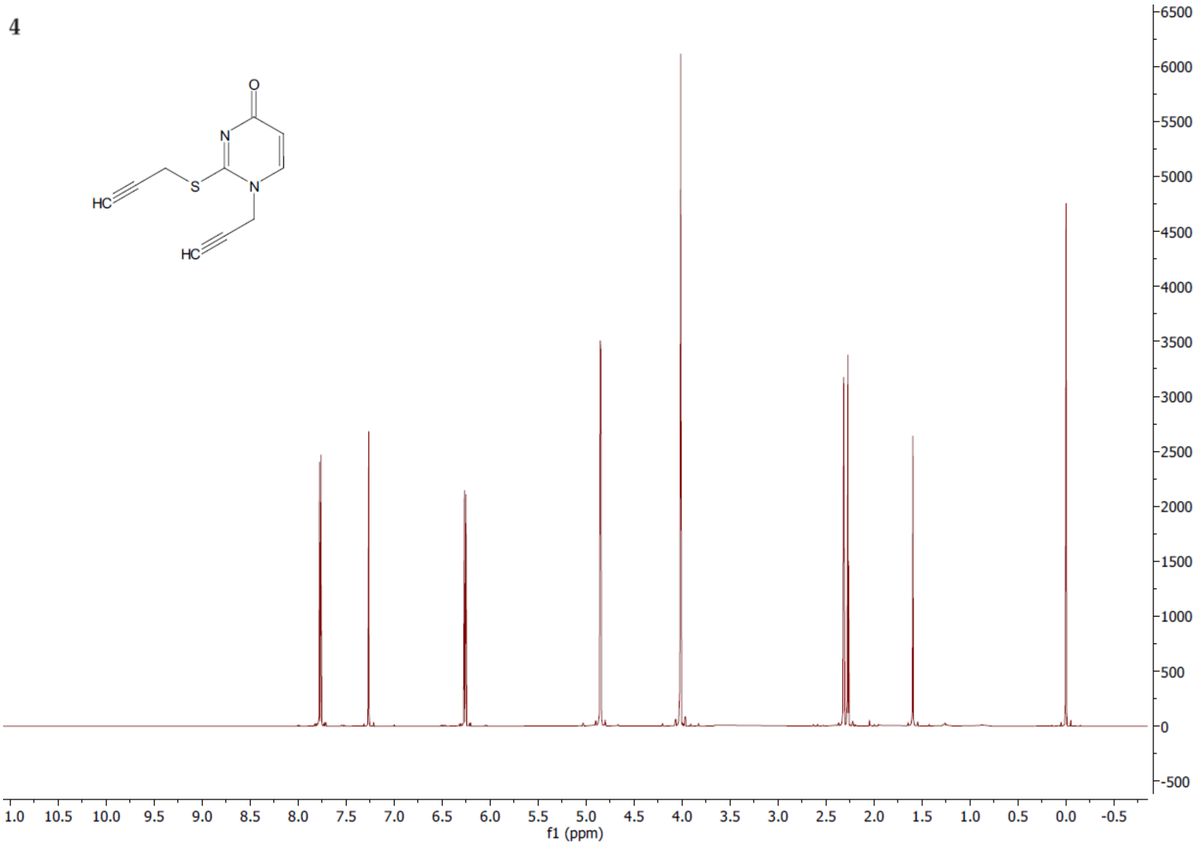

**5**

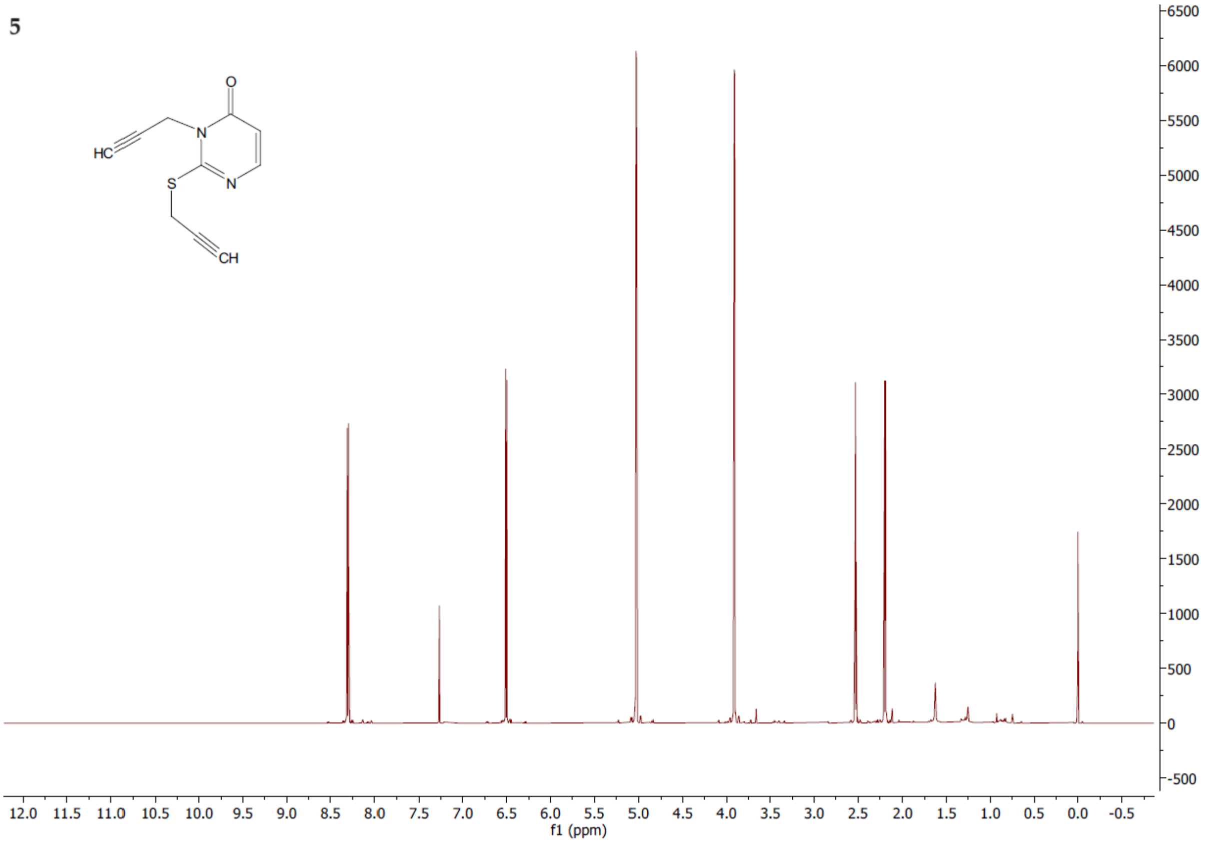


**16**

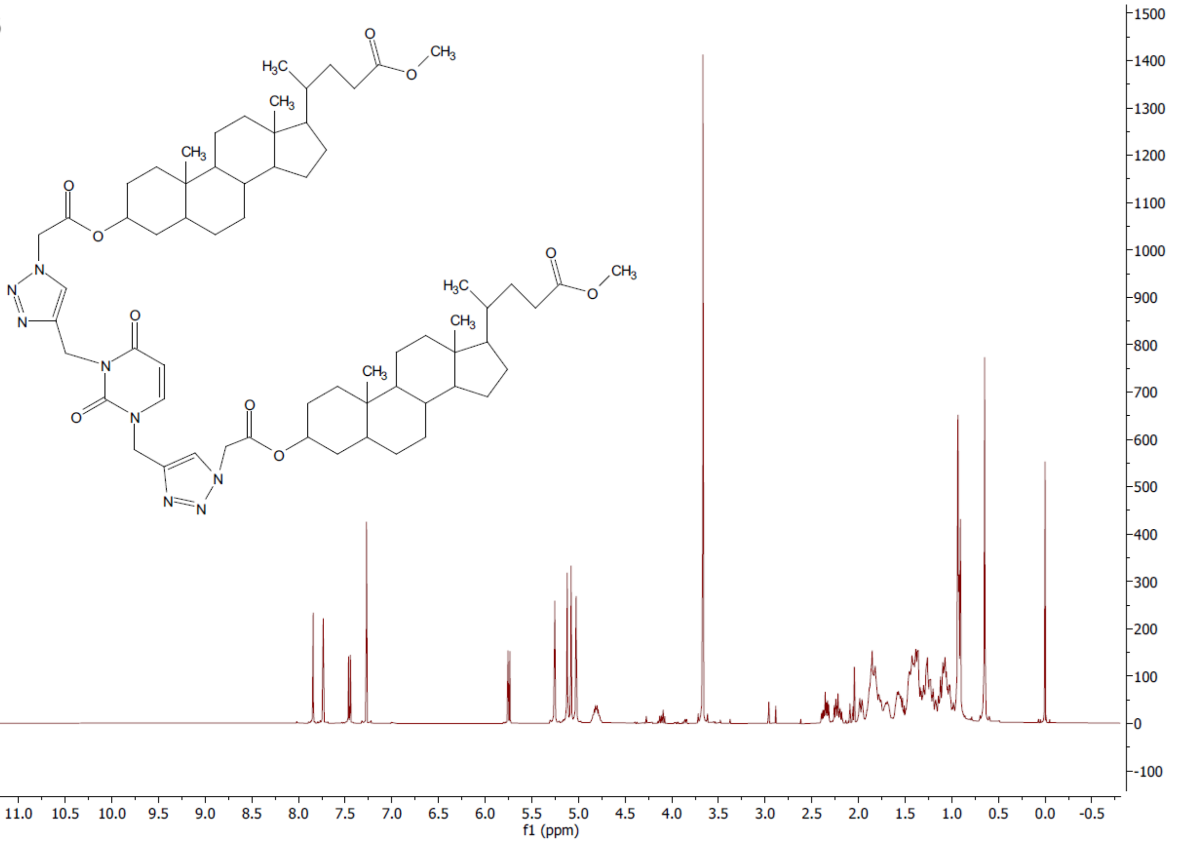

**17**

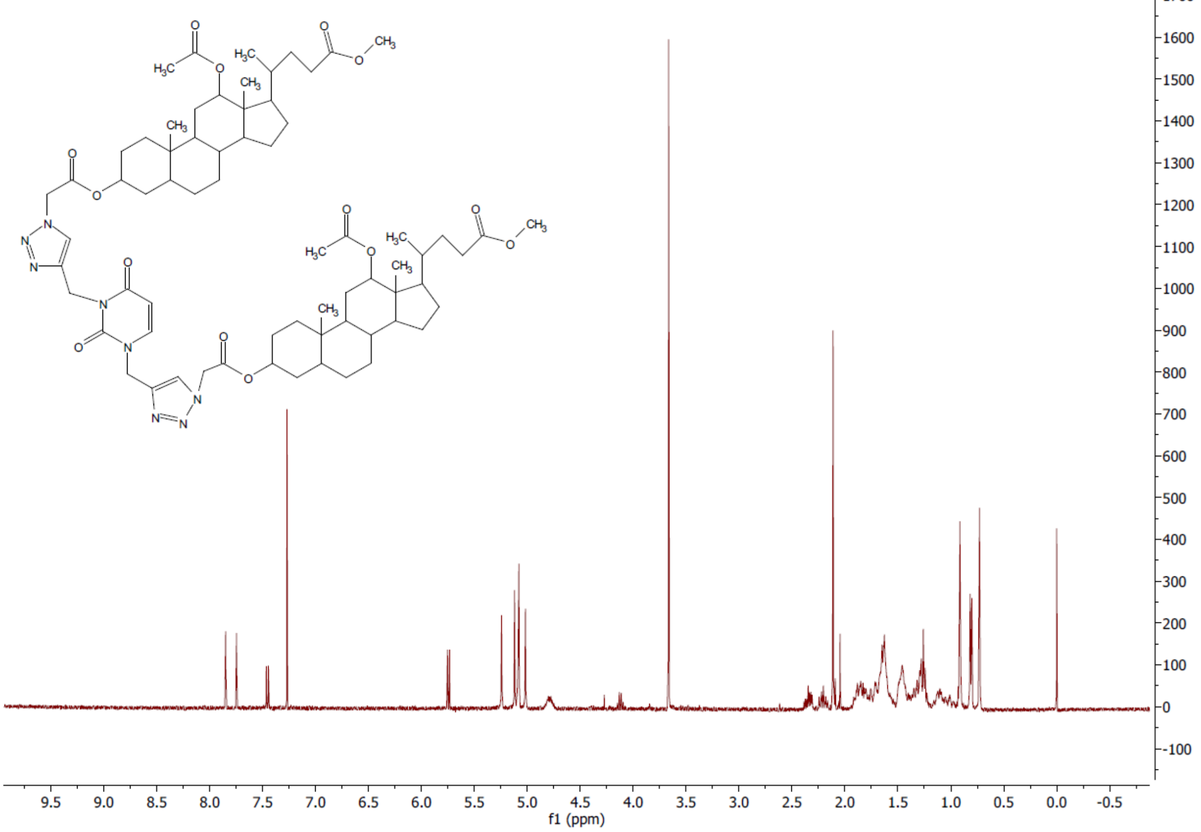


**18**

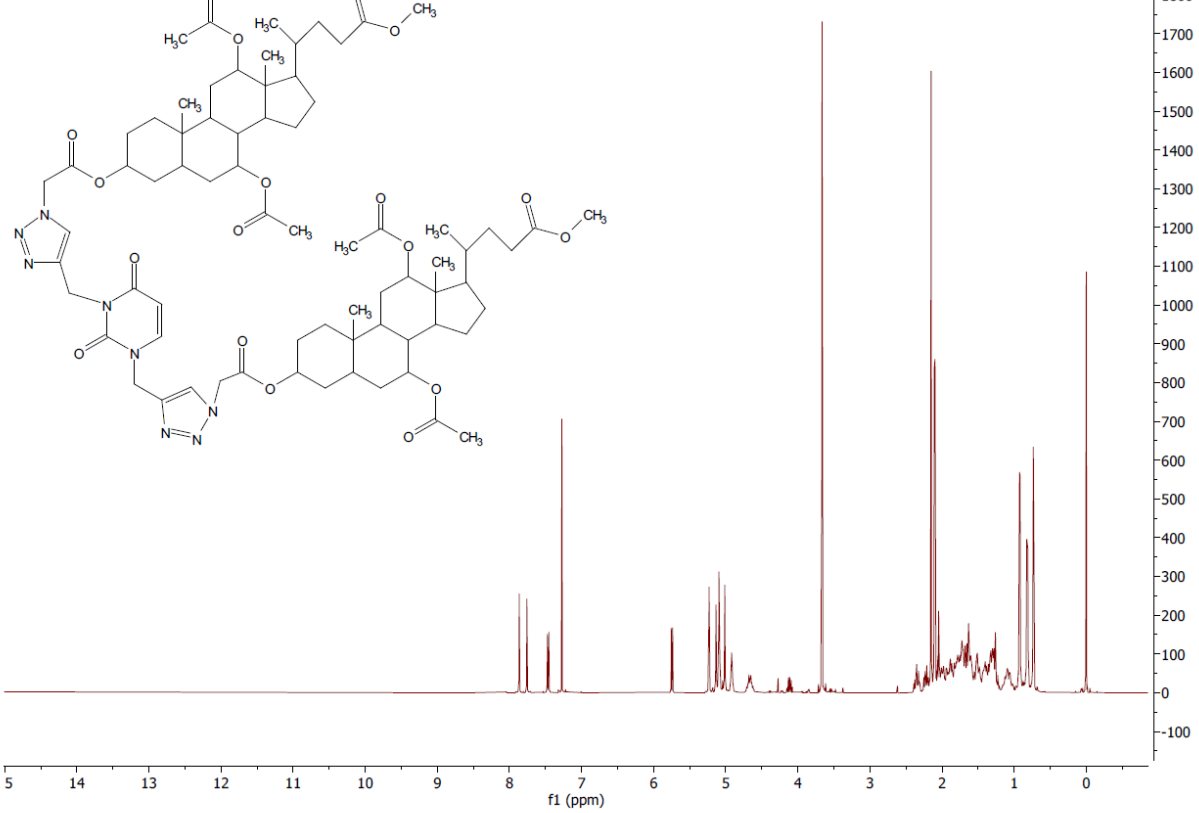

**19**

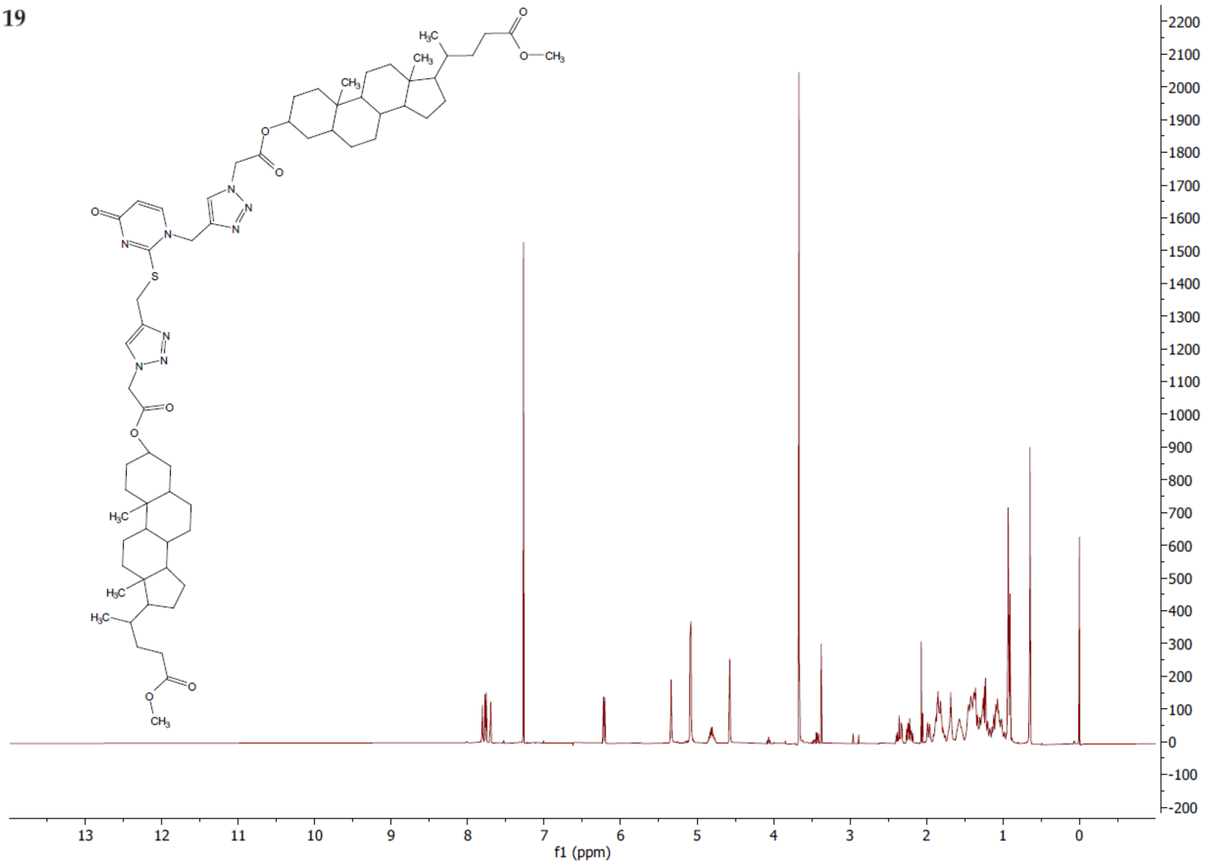


**20**

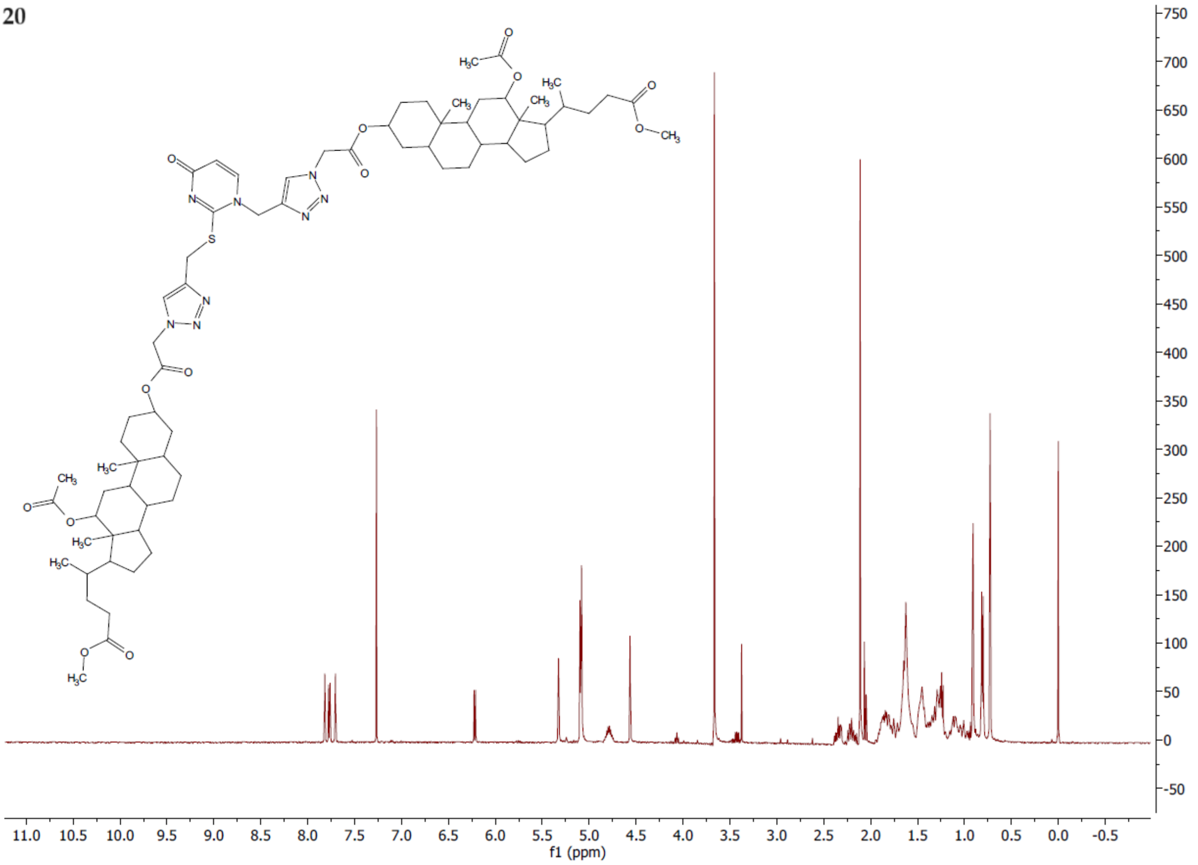

**21**

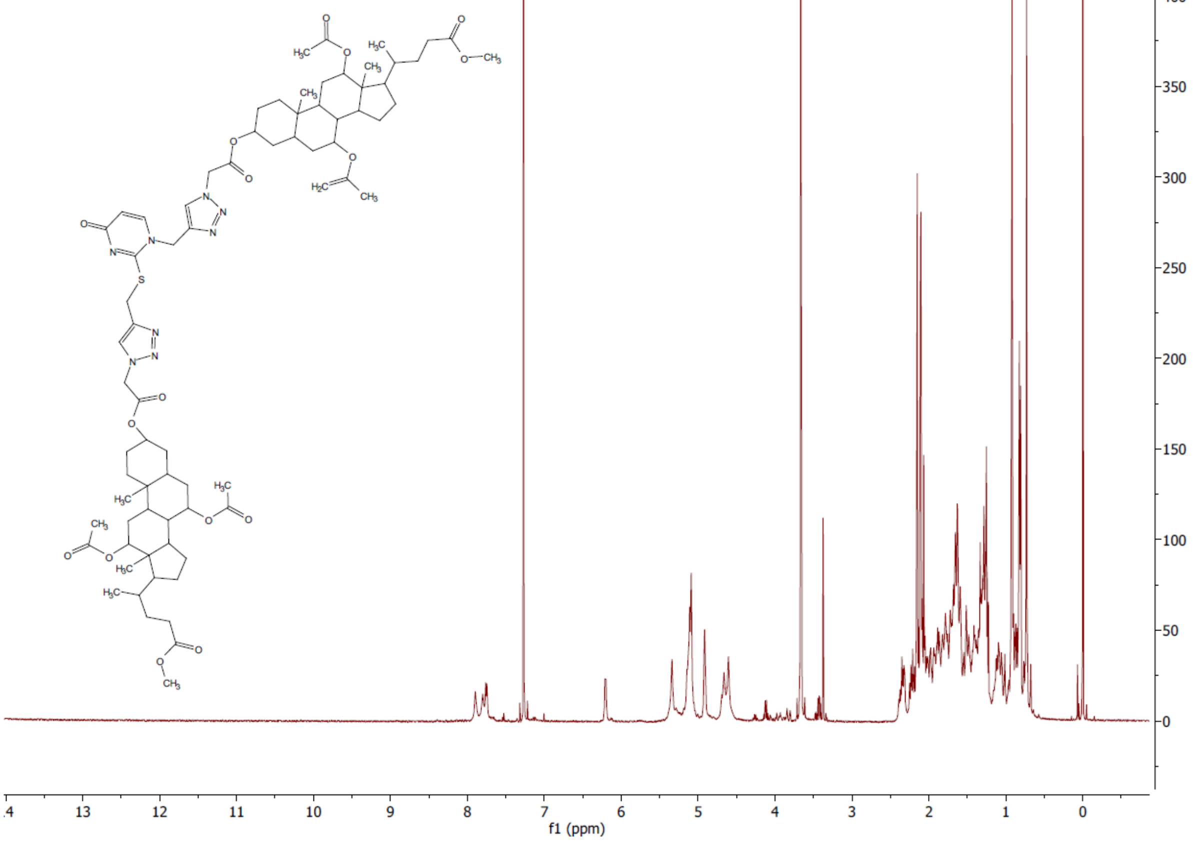


**22**

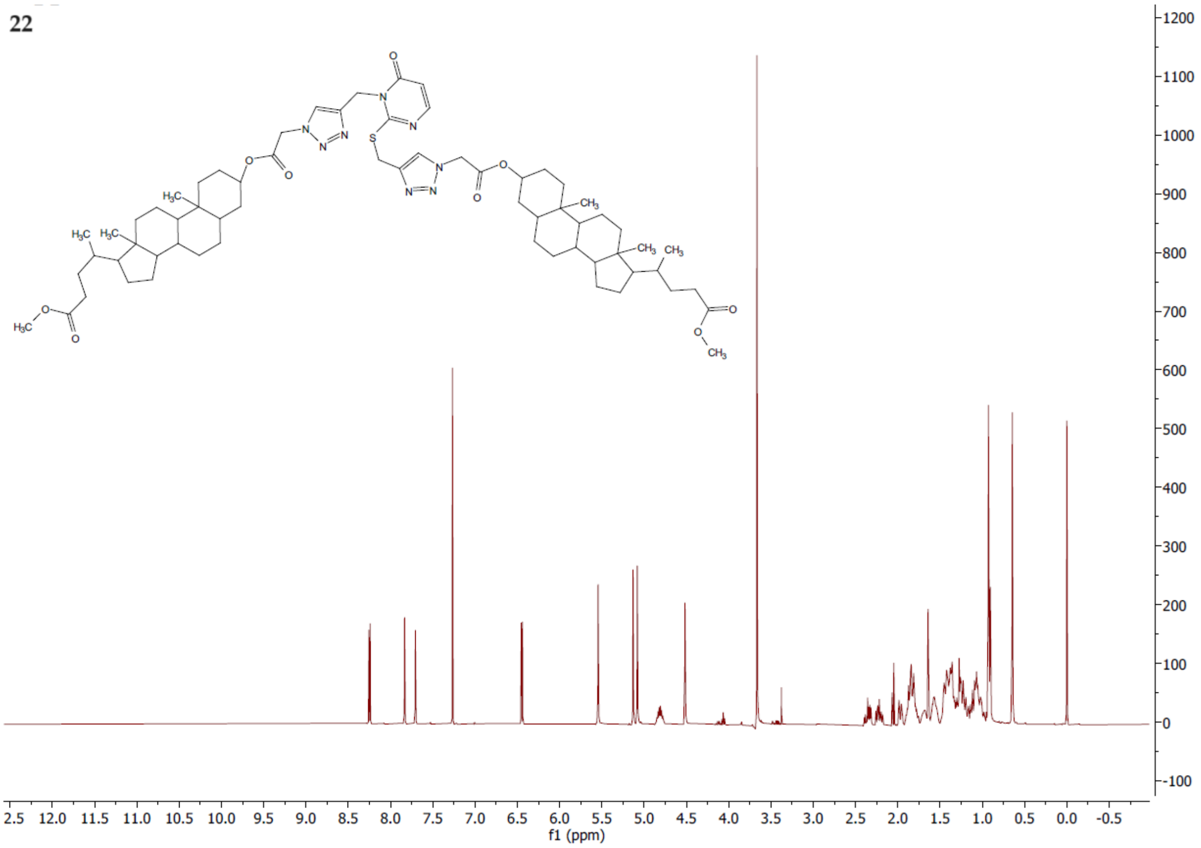

**23**

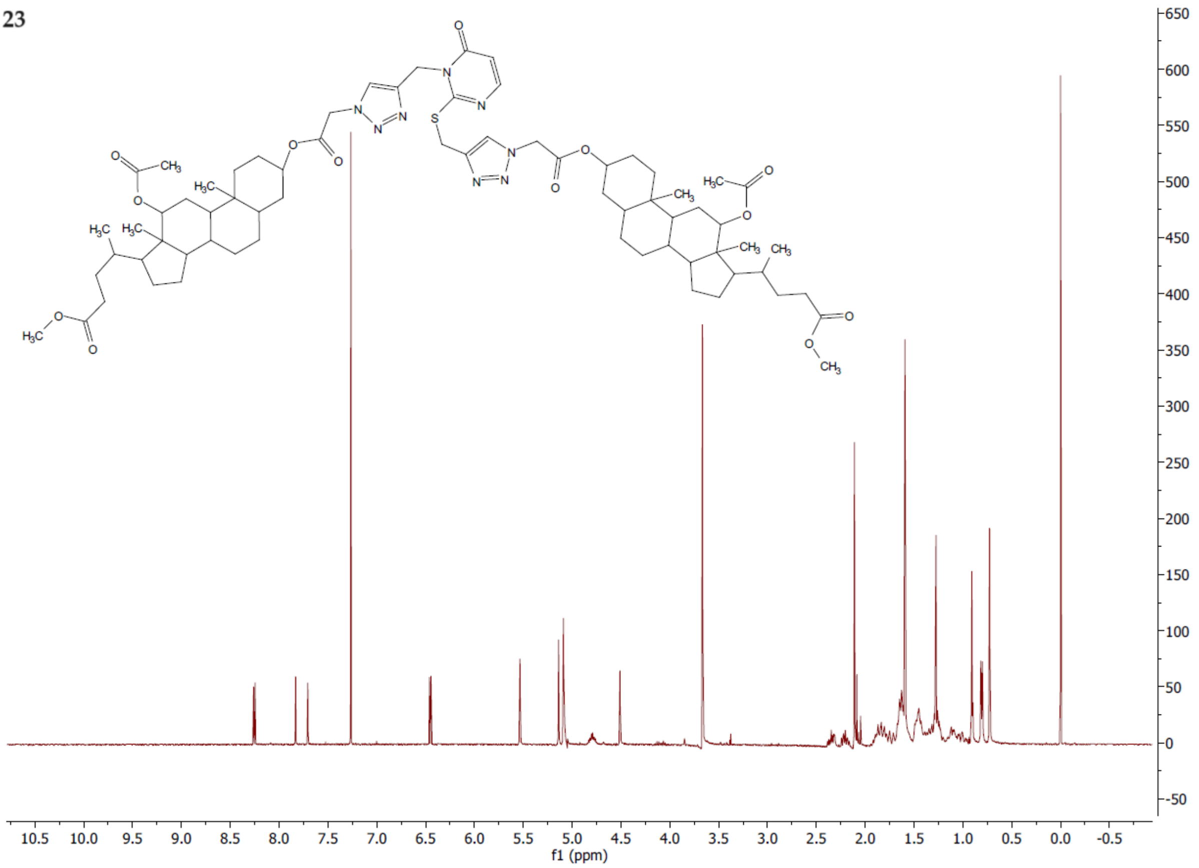


**24**

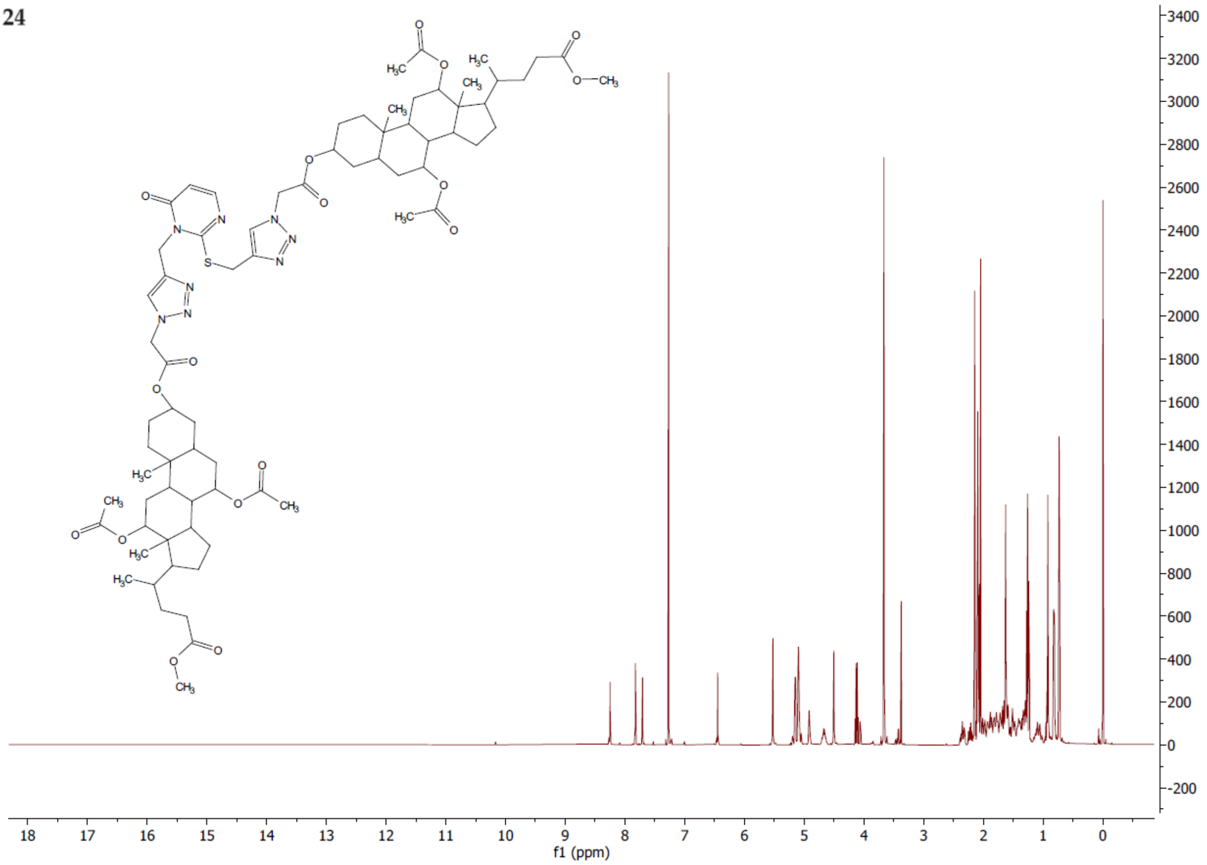

**25**

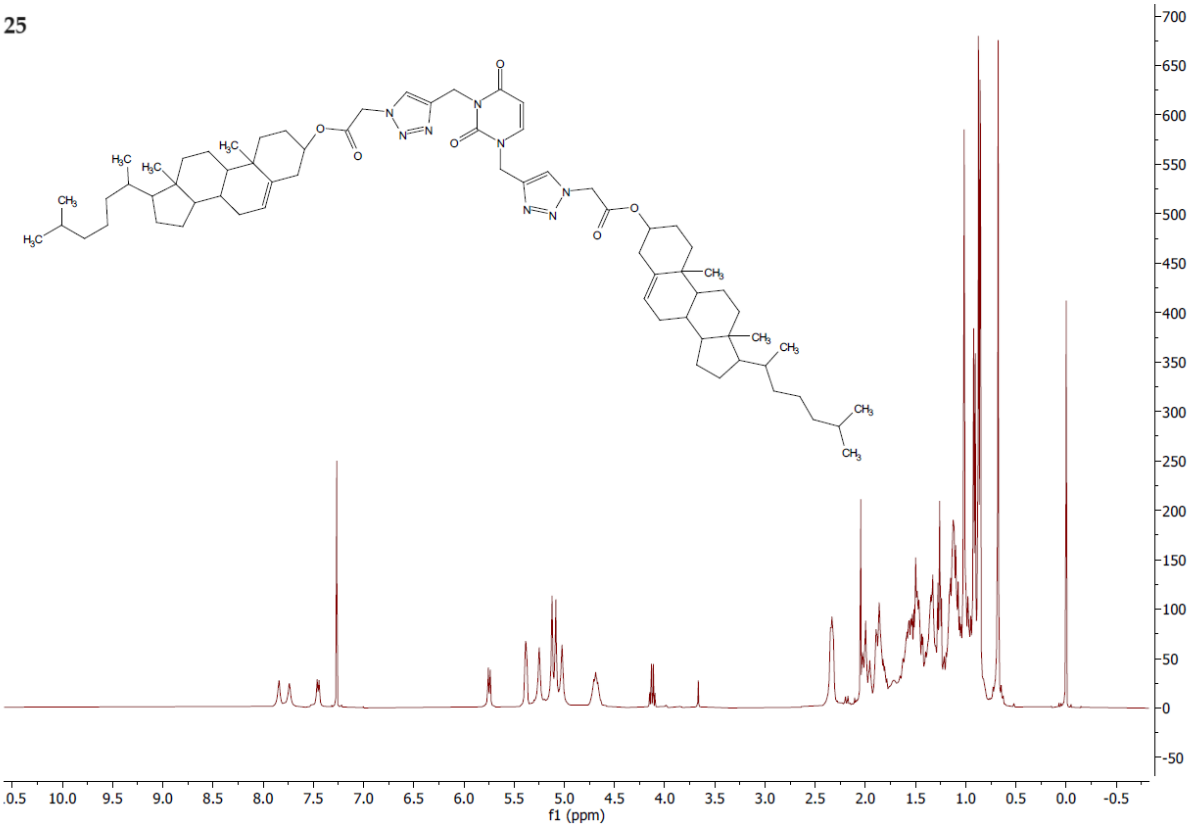


**26**

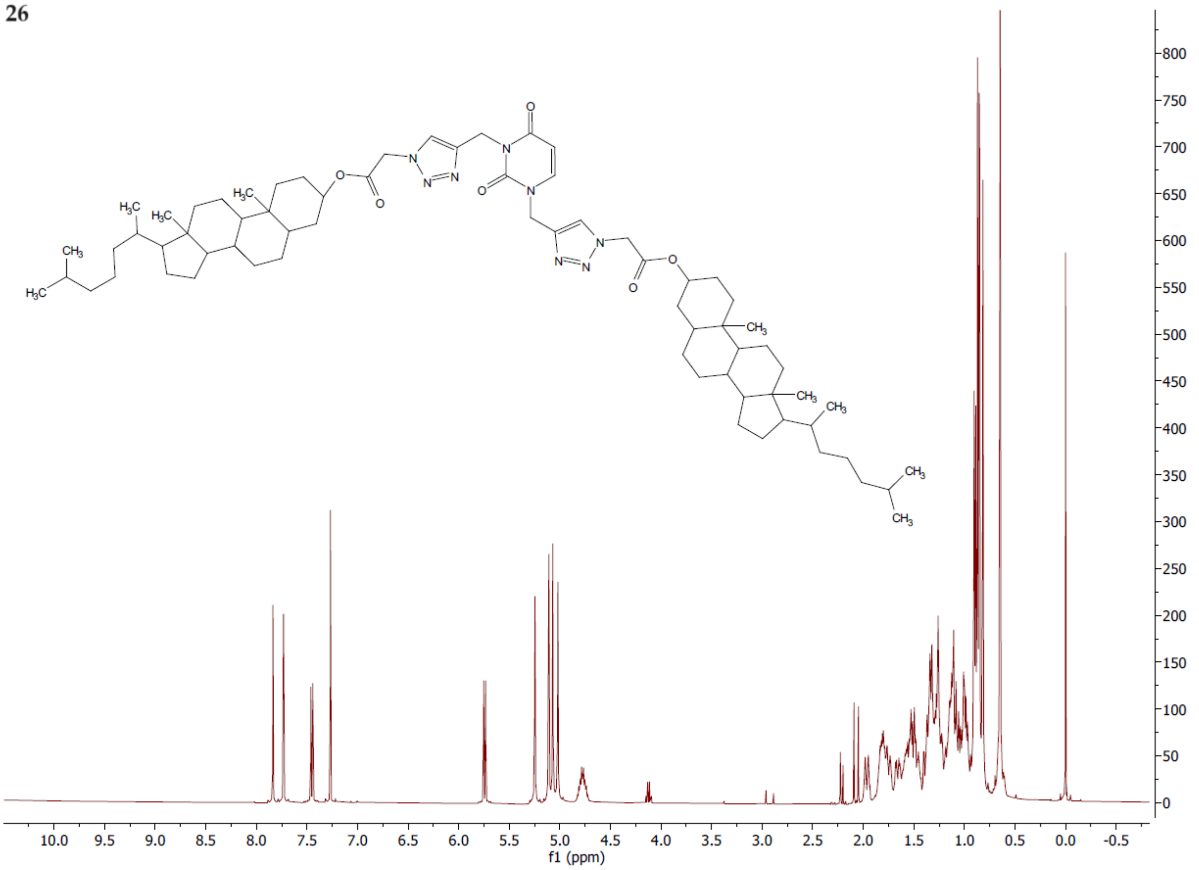

## 2.2. Copies of $^{13}$C NMR spectra

**3**

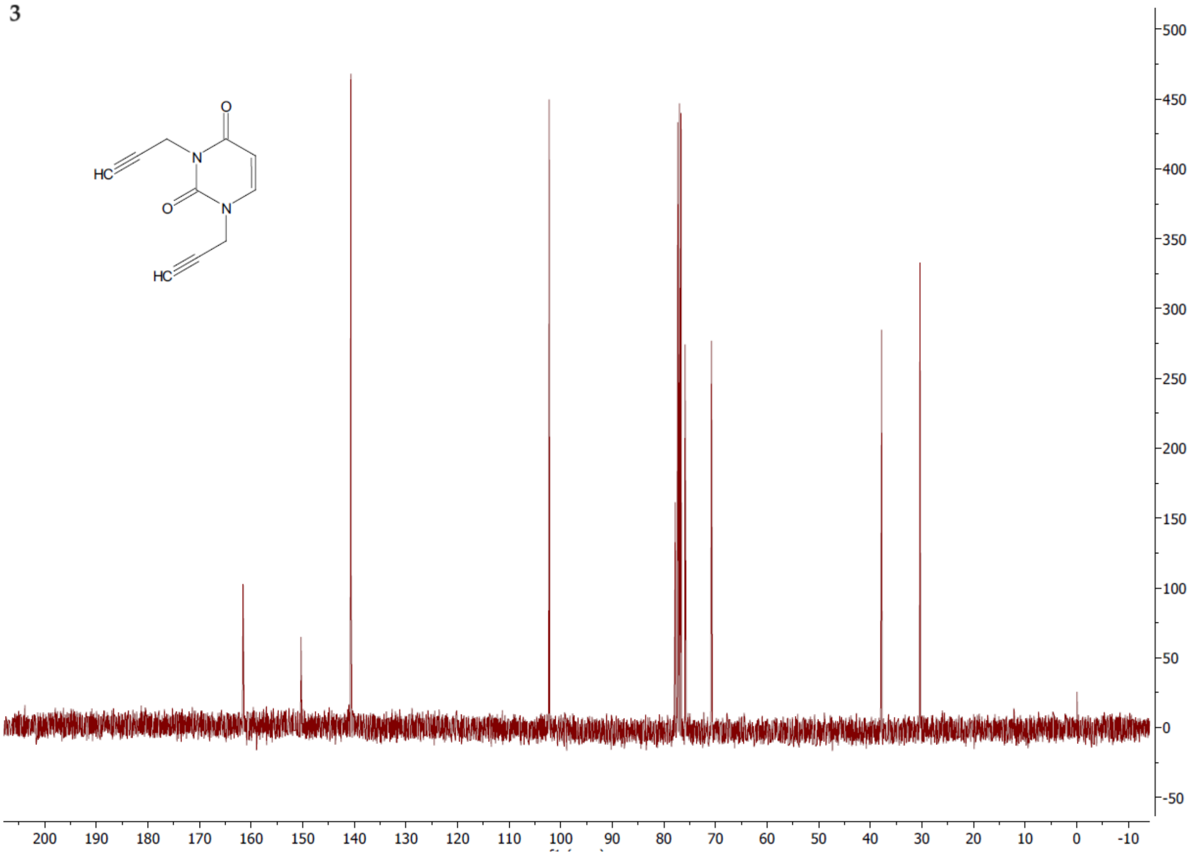


**4**

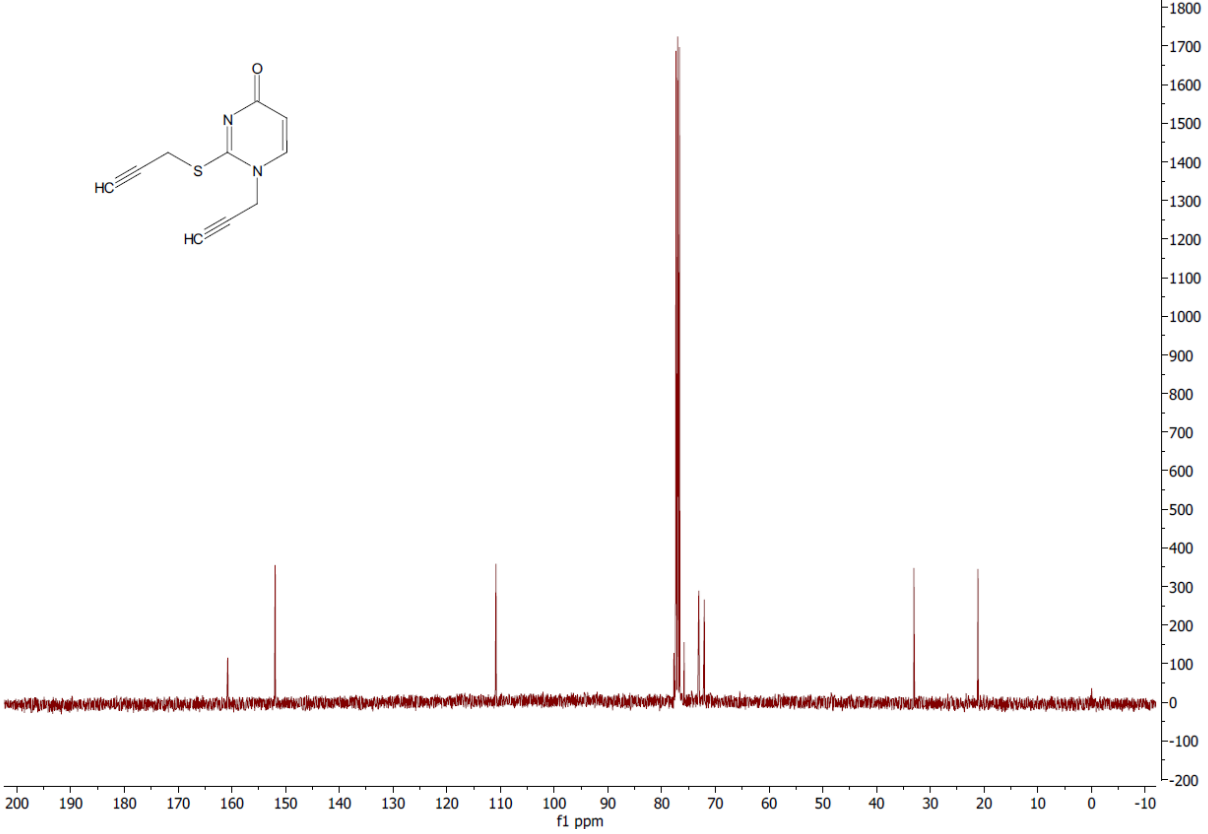

5

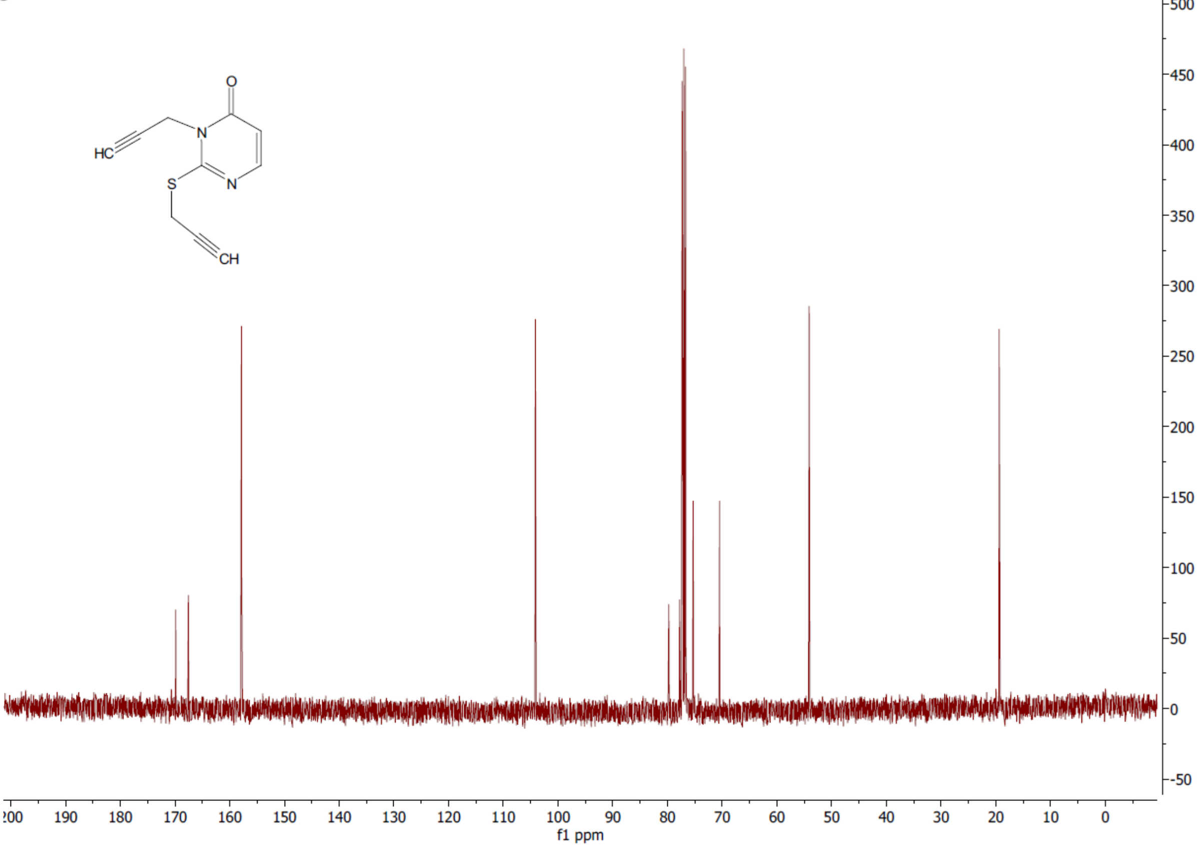


16

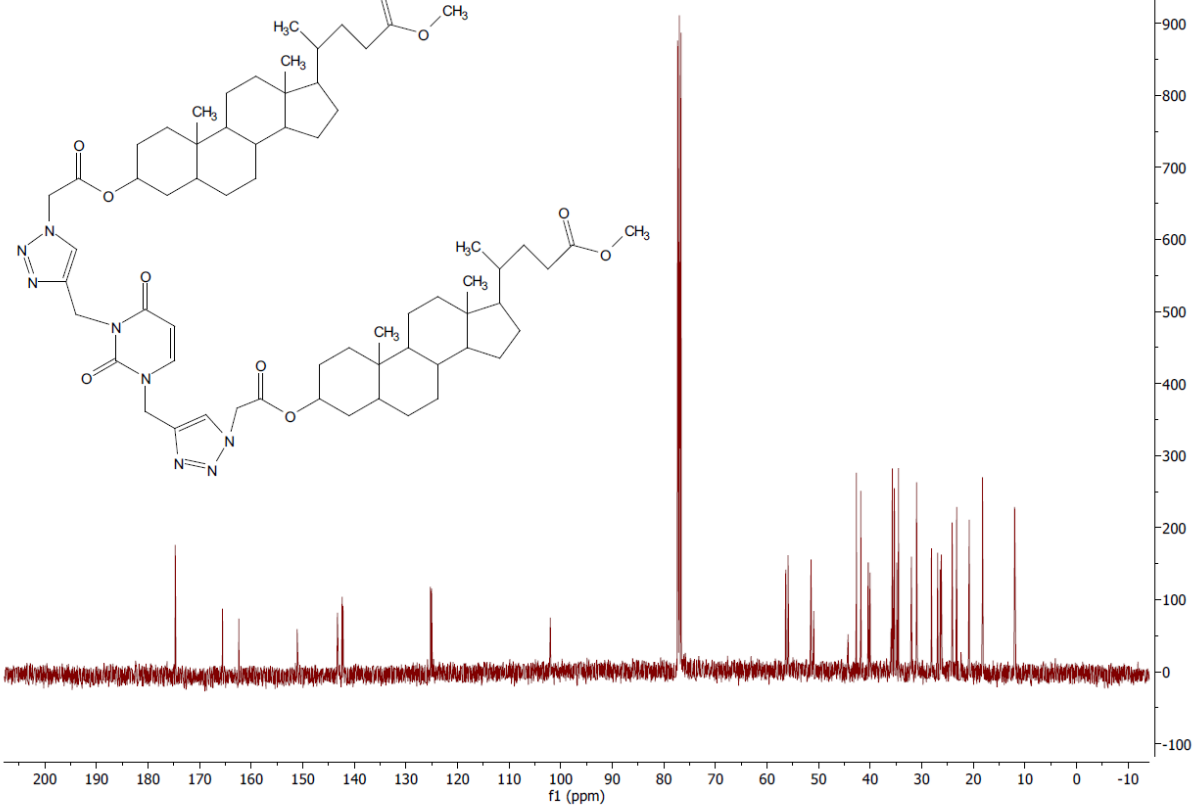

**17**

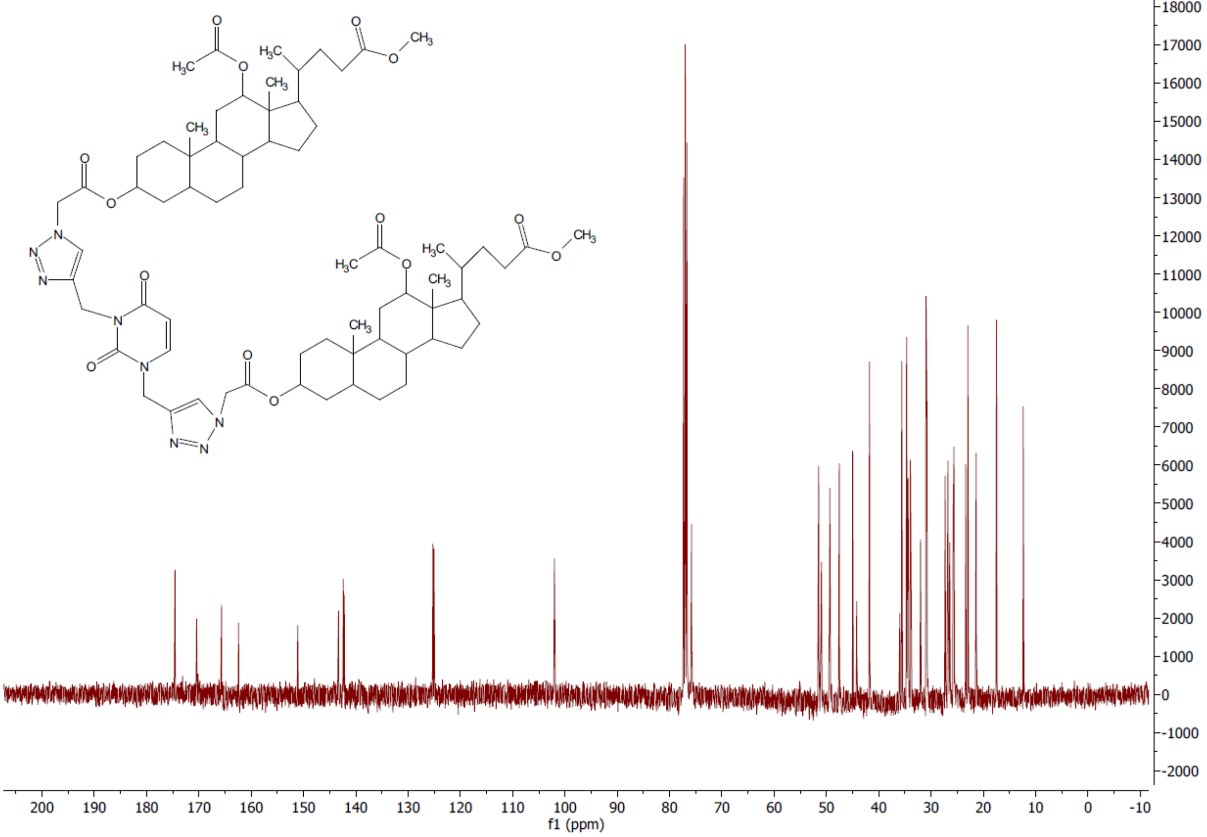


**18**

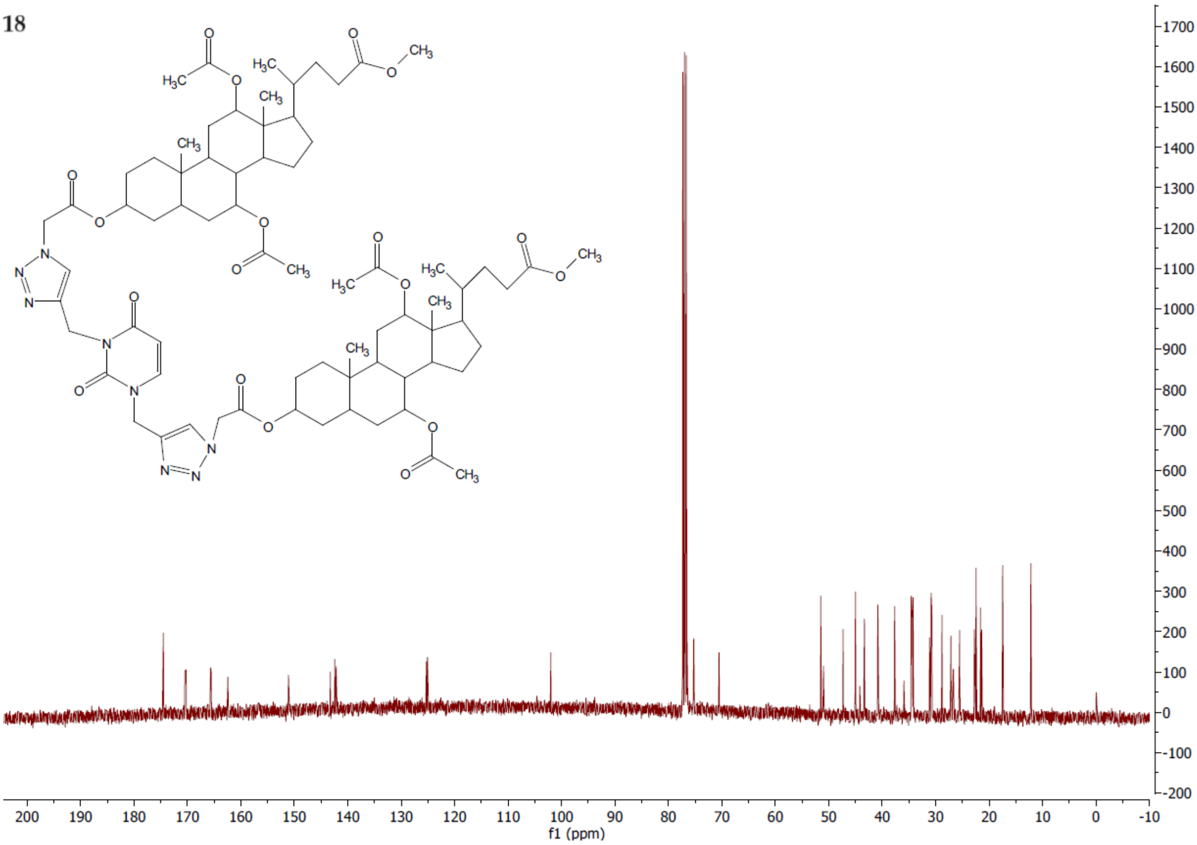

**19**

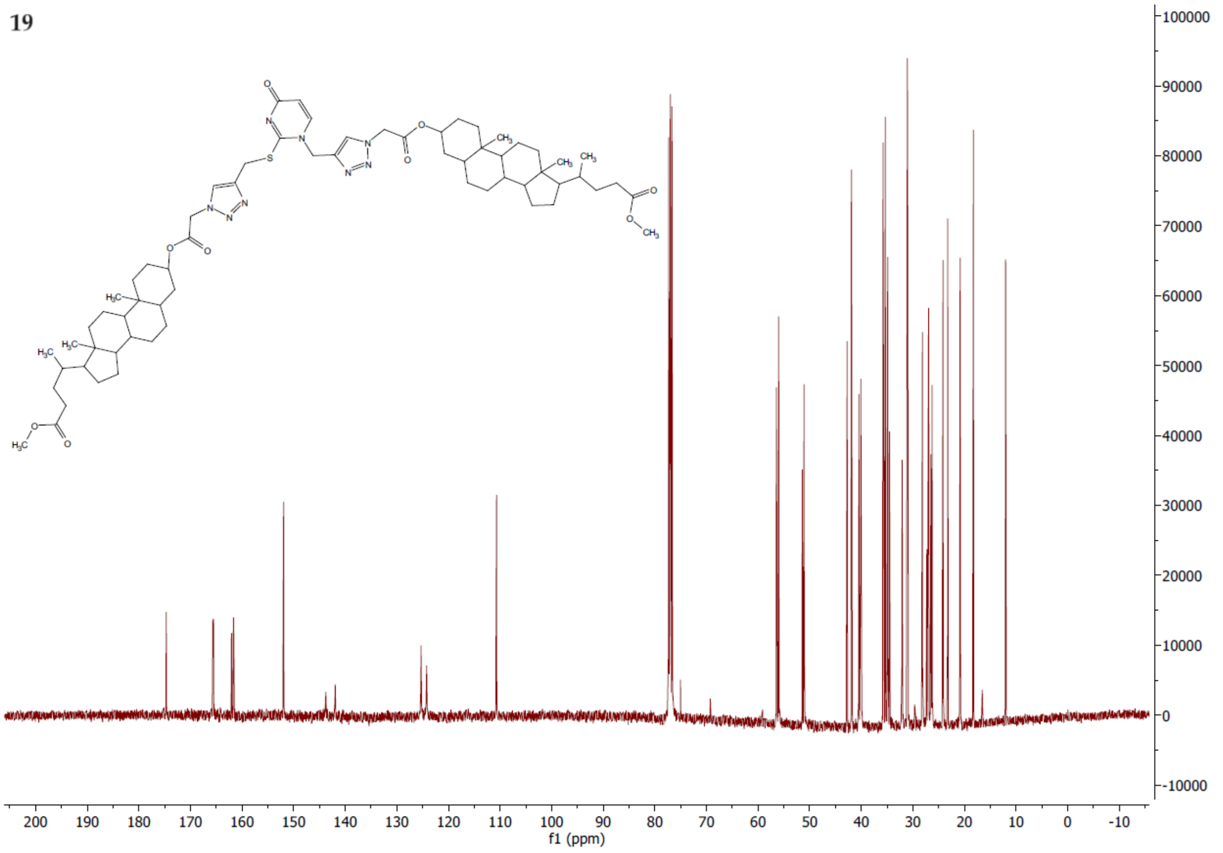

**20**

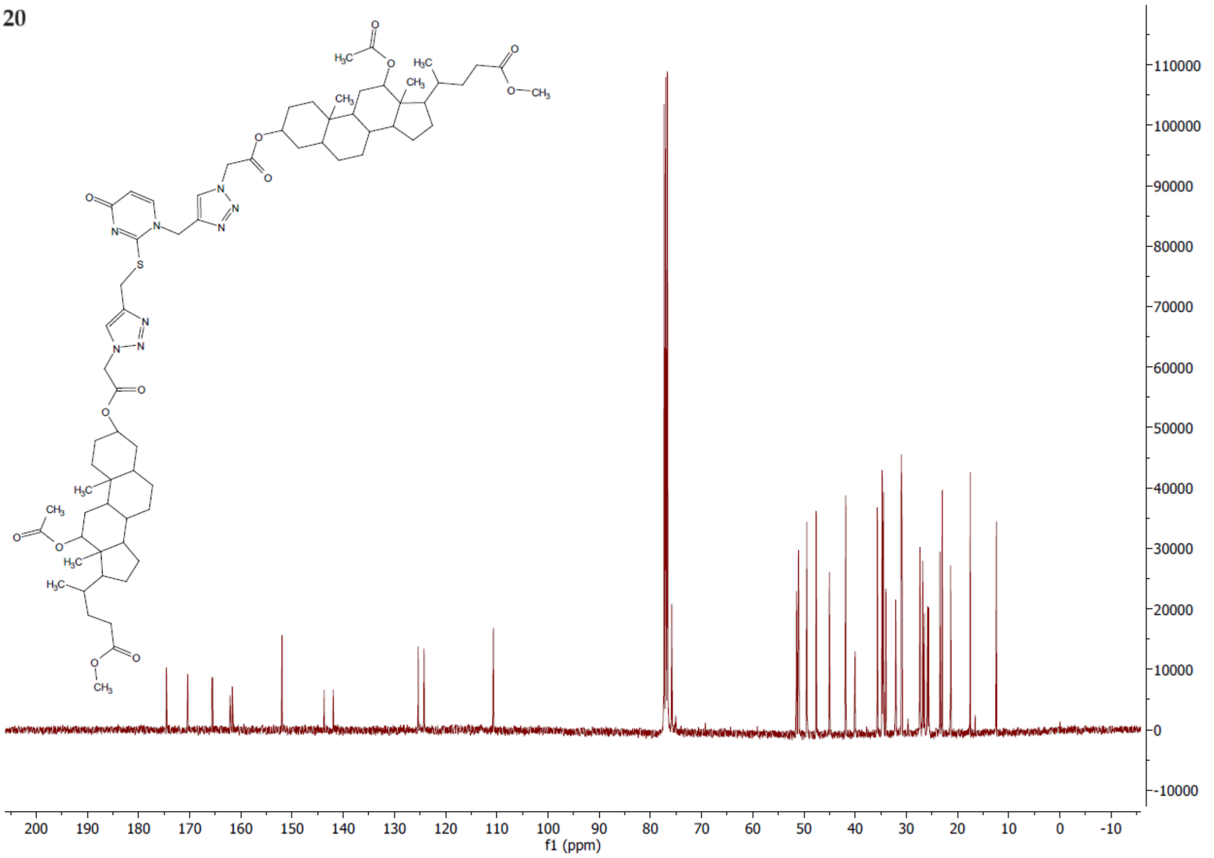

**21**

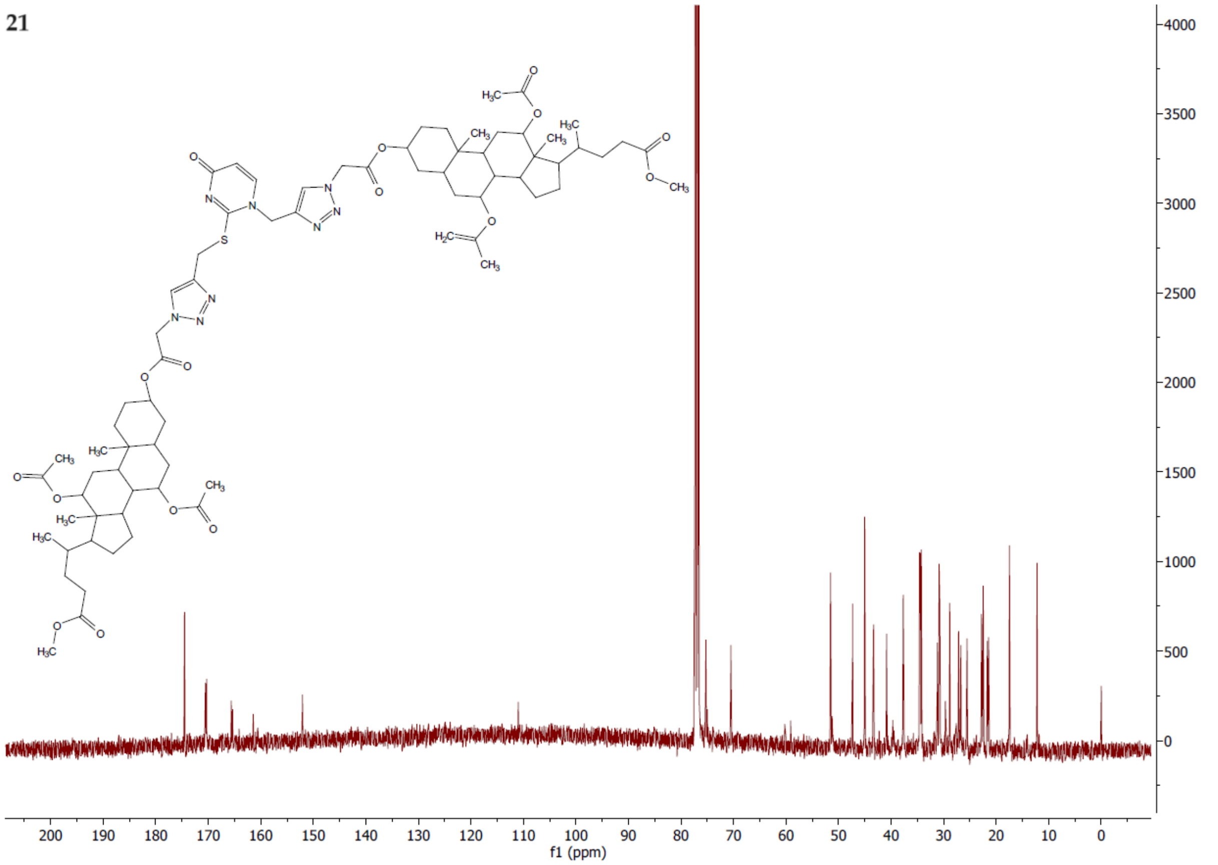


**22**

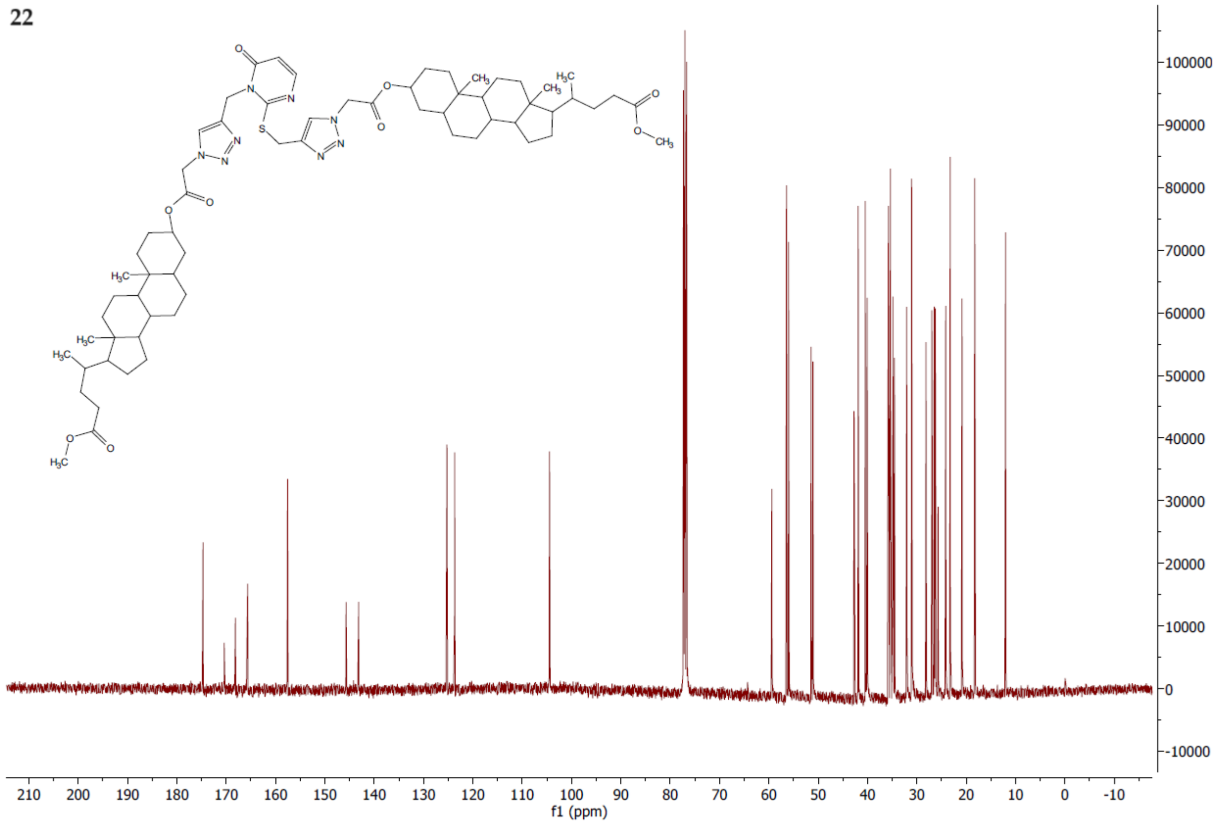

**23**

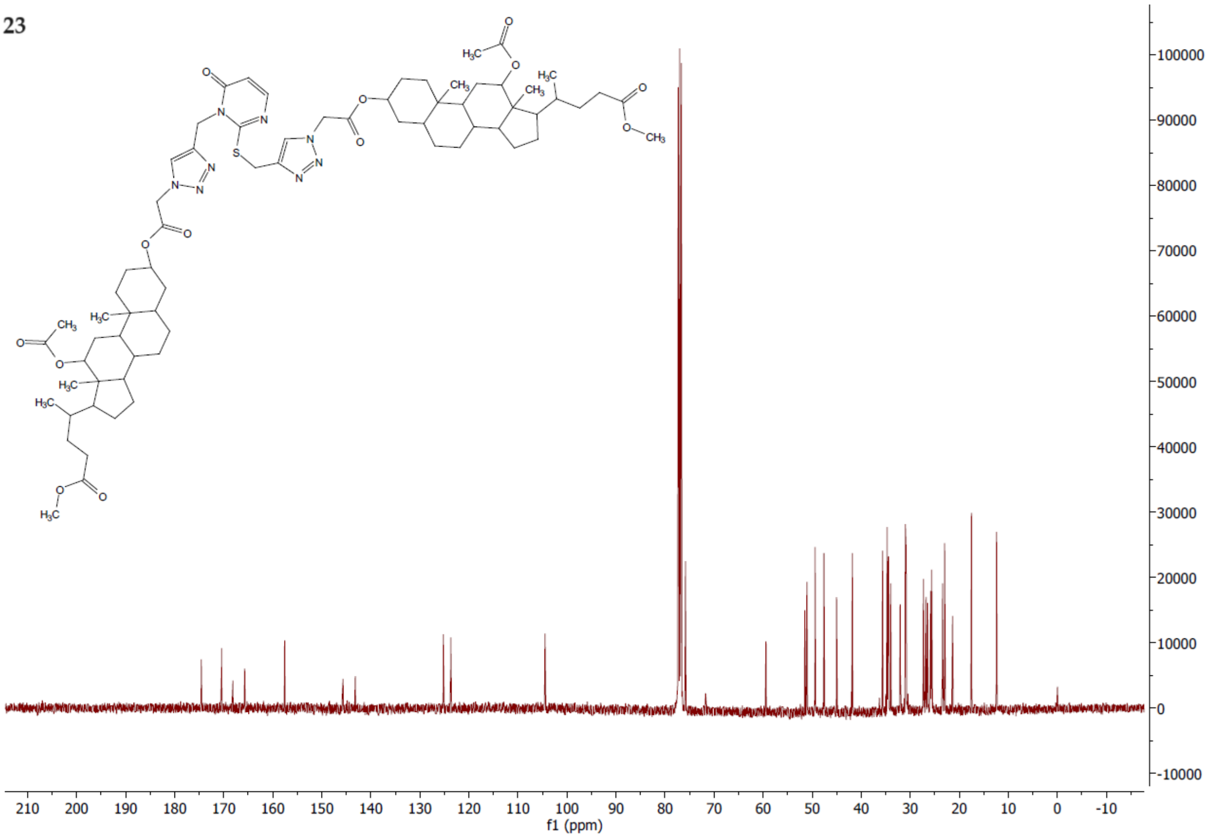

100000
90000
80000
70000
60000
50000
40000
30000
20000
10000
0
-10000
210 200 190 180 170 160 150 140 130 120 110 100 90 80 70 60 50 40 30 20 10 0 -10
f1 (ppm)


**24**

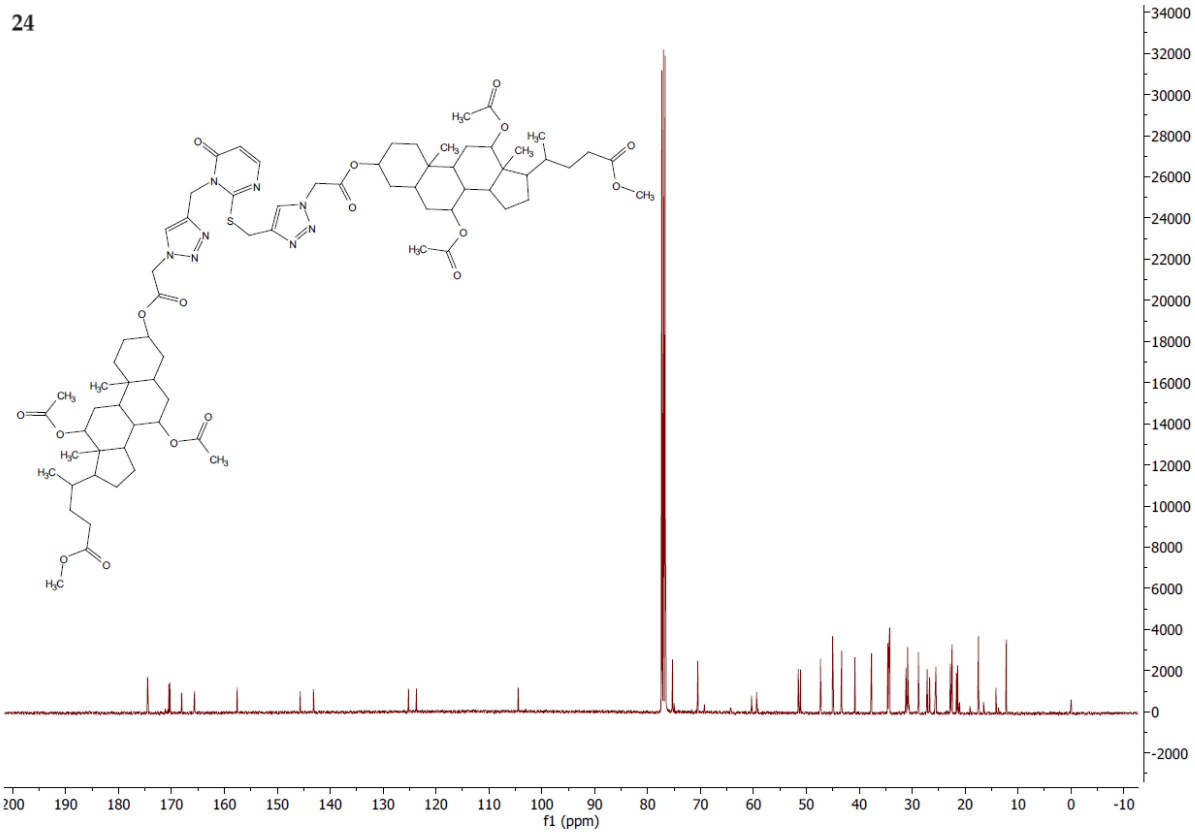

34000
32000
30000
28000
26000
24000
22000
20000
18000
16000
14000
12000
10000
8000
6000
4000
2000
0
-2000
200 190 180 170 160 150 140 130 120 110 100 90 80 70 60 50 40 30 20 10 0 -10
f1 (ppm)

**25**

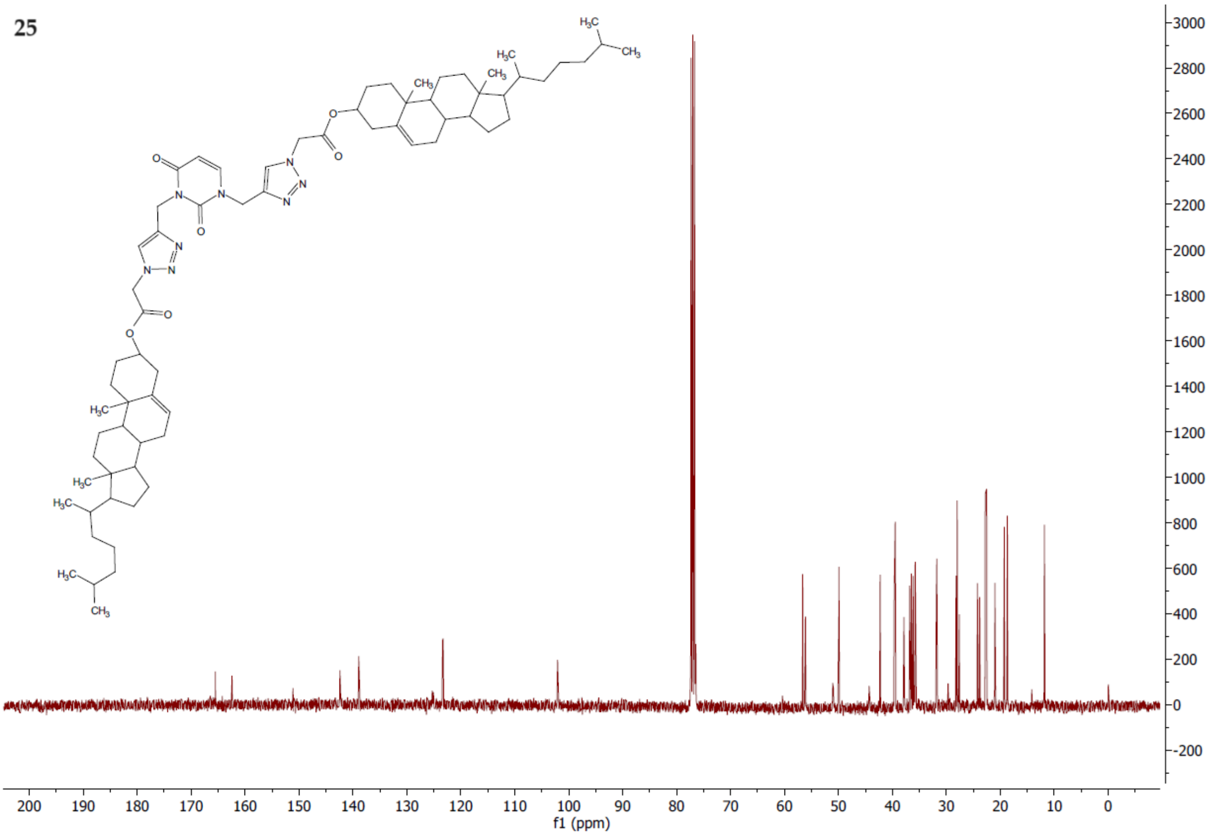

**26**

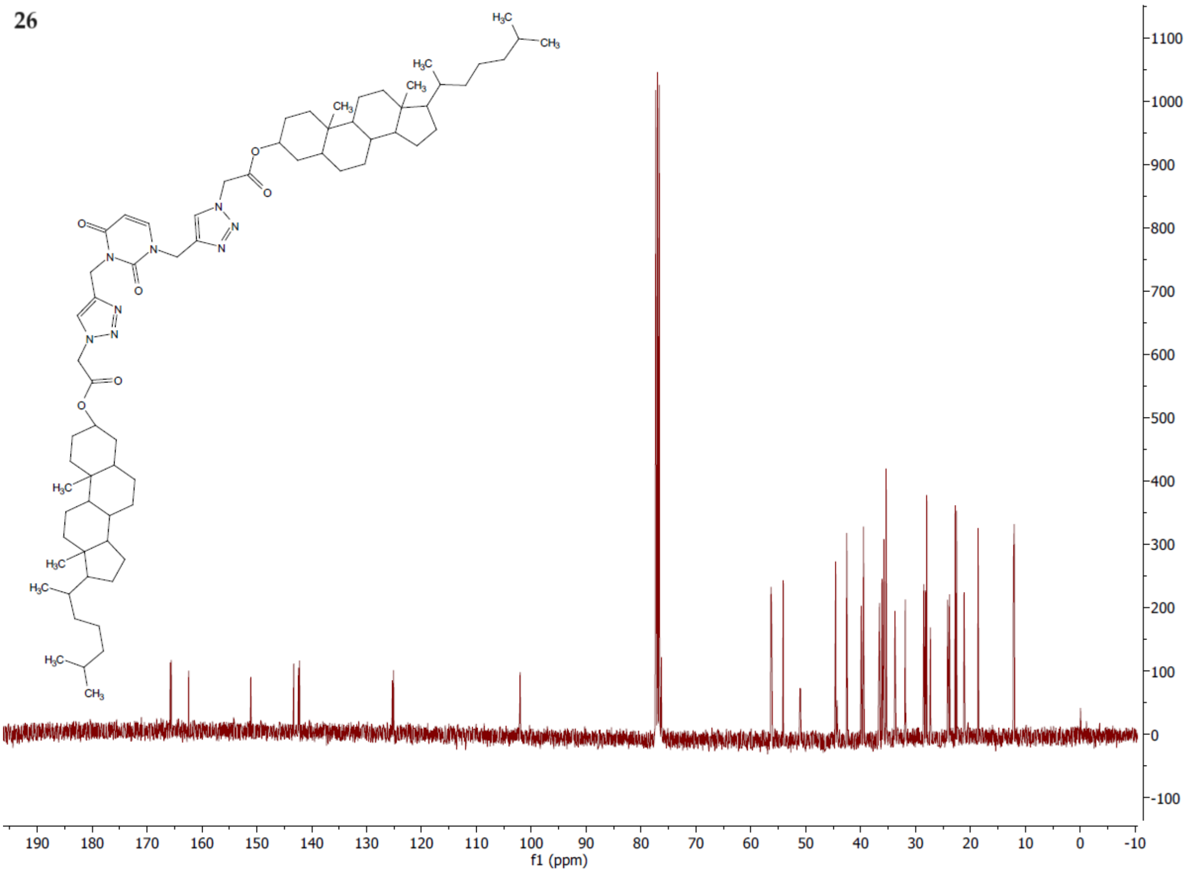

## 3. Copies of MS spectra

### 3.1. Copies of ESI-MS spectra

**16**

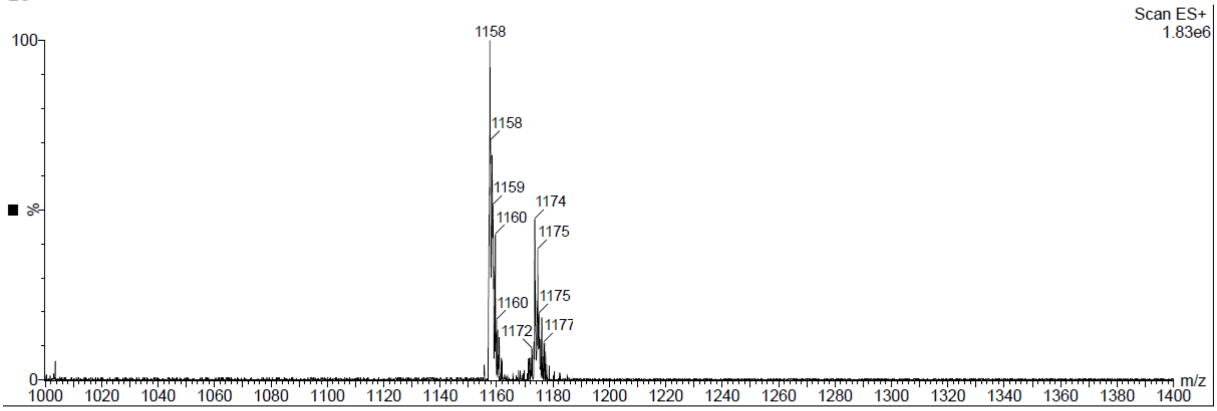


**17**

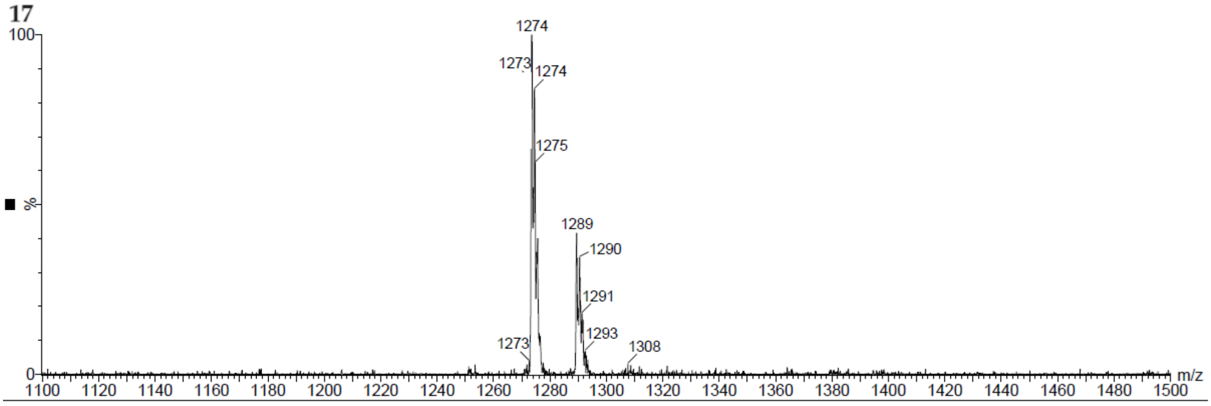


**18**

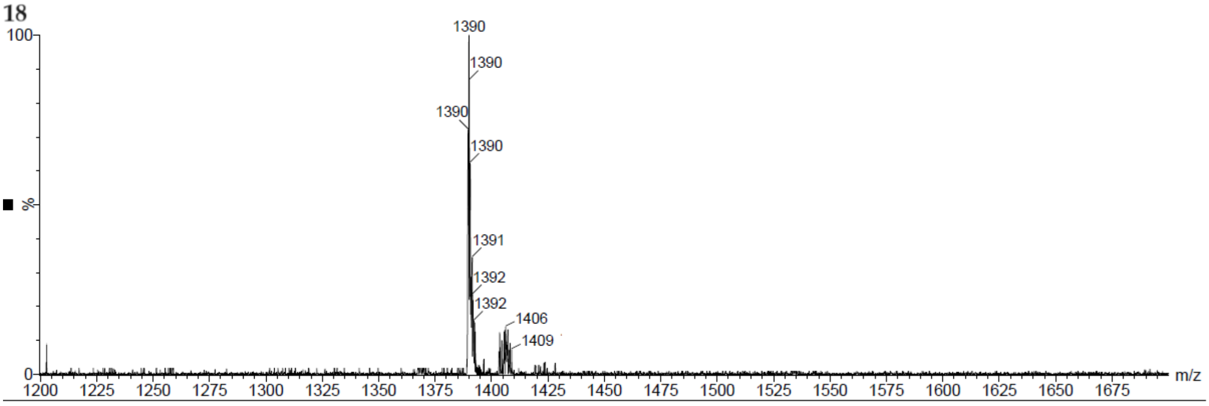


**19**

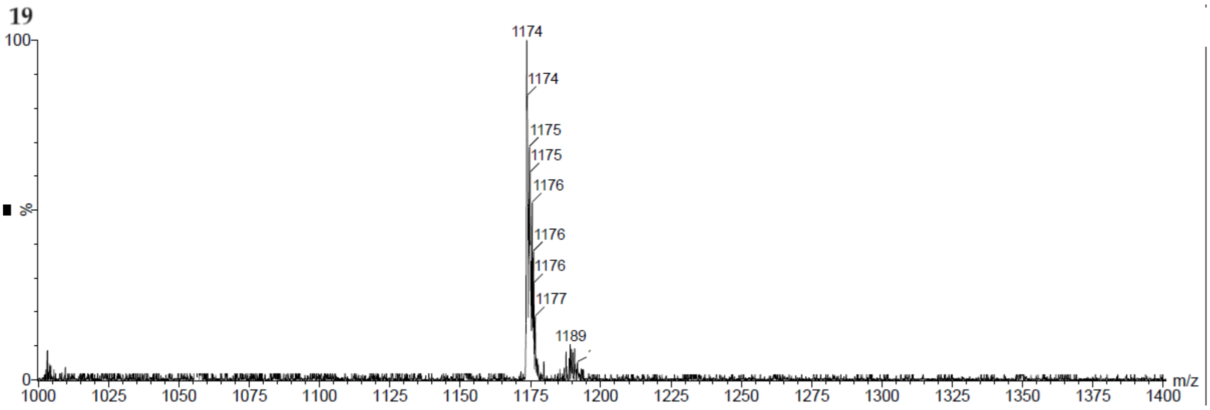

**20**

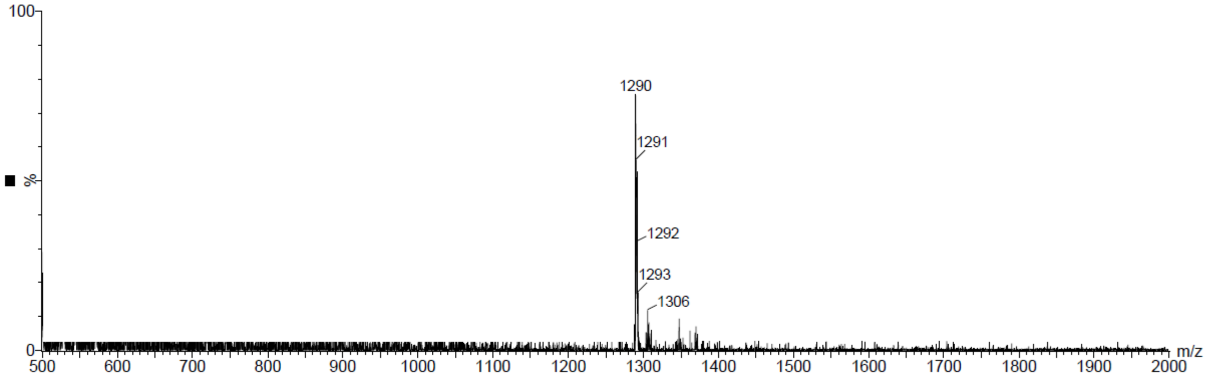
100
%
0
1290
1291
1292
1293
1306
500 600 700 800 900 1000 1100 1200 1300 1400 1500 1600 1700 1800 1900 2000
m/z

**22**

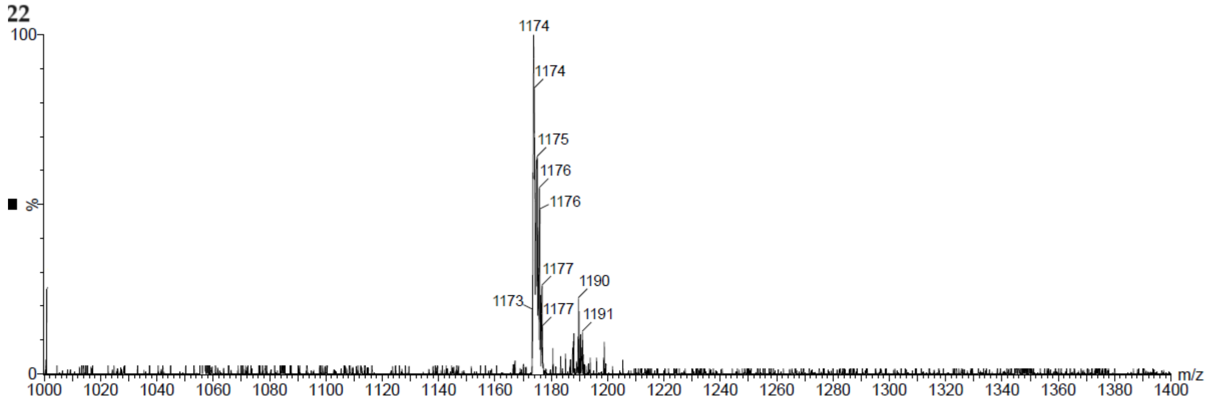
100
%
0
1174
1174
1175
1176
1176
1177
1173
1177
1190
1191
1000 1020 1040 1060 1080 1100 1120 1140 1160 1180 1200 1220 1240 1260 1280 1300 1320 1340 1360 1380 1400
m/z

**23**

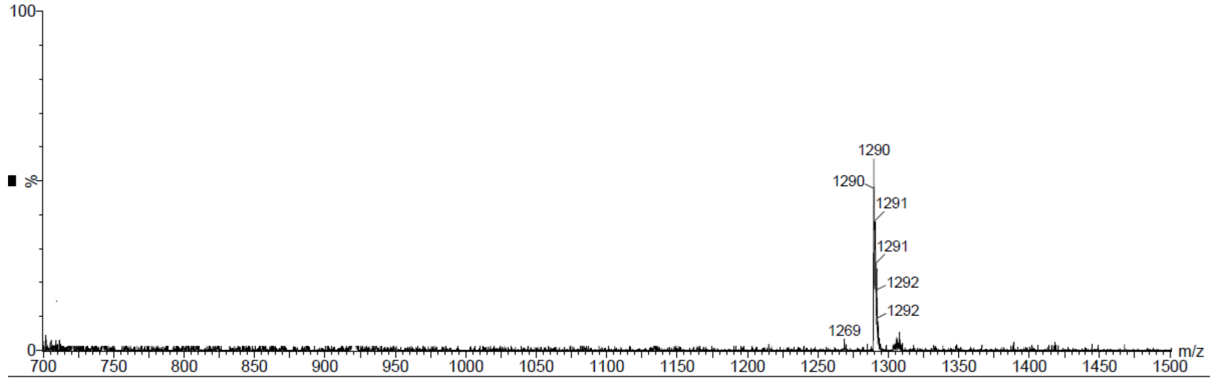
100
%
0
1290
1290
1291
1291
1292
1292
1269
700 750 800 850 900 950 1000 1050 1100 1150 1200 1250 1300 1350 1400 1450 1500
m/z

**24**

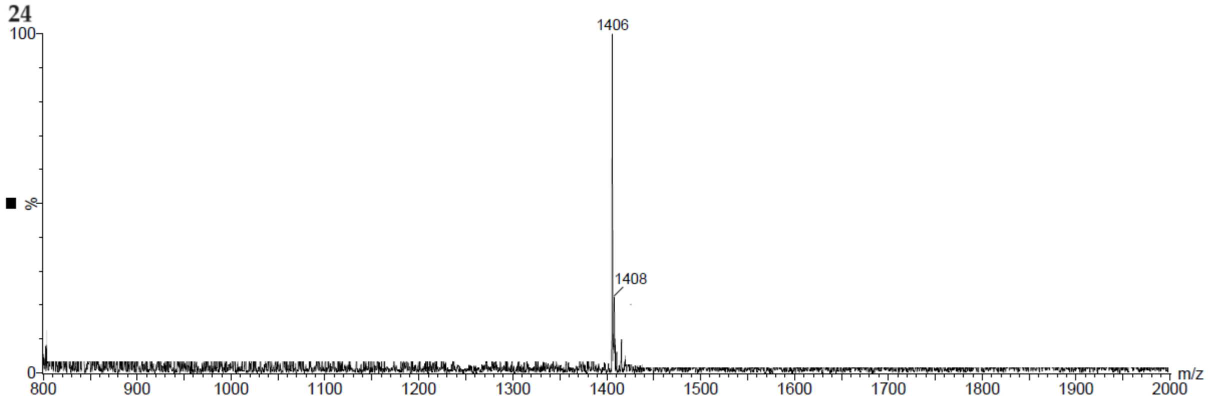
100
%
0
1406
1408
800 900 1000 1100 1200 1300 1400 1500 1600 1700 1800 1900 2000
m/z

25

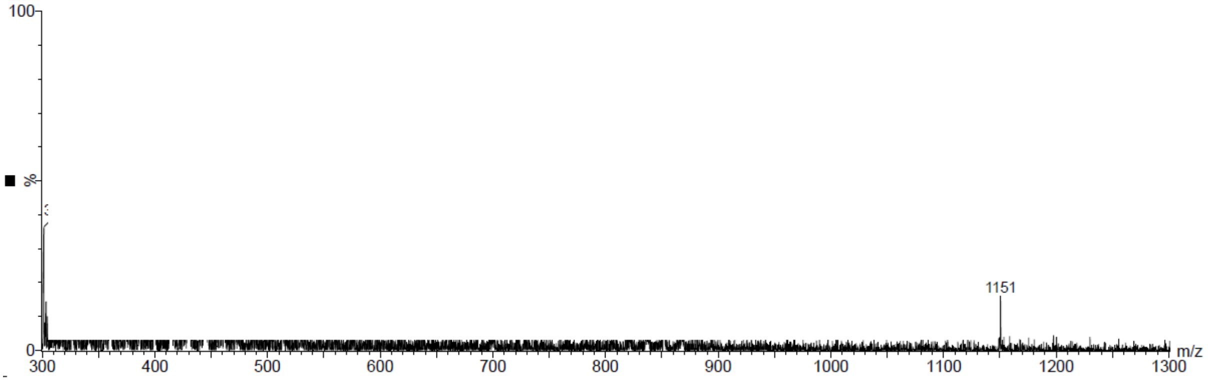


26

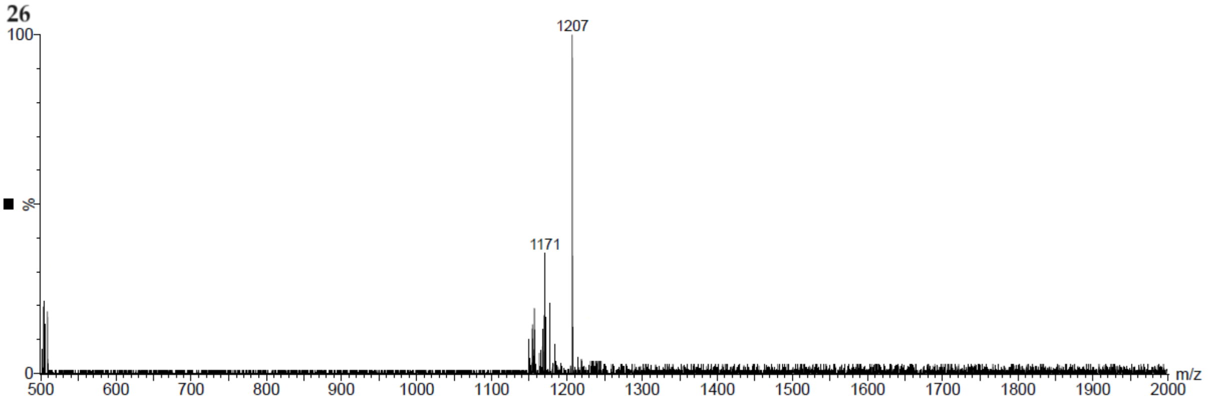


## 3.2. Copies of EI-MS spectra

4

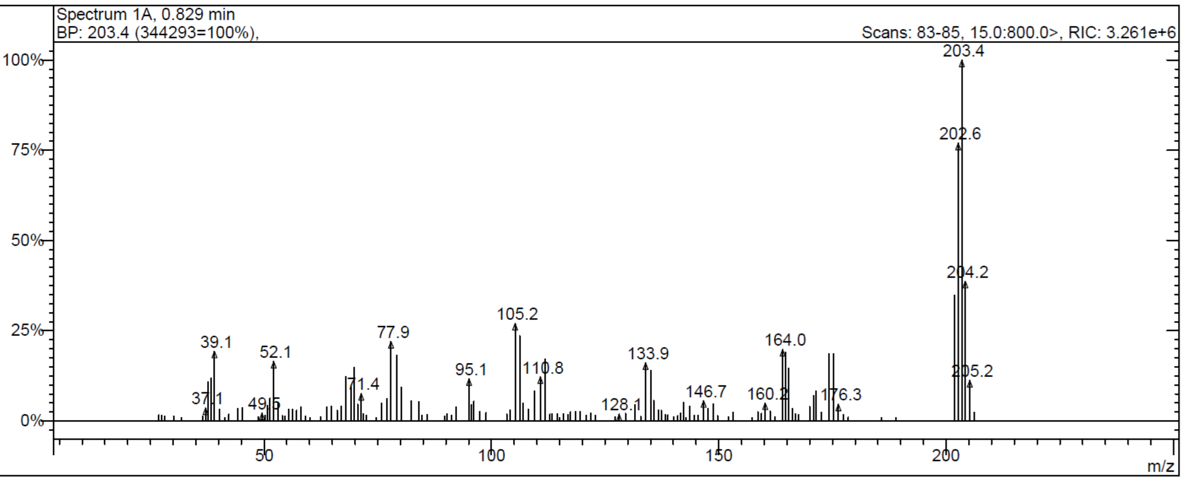


5

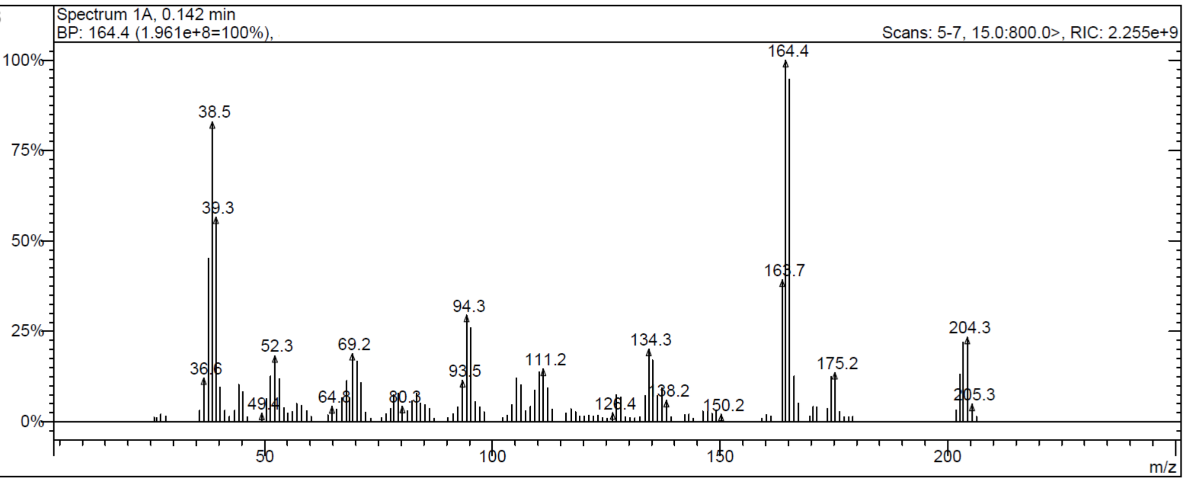

## 4. Copies of FT-IR spectra

### 16

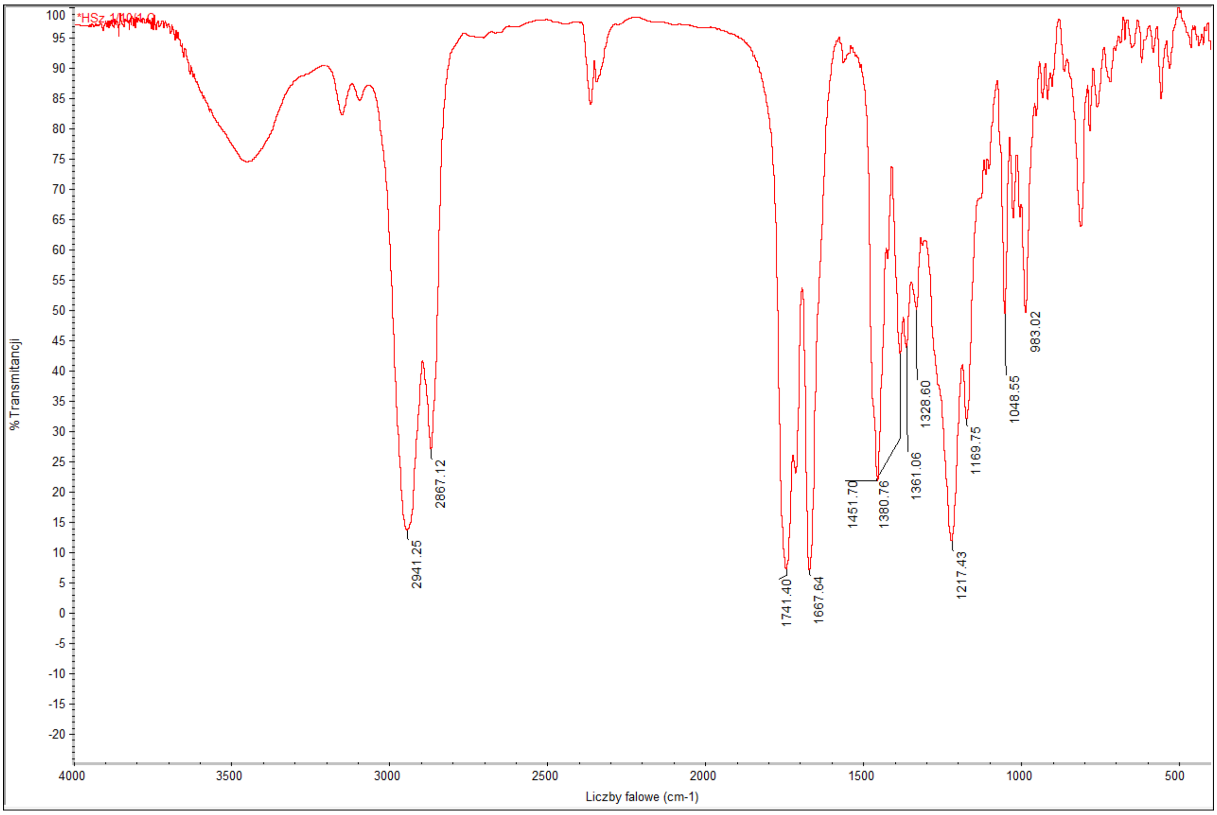


### 17

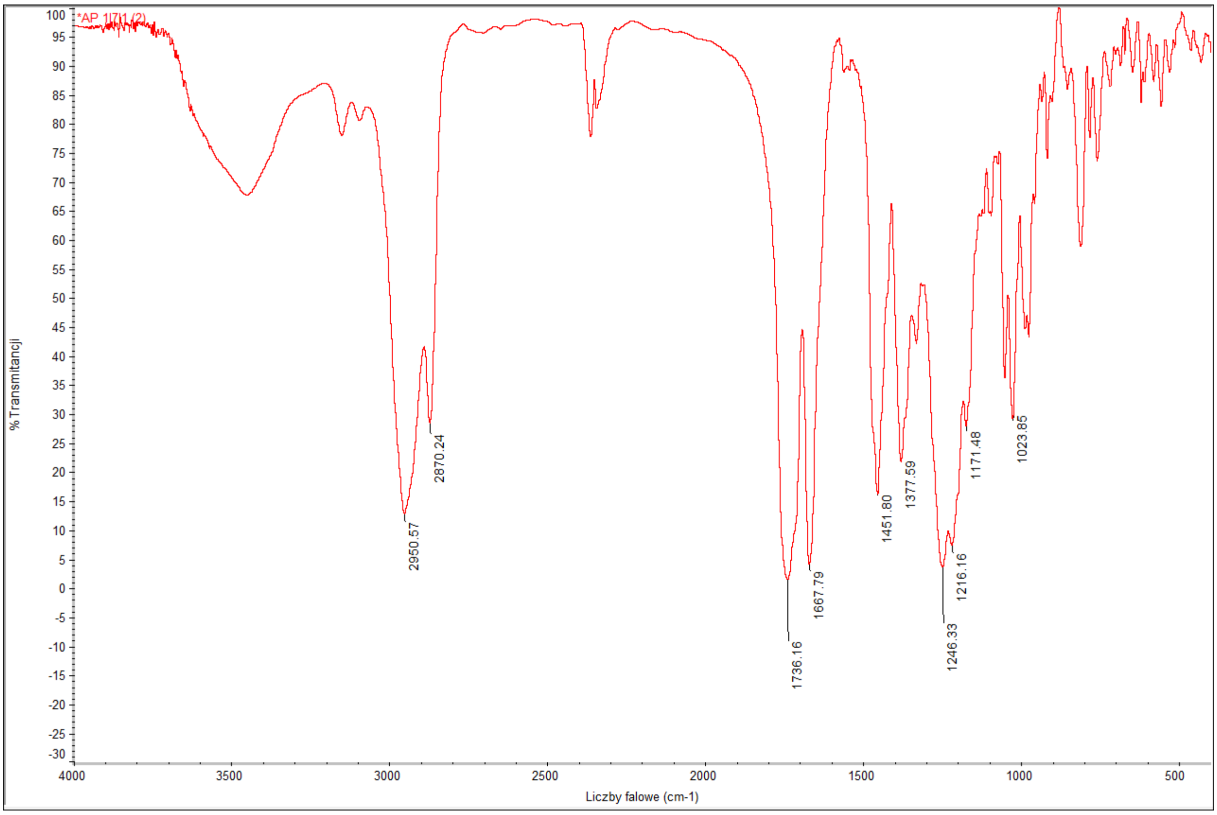

**18**

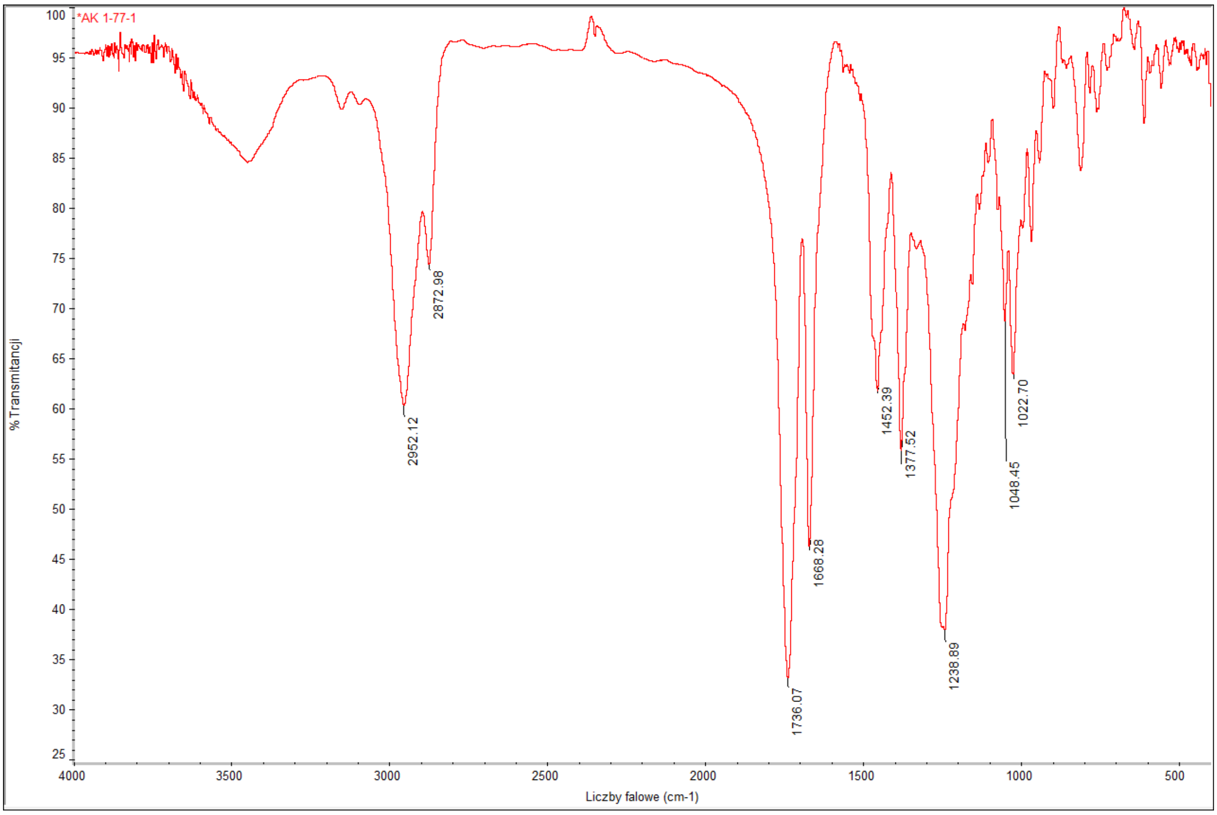
*AK 1-77-1
% Transmitancji
Liczby falowe (cm-1)
2952.12
2872.98
1736.07
1668.28
1452.39
1377.52
1238.89
1048.45
1022.70

**19**

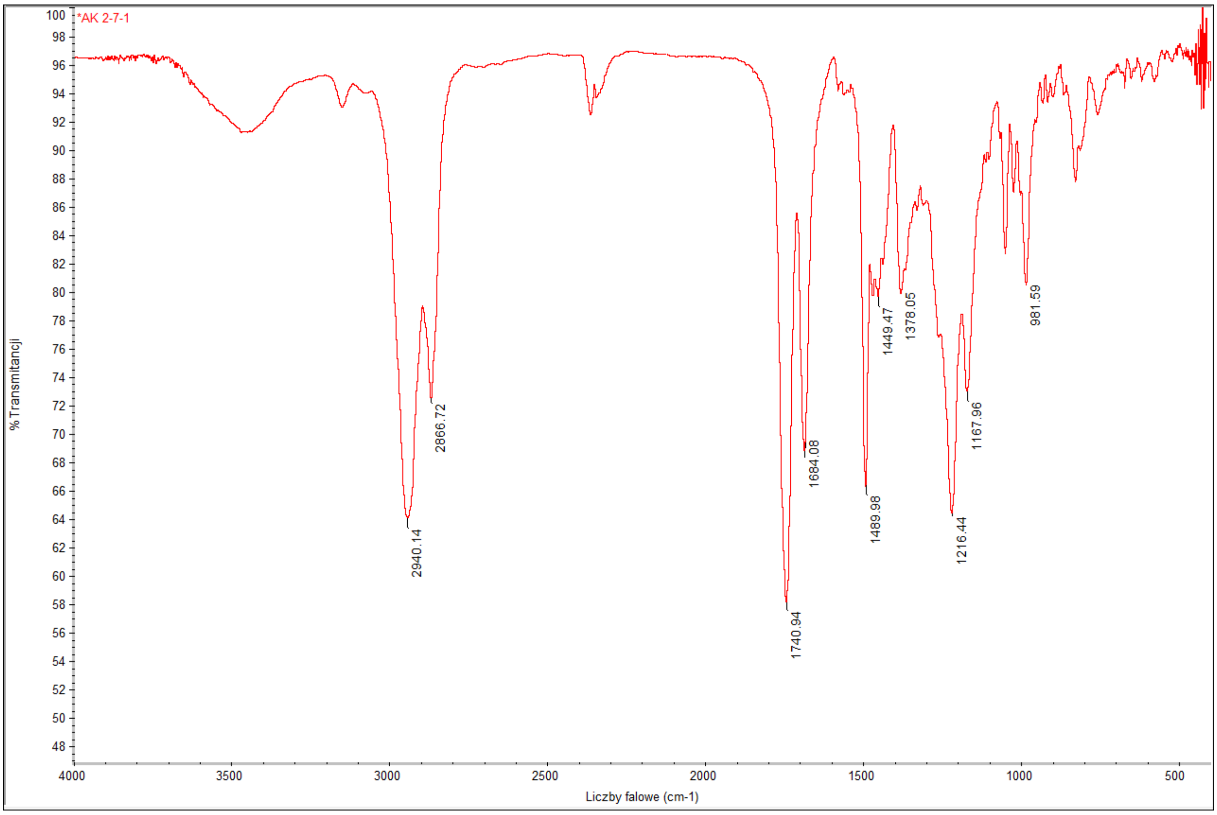
*AK 2-7-1
% Transmitancji
Liczby falowe (cm-1)
2940.14
2866.72
1740.94
1684.08
1489.98
1449.47
1378.05
1216.44
1167.96
981.59

**20**

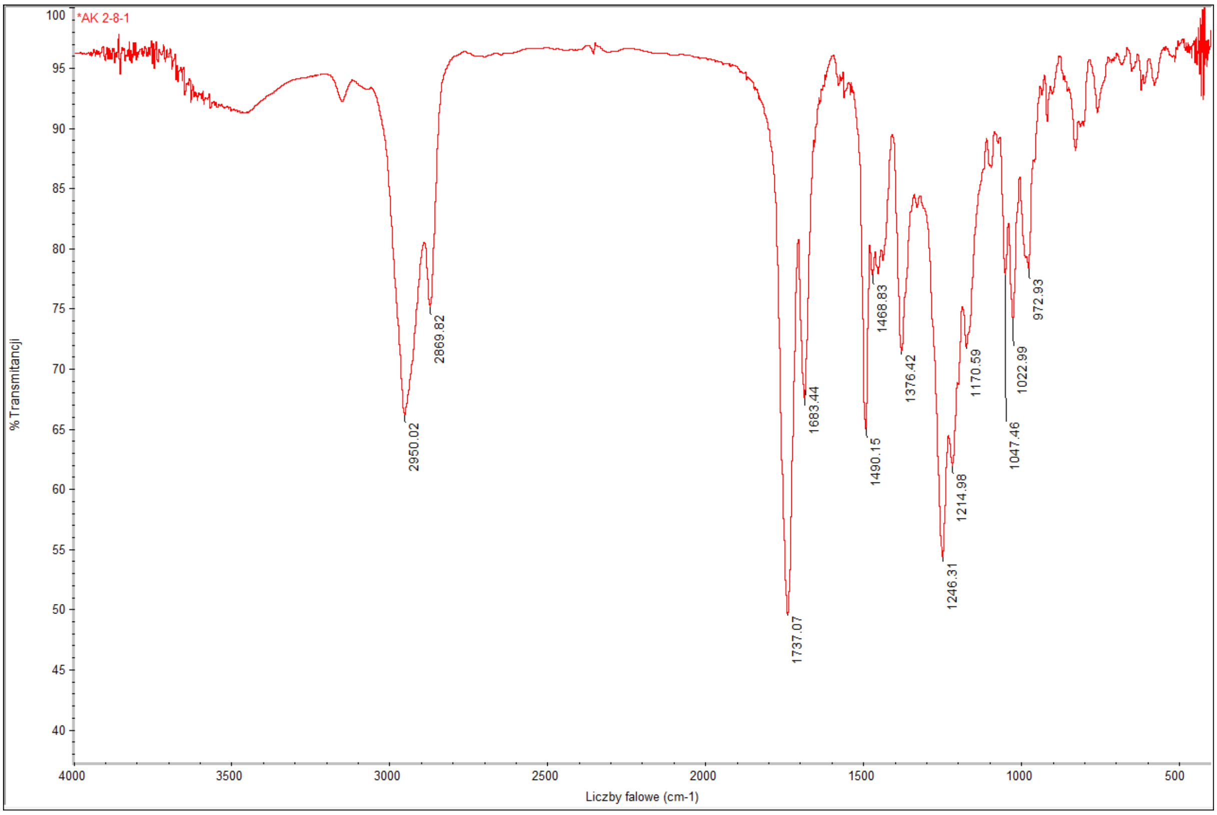

*AK 2-8-1
% Transmitancji
Liczby falowe (cm-1)
100
95
90
85
80
75
70
65
60
55
50
45
40
4000
3500
3000
2500
2000
1500
1000
500
2950.02
2869.82
1737.07
1683.44
1490.15
1468.83
1376.42
1246.31
1214.98
1170.59
1047.46
1022.99
972.93


**21**

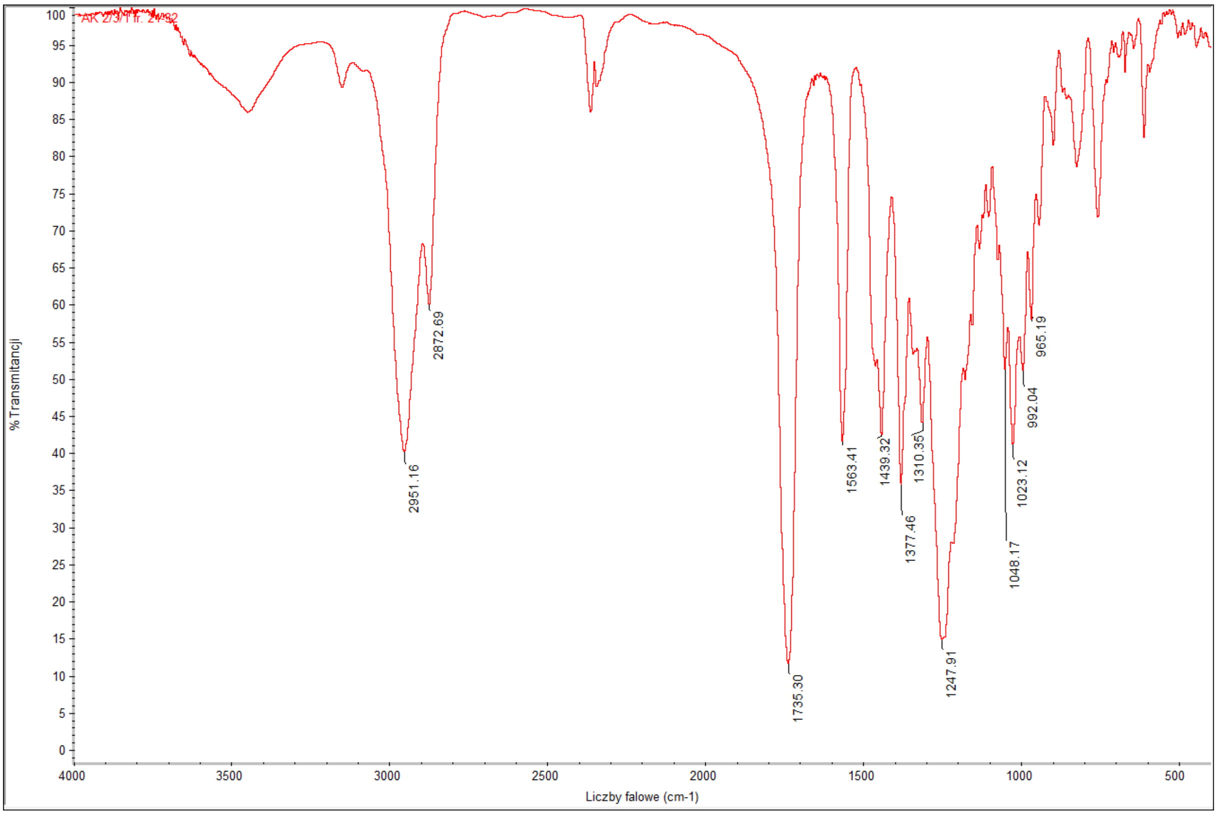

% Transmitancji
Liczby falowe (cm-1)
100
95
90
85
80
75
70
65
60
55
50
45
40
35
30
25
20
15
10
5
0
4000
3500
3000
2500
2000
1500
1000
500
2951.16
2872.69
1735.30
1563.41
1439.32
1377.46
1310.35
1247.91
1048.17
1023.12
992.04
965.19

**22**

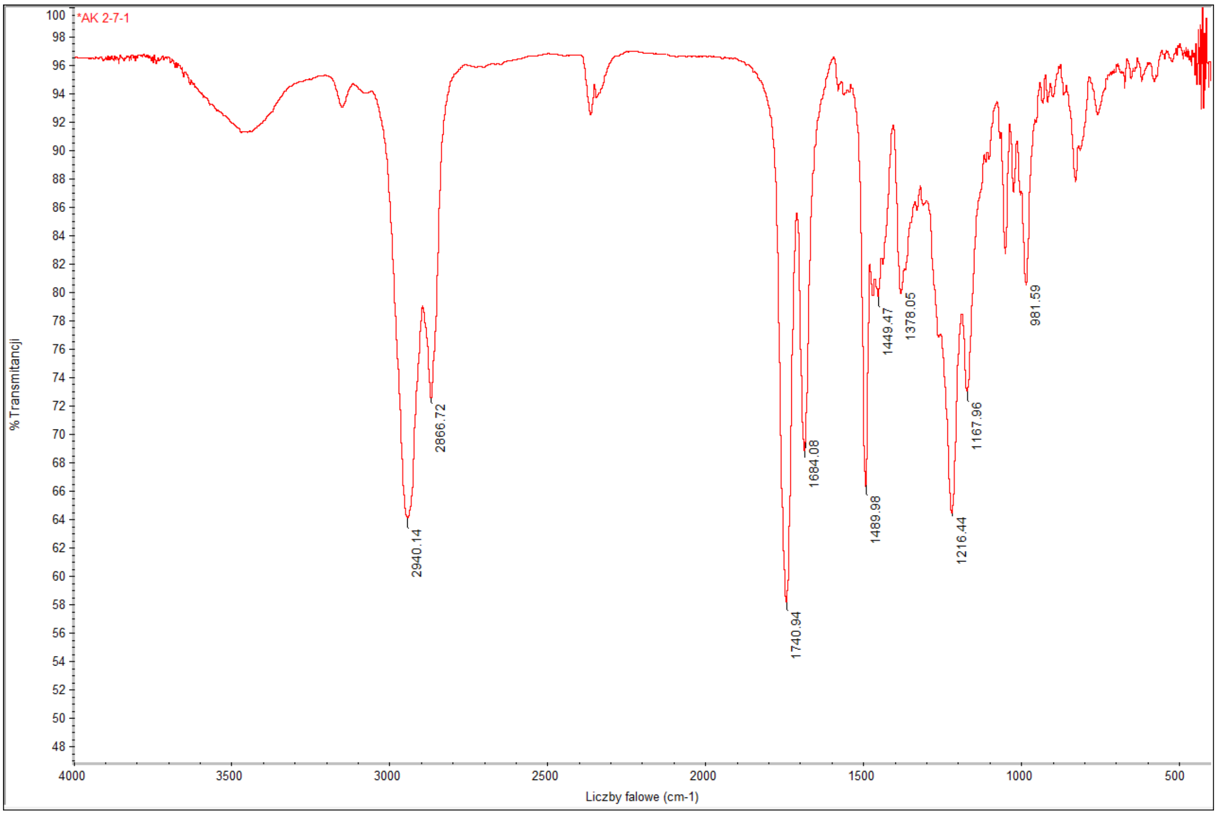
*AK 2-7-1
% Transmitancji
Liczby falowe (cm-1)
2940.14
2866.72
1740.94
1684.08
1489.98
1449.47
1378.05
1216.44
1167.96
981.59

**23**

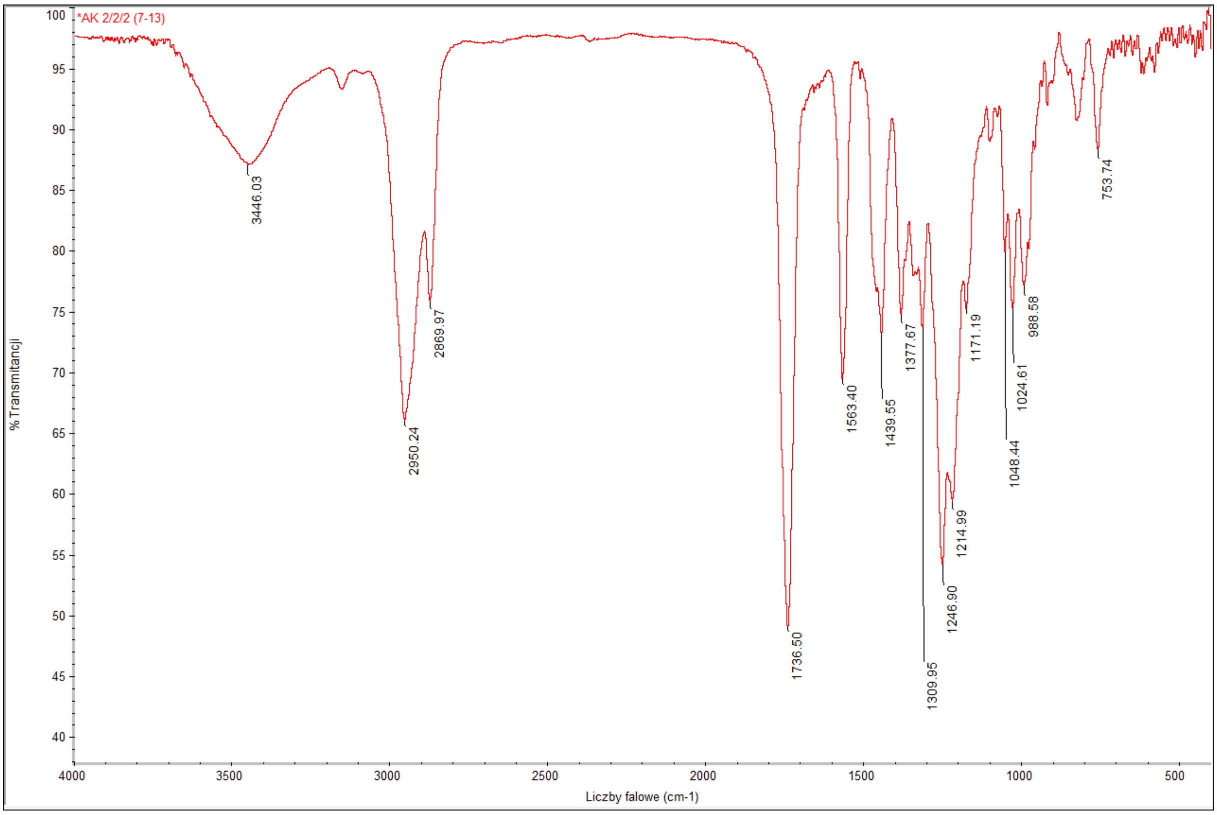
*AK 2/2/2 (7-13)
% Transmitancji
Liczby falowe (cm-1)
3446.03
2950.24
2869.97
1736.50
1563.40
1439.55
1377.67
1309.95
1246.90
1214.99
1171.19
1048.44
1024.61
988.58
753.74

**24**

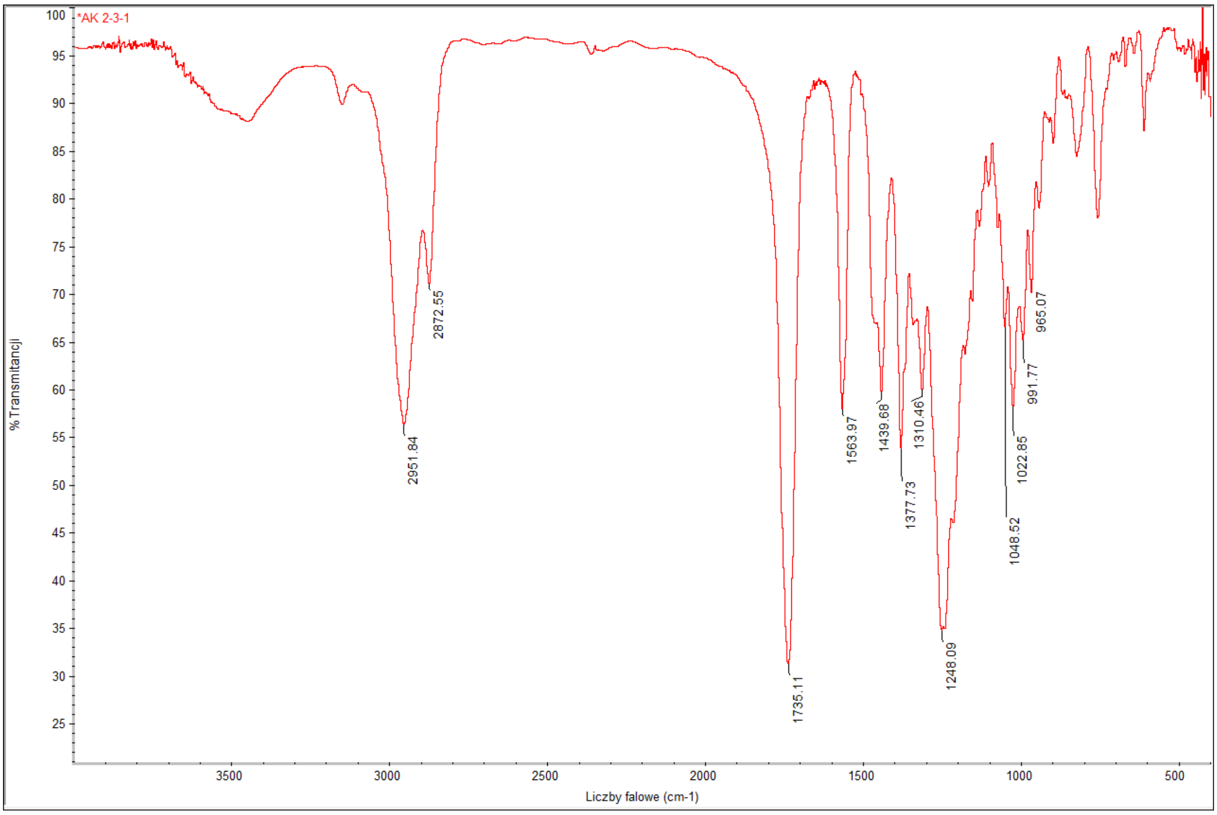
*AK 2-3-1
% Transmitancji
Liczby falowe (cm-1)
2951.84
2872.55
1735.11
1563.97
1439.68
1377.73
1310.46
1248.09
1048.52
1022.85
991.77
965.07

**25**

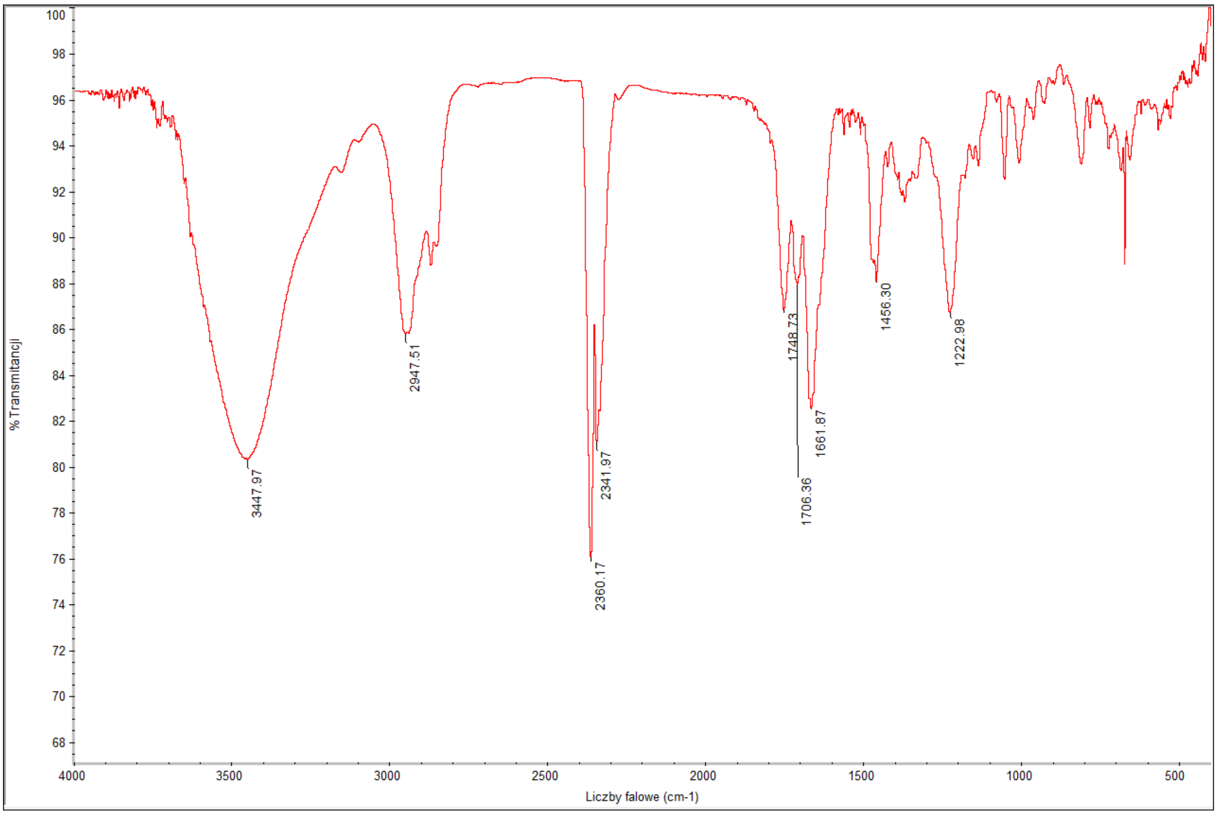
% Transmitancji
Liczby falowe (cm-1)
3447.97
2947.51
2360.17
2341.97
1748.73
1706.36
1661.87
1456.30
1222.98

**26**

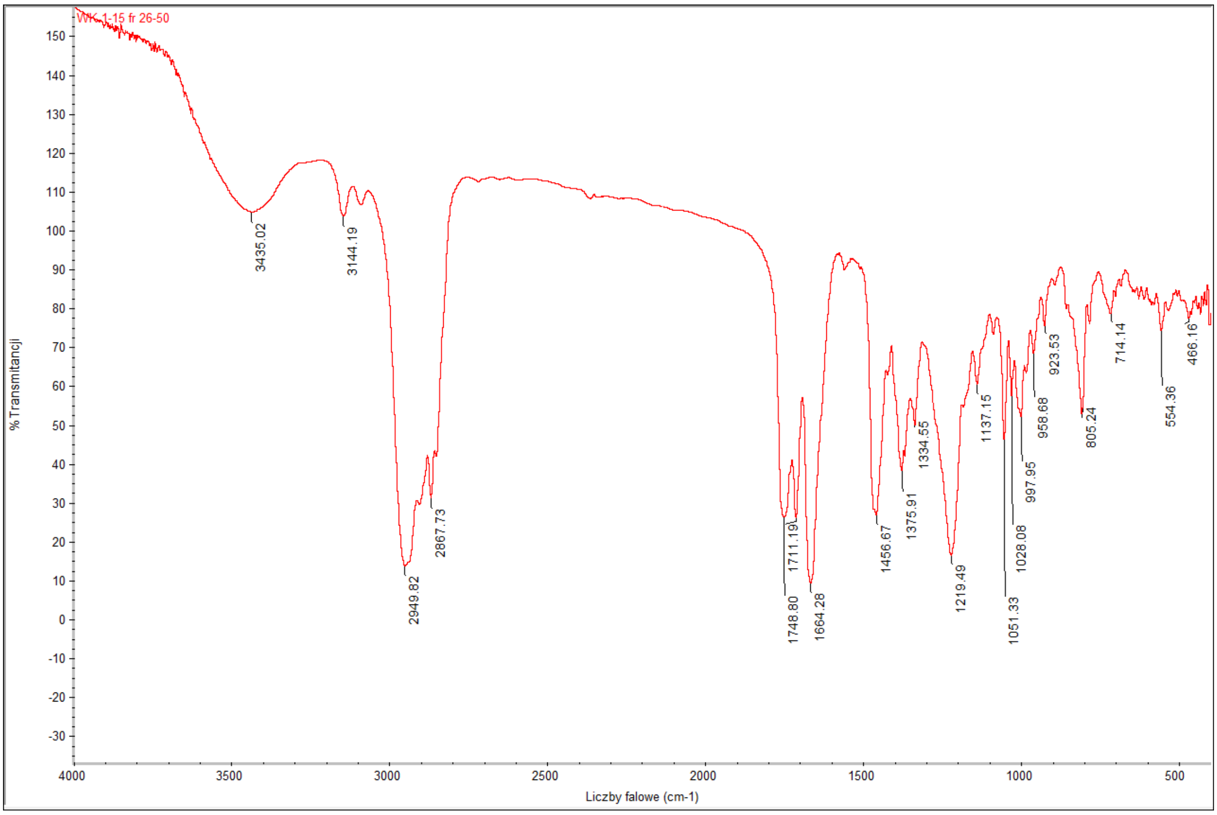

WK 1-15 fr 26-50
% Transmitancji
Liczby falowe (cm-1)
3435.02
3144.19
2949.82
2867.73
1748.80
1711.19
1664.28
1456.67
1375.91
1334.55
1219.49
1137.15
1051.33
1028.08
997.95
958.68
923.53
805.24
714.14
554.36
466.16
